# Randomized trial of planning tools to reduce unhealthy snacking: Implications for health literacy

**DOI:** 10.1371/journal.pone.0209863

**Published:** 2019-01-17

**Authors:** Julie Ayre, Carissa Bonner, Erin Cvejic, Kirsten McCaffery

**Affiliations:** 1 Faculty of Medicine and Health, School of Public Health, Sydney Health Literacy Lab, The University of Sydney, Sydney, NSW, Australia; 2 Faculty of Medicine and Health, School of Public Health, The University of Sydney, Sydney, NSW, Australia; Universite de Bretagne Occidentale, FRANCE

## Abstract

**Objective:**

Guidance to address health literacy often focuses on health education rather than tools to facilitate action, despite action being important for self-management. This study evaluated an online intervention informed by health literate design principles and behavior change theory to reduce unhealthy snacking.

**Methods:**

440 participants were recruited online and randomized to an intervention: 1) Health-literate action plan (guided implementation intention); 2) Standard action plan (self-guided implementation intention); 3) Education (healthy snacking fact-sheet). The primary outcome was self-reported unhealthy snacking. Follow-up was at 1 month.

**Results:**

373 participants (84.8%) completed follow-up. Half the sample had adequate health literacy (52%), and the other half had low (24%) or possibly low (25%) health literacy, as measured by Newest Vital Sign (NVS). At follow-up, lower health literacy was associated with more unhealthy snacks and there was no overall difference between intervention groups. However, participants with lower health literacy who used the health-literate action plan reported less unhealthy snacking compared to the standard action plan; the reverse was true for those with higher health literacy scores (b = 1.7, p = 0.03). People scoring 2 points below the mean NVS (M = 3.4, SD = 2.0) using the health-literate action plan reported eating 8 fewer serves of unhealthy snacks, whereas people scoring 2 points above the mean NVS reported eating 6 more serves of unhealthy snacks using the same tool.

**Conclusions:**

These findings suggest that the universal precautions approach currently recommended for health information may be less effective for facilitating action than tailoring to health literacy level.

**Trial registration:**

ANZCTR identifier: ACTRN12617001194358.

## Introduction

Low health literacy is an important predictor of health inequality that is associated with worse health outcomes, including increased hospitalization, risk factors, chronic disease and mortality risk [1]. One means of addressing this issue is to simplify the information presented in health interventions. Several reviews have indicated that this ‘universal precautions’ approach can mitigate the effects of lower health literacy on comprehension of health information, knowledge, service use, and discrete behaviors (such as screening uptake) without compromising these outcomes for people with higher health literacy [1–4]. The universal precautions approach is also a practical solution in that health literacy strategies can be implemented without needing to identify or target people with lower health literacy.

However, despite evidence that health literacy interventions can improve knowledge about a health issue, it is unclear to what extent this translates to improved behavior change such as self-management [2–5]. This parallels a well-documented challenge in the behavior change literature, the discrepancy between intention and behavior (often termed the ‘intention-behavior gap’) [6]. Several theoretical models have incorporated this gap explicitly by partitioning behavior change into 2 phases: a phase that cultivates motivation and intentions, and a phase that promotes action [7–11]. Important differences between these two phases are highlighted in recent reviews of interventions for self-management behaviors. These have emphasized the importance of strategies used in the action phase, such as self-monitoring, action planning and goal setting [12–17].

This may explain why health literacy interventions do not necessarily result in behavior change. By focusing primarily on education [1], health literacy interventions mostly target motivation and intention. Strategies corresponding to the action phase have received little attention in health literacy guidelines [18–20] and interventions [1, 3, 21, 22]. However, this shortcoming is unlikely to be addressed by existing action strategies as these are typically cognitively demanding. For example, effective self-guided action plans require a high level of self-reflection; individuals must select goals that are challenging but still achievable, that are personally meaningful, and that overcome conflicting motivations [6, 18, 23]. In direct contrast, health literacy strategies are underpinned by efforts to reduce the cognitive demand placed on individuals [24, 25]. Furthermore, health literacy interventions that target chronic disease self-management may benefit from reduced cognitive demand because many chronic diseases are associated with cognitive decline [26–28].

The present study aimed to address this gap in the literature by developing an action plan intervention for a lower health literacy audience. The design incorporated current best practice for health literate design as well as strategies to reduce the cognitive effort required to form and carry out an action plan.

A key candidate action plan strategy is ‘implementation intentions.’ This strategy guides individuals to specify the critical cues to carry out a behavioral response, often presented as an ‘if-then’ plan (for example, “If [cue] occurs, then carry out [behavioral response]”) [6, 29]. Systematic reviews have indicated that these are effective at improving a range of lifestyle behaviors in the general population [30–32]. They may also be particularly useful for people with lower health literacy as some have argued that less cognitive effort is needed to carry out these plans [33–36].

However, it can be difficult to create effective implementation intentions. Gollwitzer, Wieber [37] identified several criteria for effective implementation intentions, including strong behavioral intentions, an easily recognizable and frequently occurring cue, and a behavioral response that contributes to the goal and is neither too challenging nor too easy. The ‘volitional help sheet’ [38] is a specific type of implementation intention tool that can increase the likelihood that these criteria are met. This tool guides the user to select the most relevant ‘if’ and ‘then’ statements from a predetermined list of cues and responses. Studies have also shown that this tool is effective for lifestyle behavior change [39–44].

The current study investigated the effectiveness of an online action plan intervention to reduce unhealthy snacking. High intake of discretionary foods (i.e. ‘snacks’ including cakes, biscuits, chocolate and confectionary) has been identified as an important contributor to unhealthy diets in Australia and worldwide [45]. This intervention was designed to meet current best practice guidelines for online health literacy interventions, such as simple language and use of white space [19]. Plan formation was facilitated through an online volitional help sheet. This was compared to two controls: a standard action plan (a standard implementation intention) and education (a fact sheet on healthy snacking). It was hypothesized that health literacy would moderate the effect of the interventions on unhealthy snacking with lower literacy participants showing better outcomes using the health-literate action plan design

## Materials and methods

### Study design

This between-subjects randomized trial compared the effects of three interventions on unhealthy snacking over one month. This study was approved by the University of Sydney Human Research Ethics Committee [2017/622]. The study was prospectively registered with ANZCTR (www.anzctr.org.au) (ACTRN12617001194358).

### Participants

The market research company Research Now Survey Sampling International independently recruited Australian adults (N = 440) to participate in an online survey in return for points that can be redeemed for gift vouchers. Quota sampling ensured representation by lower educated participants with 50% of the sample with less than university education, and equal numbers of men and women. Inclusion criteria were fluency in English and age over 30 years to maintain relevance to chronic and lifestyle conditions such as type 2 diabetes. Participants were not selected on the basis of current snacking behavior or BMI.

### Procedure

**Fig 1:**
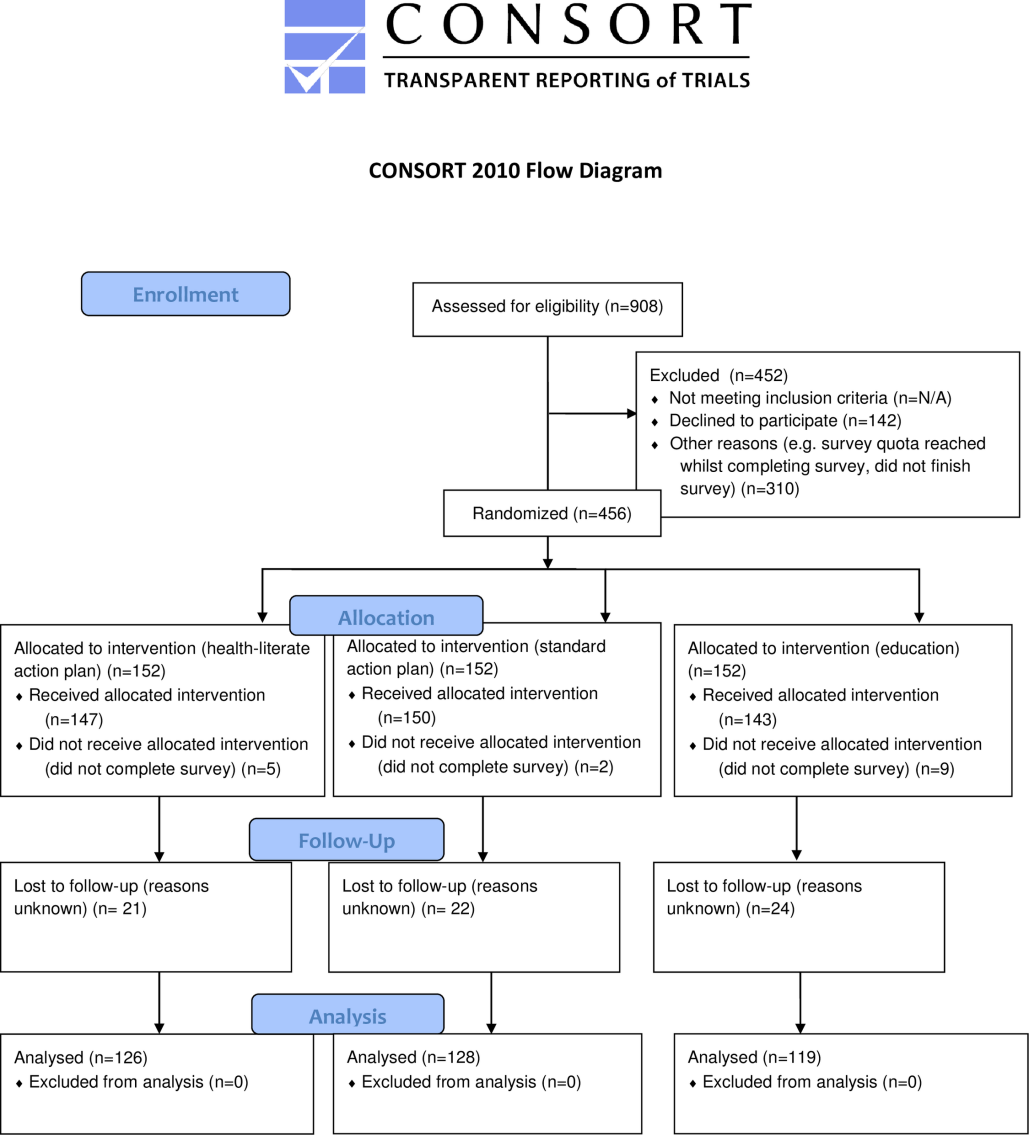
CONSORT flow diagram.

**Fig 2:**
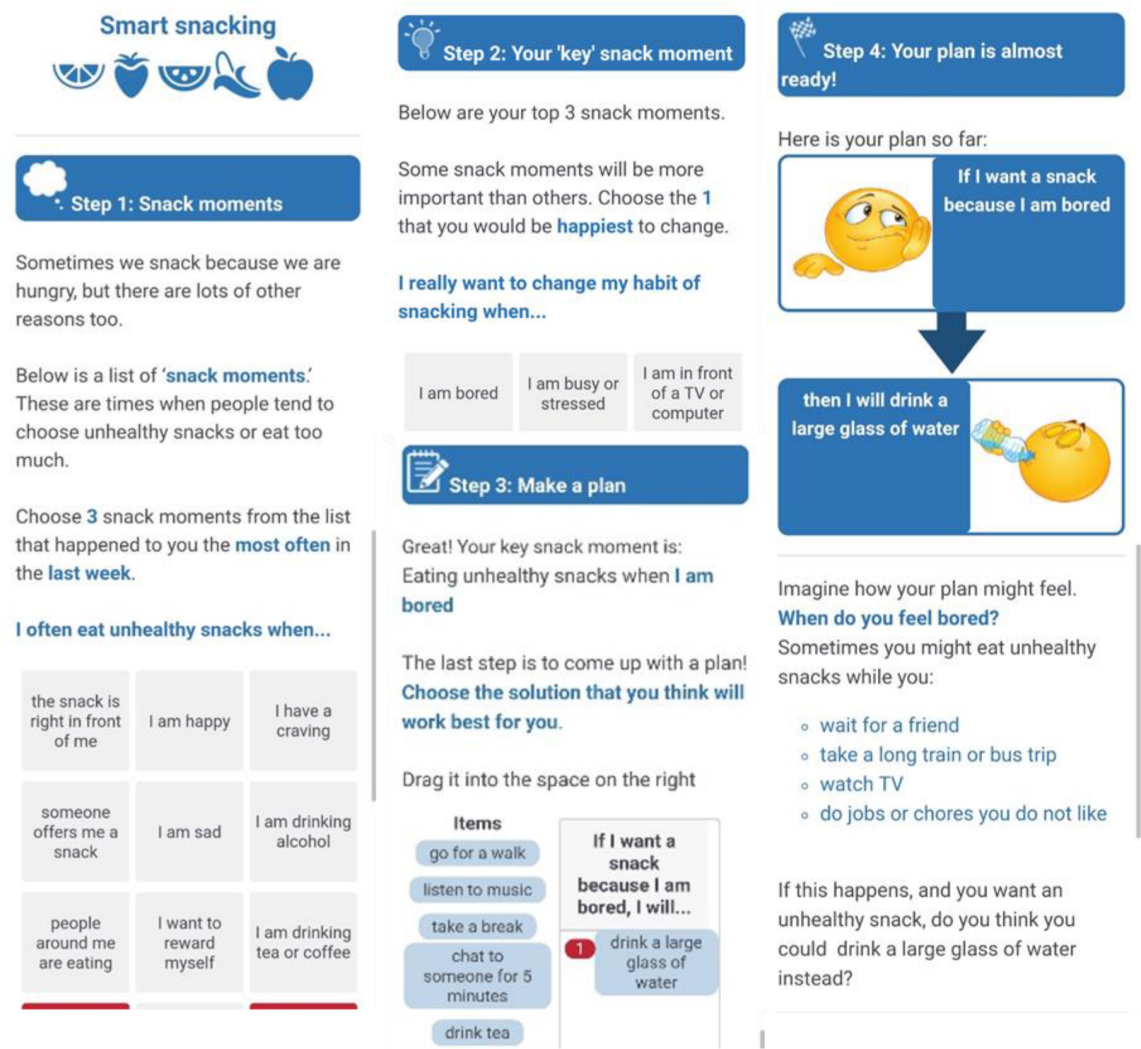
Mobile screenshots from *health-literate action plan*. From left to right: a) Step 1: Selecting top 3 snacking scenarios; b) Step 2: Selecting 1 key scenario; c) Step 3: Selecting a solution; d) Step 4: Imagining the plan.

Surveys were hosted by Qualtrics. After completing informed consent, demographics and baseline measures, participants were automatically randomized evenly to an intervention group (see Box 1 and Figs 1 and 2) using the Qualtrics ‘randomizer,’ and asked post-intervention to make a digital or paper copy of their plan. Participants were blind to the intervention to which they were randomized. Participants received emails containing their plan at baseline, 1 and 2 weeks. Participants completed the follow-up survey at 4 weeks.

**Box 1: Description of interventions**

**Health-literate action plan**

Description:

1. Participants chose 3 scenarios they found most difficult to eat healthily (Panel a, Fig 2). The list of scenarios was based on previous studies [43, 44], adapted for unhealthy snacking. Participants then selected the scenario they were most motivated to address (Panel b, Fig 2).
2. Participants dragged an action from a list and placed it underneath the scenario (Panel c, Fig 2). This created the implementation intention i.e. “If [scenario], then I will [action].” Participants were asked to imagine the plan (Panel d, Fig 2) and rate how difficult they would find it over the next month using a 10-point scale (Very easy to very hard). Those scoring 7 or more were asked to revise their action.

Active elements:

- Implementation intention (focus on cues for action)
- High quality plan (Cue is likely to be high frequency and personally relevant, solution is not too challenging)
- Health-literate design for comprehension, including minimal and simple text, illustrative images and white space [19].

**Standard action plan**

Description:

Participants read instructions similar to those used by Armitage [40]:

“We want you to plan how you will change your unhealthy snacking behavior each day because forming plans has been shown to improve snacking habits. You are free to choose how you do this but we want you to formulate your plans in as much detail as possible. Please pay attention to the situations in which you will implement these plans. Focus on situations when you are not hungry but find yourself snacking.”

Active elements:

- Implementation intention (focus on cues for action)

**Education**

Description:

The fact sheet was based on the Australian National Diabetes Services Scheme ‘Healthy snacks’ fact sheet [46], with references to diabetes, carbohydrates and blood glucose removed.

Active elements:

- Information about healthy snacking options

### Measures

#### Health literacy

Health literacy measures included the Newest Vital Sign (NVS) [47], a 6-item measure of functional health literacy. NVS scores of 0–1 indicate a high likelihood of limited healthy literacy, scores of 2–3 indicate the possibility of limited health literacy and scores of 4–6 indicate adequate health literacy (Cronbach’s α = 0.78).

#### Snack scores (previous month)

A 7-item measure of snacking was based on a diet score developed and validated by a well-established and highly regarded Australian scientific organization (CSIRO) [45]. Items were drawn from the ‘discretionary foods’ category which the Australian Guidelines to Healthy Eating define as 'not considered necessary for a healthy diet’. Alcohol and sugar sweetened beverages were excluded from the assessment in this study as the focus was on ‘snacks.’ Participants answered how many serves of unhealthy snacks they ate in the past month. Participants could answer according to the number of serves per day, week or month. Average weekly servings of unhealthy snacks were calculated from these scores.

#### Cognitive variables

Cognitive variables with theoretical importance for the action phase of behavior change were included [7]. Items were based on existing measures of self-efficacy [7], planning [7], action control [48], intentions [49, 50] and habit strength [51] and adapted to refer specifically to unhealthy snacking. These were measured at baseline and follow-up, with the exception of habit strength (baseline only) and action control (follow-up only). Responses were recorded on 7-point Likert scales (strongly disagree to strongly agree).

#### Difficulty using the planning tool

A single item asked participants to rate how hard it was to use the planning tool (1 = not at all hard, 5 = extremely hard).

### Analysis

#### Sample size

A sample size estimate of 261 was based on 90% power at α = 0.05 to detect a moderate effect size (Cohen’s f = 0.25, corresponding to ANCOVA effects and interaction as small as ηp ^2^ = 0.06) in the primary outcome (unhealthy snacking score). With an anticipated attrition rate of 40%, this totaled 435 participants. Effect size estimate was based on a previous analysis a of volitional help sheet intervention [40].

#### Analysis methods

Randomization at baseline was assessed using ANOVA (or Kruskal-Wallis H test for non-normal data) for continuous variables and chi-square tests for categorical variables. Multiple linear regression was used to predict snacking scores and perceived difficulty using the plan at follow-up. Contrasts compared participants allocated to a) the health-literate and standard action plans; and b) the latter two plans and education. Important correlates of health literacy (age, level of education, language spoken at home) [1], baseline snacking and health literacy (as NVS scores) were controlled for in the model, including an intervention group × health literacy interaction term. NVS scores were examined both continuously and categorically. To assist with interpretation of the effect on snacking behavior, the effect of NVS scores were expressed in 2-point increments as this corresponds to a meaningful shift in health literacy category [47].

Each cognitive variable (baseline habit strength; follow-up intention, action control, planning and self-efficacy) was independently assessed as a potential mediator of the effect of intervention group and health literacy on snacking behavior. Mediation was evaluated using Hayes [52]’ mediation method (PROCESS V3.0). Each assessment of mediation for follow-up cognitive variables included the corresponding baseline measure as a covariate where available), as well as baseline snacking, age, level of education and language spoken at home. Model 4 was employed for main effects (a mediation model testing the direct effect of intervention group and health literacy on snack scores, the effect of the potential mediator on snack scores, and the indirect effect of intervention group and healthy literacy on snack scores via the potential mediator), and Model 8 was employed for interaction effects, similar to Model 4 but with health literacy included as the moderator of the effect of intervention group on snack scores. Mediation was evaluated using 95% confidence intervals generated from 5,000 bootstrapped samples. For contrasts, only the relevant groups were included in the mediation analysis.

Two researchers independently coded standard action plans to indicate the extent that participants followed standard action plan instructions and the extent that plans differed from the pre-determined options presented in the health-literate action plans. Coders were blind to the health literacy level of participants. Any disagreements were resolved through discussion.

## Results

Three hundred and seventy-three of the 440 participants (84.8%) completed the follow-up survey (Fig 1). Descriptive statistics are shown in Tables 1–3. There were no significant differences in age, gender, BMI, intention, habit strength, NVS score and baseline snacking scores across intervention groups at baseline.

**Table 1:** Baseline participant characteristics.

Demographic variables	N	%
Age (years)		
≤ 40	124	33.2
41–50	81	21.7
51–60	85	22.8
> 60	83	22.3
Female	190	50.1
Speaks English at home	354	94.9
Education		
Less than high school education	15	4.0
High school graduate	63	16.9
Certificate	80	21.4
University education	215	57.6
Health literacy (NVS categories)		
High likelihood of limited health literacy (scores 0–1)	88	23.6
Possibly limited health literacy (scores 2–3)	92	24.6
Adequate health literacy (scores 4–6)	193	51.7
Self-reported BMI (kg/m^2^)	218	58.4
Underweight (<18.5)	8	2.1
Normal weight (18.5–24.9)	147	39.4
Overweight (25.0–29.9)	116	31.1
Obese (≥ 30.0)	102	27.3

*Note*. BMI: Body mass index; NVS: Newest Vital Sign

**Table 2:** Average unhealthy snack serves per week at baseline and follow-up, by intervention group.

Intervention group	N	Baseline snack score		Follow-up snack score	
		Mean	SD	Mean	SD
Health-literate action plan	126	20.2	28.3	18.6	28.2
Standard action plan	128	15.5	14.8	16.4	34.0
Education	119	12.5	10.8	11.9	14.6
**Total**	**373**	**16.2**	**19.8**	**15.7**	**27.1**

**Table 3:** Baseline cognitive variables, by intervention group.

Cognitive variables (scale range)	Health-literate action plan		Standard action plan		Education		Total	
	Mean	SD	Mean	SD	Mean	SD	Mean	SD
Intention (1 low– 7 high)	5.0	1.6	5.3	1.2	5.2	1.3	5.1	1.4
Habit strength (1 low– 7 high)	3.7	1.5	3.9	1.4	3.6	1.4	3.7	1.4
Planning (1 low– 7 high)	4.3	1.4	4.5	1.3	4.5	1.3	4.4	1.5
Self-efficacy (1 low– 7 high)	4.5	1.0	4.6	1.0	4.6	0.9	4.5	1.0

At baseline, participants scored around the midpoint of all cognitive variables (habit strength, planning and self-efficacy). Intention scores were on average slightly higher, at approximately 5 out of 7, indicating a moderately positive intention (Table 3).

### Health-literate action plan

Table 4 shows the frequency of selected health-literate action plan scenarios and solutions. Twenty-eight participants (22%) revised their solution after being advised to choose an easier plan (See S2 File for examples).

**Table 4:** Characteristics of 'if statements' and ‘then statements’ selected by participants using the health-literate action plan.

Scenario selected for ‘if statement’	N	%	Solution selected for ‘then statement’	N	%
I have a craving	24	19.0	drink a large glass of water	24	19.0
I am bored	16	12.7	eat a piece of fruit	23	18.3
I am in front of a TV or computer	15	11.9	go for a walk	14	11.1
I start with one piece but then keep eating	10	7.9	drink tea	11	8.7
the snack is right in front of me	8	6.3	eat a smaller amount	11	8.7
I want to reward myself	8	6.3	chat to someone for 5 minutes	10	7.9
I am busy or stressed	7	5.6	do a chore or task	10	7.9
I am happy	7	5.6	eat fresh vegetables and dip	9	7.1
I am sad	7	5.6	Other solutions with frequency <5%†	14	11.1
Other scenarios with frequency <5% *	24	19.0			

*Note*.

*’Other scenarios with frequency <5%’: ‘people around me are eating’, ‘I am drinking tea or coffee’, ‘I am drinking alcohol’, ‘it is part of a celebration or special event’, ‘I have arrived home’, ‘someone offers me a snack’, and ‘I am about to go to bed.’

†’Other solutions with frequency <5%’ include: ‘listen to music,’ ‘take the food out of the packet and put it on a plate,’ ‘move the snack into the cupboard,’ ‘politely say “No thank you”,’ and ‘take a break.’

### Standard action plan

Average plan length was 23.3 words (SD = 20.0). This varied by health literacy level. Plan length was 24.0 words (SD = 19.9) for participants with adequate health literacy, and 10.3 words (SD = 12.0) for participants with high likelihood of limited health literacy. One third of participants with high likelihood of limited health literacy did not create a plan, compared to 13% of participants with adequate health literacy (Table 5).

**Table 5:** Characteristics of standard action plans by health literacy level.

Plan characteristic	High likelihood of lower health literacy		Possibility of lower health literacy		Adequate health literacy	
	N	%	N	%	N	%
No plan created*	10	33.3	6	21.4	9	12.9
Plan was not specific (e.g. "eat less junk food")*	8	26.7	7	25.0	9	12.9
Plan identified a trigger situation for unhealthy snacking	2	6.7	6	21.4	21	30.0
Plan identified a solution for unhealthy snacking	11	36.7	15	53.6	52	74.3
Plan involved changing the existing environment	3	10.0	9	32.1	25	35.7
Plan identified trigger situation not listed in health-literate action plan	1	3.3	3	10.7	8	11.4
Plan identified a personal solution not presented in health-literate action plan	3	10.0	9	32.1	34	48.6
*Eat only a designated unhealthy snack*	2	6.7	1	3.6	3	4.3
*Do not buy unhealthy snacks*	1	3.3	7	25.0	12	17.1
*Do not keep unhealthy snacks at home*	1	3.3	2	7.1	11	15.7
*Eat larger main meals / do not skip meals*	1	3.3	0	0.0	6	8.6
*Plan included having healthy snacks ready and available*	0	0.0	1	3.6	17	24.3
**Total number of participants**	**30**		**28**		**70**	

*Note*.

*These categories were mutually exclusive.

### Perceived difficulty using the interventions

On average, participants reported the interventions were easy to use (M = 1.5, SD = 0.8), although participants with lower health literacy reported finding them more difficult to use (b = -0.12, SE = 0.02, *p*<0.01). The education intervention was rated more difficult to use than both of the action plans (b = 0.13, SE = 0.06, *p* = 0.03).

### Impact of interventions on snacking behavior

The overall model was significant (F_(9, 363)_ = 9.0, *p*<0.001), explaining 18.3% of variance in snack diary scores. Of the explained variance, 15.5% can be attributed to health literacy and intervention group (ΔR^2^ from base model = 0.03, F_(5, 363)_ = 2.5, *p* = 0.03). Overall this represented a small effect on unhealthy snacking [53]. There was no main effect of intervention group. The model suggested that NVS score significantly predicted snack scores, controlling for age, language spoken at home, education and baseline snacking score, such that for each point increase on the NVS (i.e. higher health literacy), unhealthy snacks per week were expected to decrease by 1.65 serves (SE = 0.67, *p* = 0.01) (Table 6).

**Table 6:** Multiple linear regression model predicting snack serves per week.

Predictors	B	95% CI	P value
Intercept	15.91	13.37–18.46	<0.01
Age (years)	-0.18	-0.39–0.03	0.10
English spoken at home	-4.69	-16.58–7.20	0.44
Education	4.14	-1.04–9.33	0.12
Baseline snack score	0.49	0.36–0.62	<0.01
NVS score	-1.65	-2.98–-0.33	0.01
Contrast 1: health-literate action plan vs standard action plan	-0.38	-3.49–2.72	0.81
Contrast 2: health-literate action plan/standard action plan vs education	-2.17	-5.84–1.50	0.25
Contrast 1*NVS score	1.74	0.20–3.28	0.03
Contrast 2*NVS score	0.51	-1.35–2.37	0.59

*Note*. NVS: Newest Vital Sign

**Fig 3:**
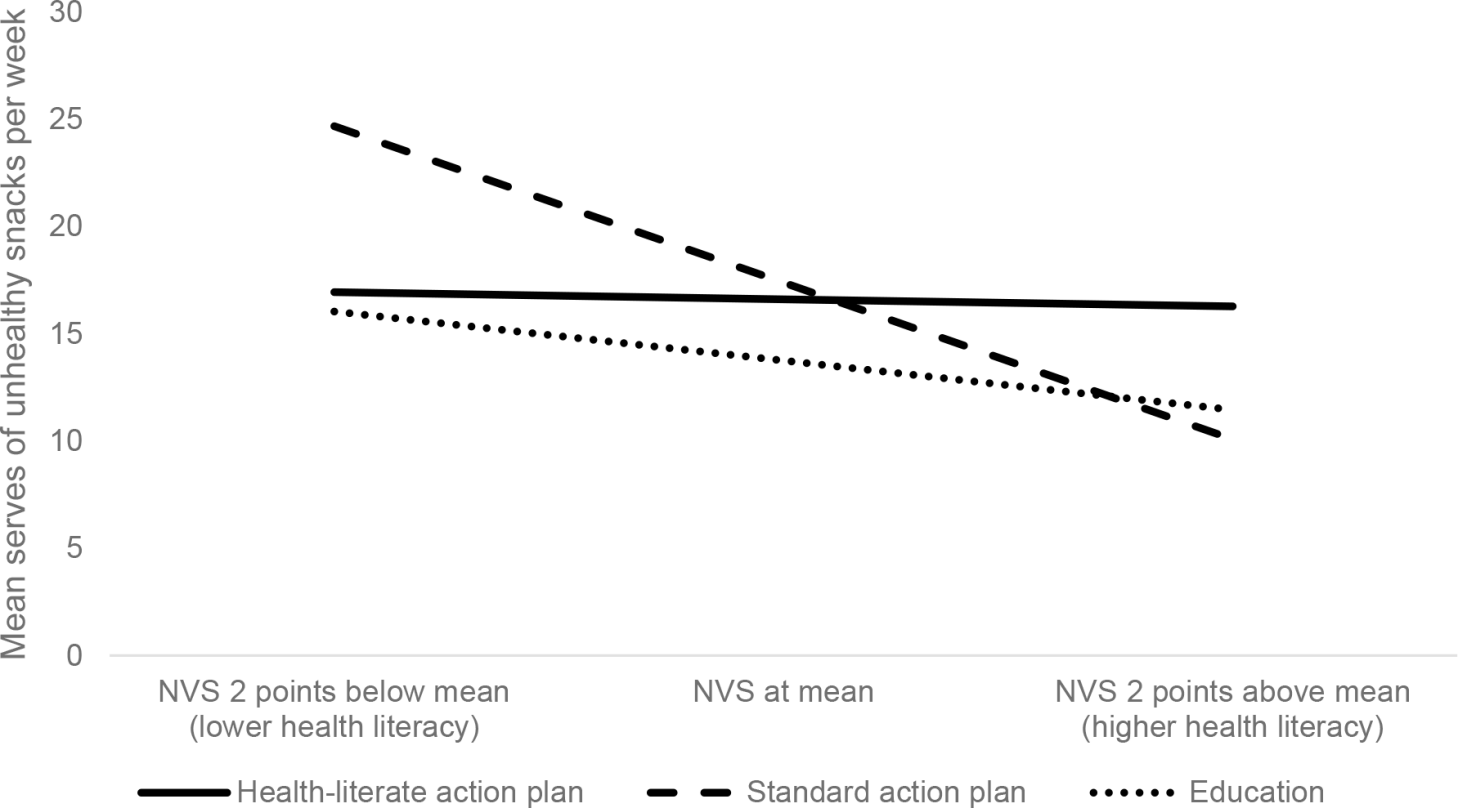
Predicted unhealthy snack serves per week by intervention group and health literacy score*. *Analysis controlled for age, language spoken at home, education and baseline snacking score. *Note*. NVS: Newest Vital Sign.

There was a significant interaction between NVS scores and the health-literate and standard action plan groups (b = 1.74, SE = 0.78, *p* = 0.03). As shown in Fig 3, this suggests that participants with lower health literacy benefited more from the health-literate action plan compared to those using the standard action plan. For example, a person scoring 2 points below the mean on the NVS would be expected to eat 7.7 fewer serves of unhealthy snacks per week using the health-literate action plan rather than the standard version. By contrast, participants with higher health literacy had significantly lower snack scores when they used the standard action plan, such that a person who scored 2 points above the mean on the NVS would eat 6.2 *fewer* serves of unhealthy snacks per week using the standard action plan, as compared to the health-literate action plan.

Cognitive variables were assessed as potential mediators for: 1) the main effect of health literacy on snacking; and 2) the interaction between intervention group contrast 1 (health-literate action plan vs standard action plan) and health literacy. Neither health literacy nor the intervention group contrast predicted scores on cognitive variables at follow-up. Furthermore, there was no statistical evidence that the cognitive variables mediated the effects of health literacy and intervention group on snacking scores.

## Discussion

This study found that lower health literacy scores predicted greater consumption of unhealthy snacks after 1 month of using an online intervention. This effect was moderated by the type of intervention. People with lower health literacy ate fewer serves of unhealthy snacks when they used a health-literate action plan rather than the standard version. Conversely, people with higher health literacy ate fewer unhealthy snacks when they used the standard action plan rather than health-literate version. This study did not find evidence of mediation by cognitive variables (habit strength, intention, self-efficacy, planning or action control) of the effect of health literacy and intervention group on self-reported snacking scores at follow-up.

This study opens up an important discussion about how the universal precautions approach to health literacy (that is, using a lower health literacy design for all consumers) might apply to self-management behavior change interventions. Whilst numerous studies have found that the universal precautions approach offers benefits to people with lower health literacy without compromising outcomes for people with higher health literacy [1–4], this research has largely focused on information comprehension, recall, acceptability of the intervention and implications for service use and cost [1, 2]. Comparatively few studies have investigated the effect of health-literate design on the *action phase* of behavior change interventions. Furthermore, no studies have specifically investigated the effects of health-literate design on action plans which seek to reduce the intention-behavior gap.

The findings from the present study suggested that the universal precautions approach may adversely impact outcomes for people with higher health literacy. This highlights that education and action may not operate via the same mechanisms despite both being common components of health interventions. The universal precautions approach that is applied to education is underpinned by the principle of reducing cognitive demand. By contrast, action plans are inherently cognitively demanding because of the high level of self-reflection required [18, 23]. Goal setting theories also argue that cognitive effort can actually facilitate engagement as long it is not *too* challenging [23].

In the present study the health-literate action plan facilitated plan formation with the aim of reducing cognitive demand compared to the standard version. For example, the planning process was broken down into steps, users were restricted to a single plan, and there was no option for free text. Participants with lower health literacy benefited from this approach. Not only were their reported snacking scores lower using the health-literate action plan, but one third of participants with a high likelihood of inadequate health literacy did not create a plan, and almost one quarter created plans that lacked specificity.

**Fig 4:**
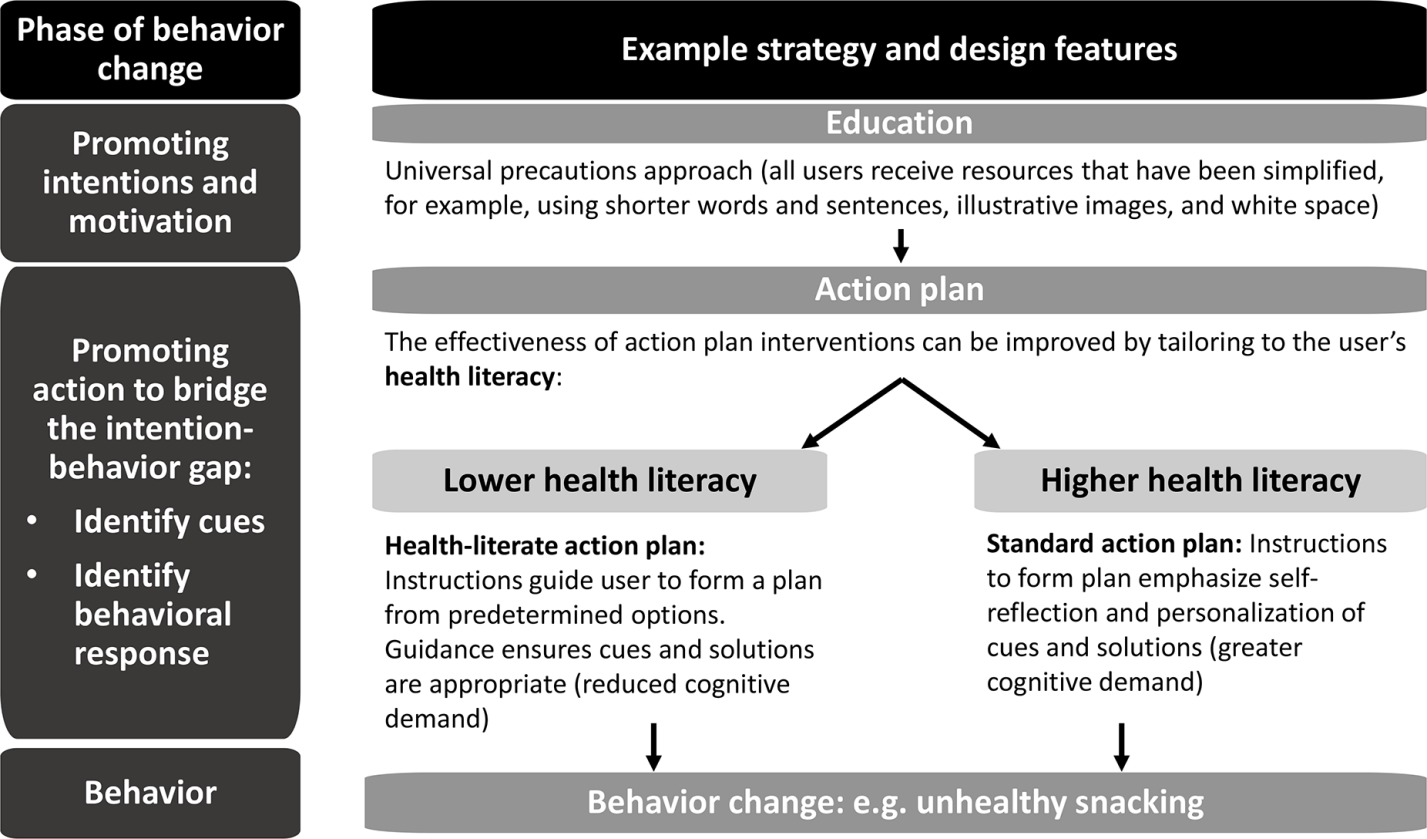
Model of recommended approach to behaviour change interventions in populations with diverse health literacy.

By comparison, participants with higher health literacy may have felt restricted by the options available in the health-literate action plan and were able to adequately generate their own effective plans using the standard version. For example, many of these plans included strategies that were not explicitly included in the health-literate action plan, such as preparing healthy snacks ahead of time so that they would be ready and available to eat. Together, these results for participants with lower and higher health literacy support the idea that tailoring intervention components that focus on the action phase may be a more effective way to promote lifestyle behavior change in diverse populations (Fig 4).

### Strengths

Key strengths of this study were the rigorous randomized controlled design, high retention, and strong generalizability (the standard action plan was similar to widely used action plan tools) [18, 54]. This study also sampled participants aged at least 30 years and of varying BMI and health literacy levels, providing evidence that online self-management tools can be appropriate for target populations for chronic disease prevention and self-management.

### Limitations

A limitation was that the snack measure asked participants to self-report food consumed over the previous month. This was mitigated by using a validated dietary measure [45] that asked about specific categories of foods to assist recall. Randomization also reduced the risk of differential self-reported snacking across intervention groups.

It should also be noted that all participants who used the health-literate action plan had to create a plan, whereas participants using the standard action plan could submit text that did not follow the instructions. As such, it is possible that the mandatory creation of plans may also have contributed to the effectiveness of the health-literate action plan.

### Future directions

This study provides a starting point for investigating how health literate design can best be applied to action plans for lifestyle change. For example, future research should investigate whether simply incorporating additional options into the health-literate action plan would improve its effectiveness for people with higher health literacy. It would also be useful to investigate whether individuals can reliably self-select the type of action plan for their health literacy level, or whether a screening item for health literacy could adequately fulfil this purpose.

## Conclusions

This study addressed an important gap in the literature by examining how an action plan intervention can be adapted for a lower health literacy audience to improve self-management (reducing unhealthy snacking). The results indicated that the effectiveness of the intervention was dependent on the health literacy of the participant; participants with lower health literacy benefited from health-literate design in which cognitive demand was reduced, whereas users with higher health literacy benefited from a design that gave them freedom to generate their own detailed and highly personalized plans. These findings have two key implications: (1) health literacy interventions that target people with lower health literacy should aim to reduce the cognitive demand required to *create* action plans; and (2) people with higher and lower health literacy may need different strategies for creating effective action plans.

## Supporting information

S1 File CONSORT checklist. (DOC)

S2 File Appendix (Additional tables). (DOCX)

S3 File Survey. (PDF)

S4 File Data and data dictionary. (XLSX)

S5 File Trial protocol (ethics). (DOCX)

S6 File Documentation submitted to ethics committee. (PDF)
