# Supplementary material for: Randomized trial of planning tools to reduce unhealthy snacking: Implications for health literacy
Source: PLoS One. 2019 Jan 17;14(1):e0209863. doi: 10.1371/journal.pone.0209863 (PMC6336265; doi:10.1371/journal.pone.0209863)
Supplement: S2 File — (DOCX) [file pone.0209863.s002.docx]

**S2 Appendix (Additional tables)**

**Table S1: Baseline participant characteristics, by intervention group**

|  | **Health-literate action plan** | | **Standard action plan** | | **Education** | |
| --- | --- | --- | --- | --- | --- | --- |
| **Demographic variables** | **N** | **%** | **N** | **%** | **N** | **%** |
| Age (years) |  |  |  |  |  |  |
| ≤ 40 | 40 | 31.7 | 42 | 32.8 | 42 | 35.3 |
| 41-50 | 26 | 20.6 | 27 | 21.1 | 28 | 23.5 |
| 51-60 | 33 | 26.2 | 35 | 27.3 | 17 | 14.3 |
| > 60 | 27 | 21.4 | 24 | 18.8 | 32 | 26.9 |
| Female | 64 | 50.8 | 64 | 50.0 | 62 | 52.1 |
| Speaks English at home | 120 | 95.2 | 119 | 93.0 | 115 | 96.6 |
| Education |  |  |  |  |  |  |
| Less than high school education | 6 | 4.8 | 5 | 3.9 | 4 | 3.4 |
| High school graduate | 22 | 17.5 | 18 | 14.1 | 23 | 19.3 |
| Certificate | 27 | 21.4 | 30 | 23.5 | 23 | 19.3 |
| University education | 71 | 56.4 | 75 | 58.6 | 69 | 58.0 |
| Health literacy (NVS categories) |  |  |  |  |  |  |
| High likelihood of limited health literacy (scores 0-1) | 35 | 27.8 | 30 | 23.4 | 23 | 19.3 |
| Possibly limited health literacy (scores 2-3) | 33 | 26.2 | 28 | 21.9 | 31 | 26.1 |
| Adequate health literacy (scores 4-6) | 58 | 46.0 | 70 | 54.7 | 65 | 54.6 |
| Self-reported BMI (kg/m^2^) |  |  |  |  |  |  |
| Underweight (<18.5) | 2 | 1.6 | 3 | 2.3 | 3 | 2.5 |
| Normal weight (18.5-24.9) | 44 | 34.9 | 48 | 37.5 | 55 | 46.2 |
| Overweight (25.0-29.9) | 42 | 33.3 | 43 | 33.6 | 31 | 26.1 |
| Obese (≥ 30.0) | 38 | 30.2 | 34 | 26.6 | 30 | 25.2 |
| **Total** | **126** |  | **128** |  | **119** |  |

*Note.* BMI: Body mass index; NVS: Newest Vital Sign

**Examples of plan revisions in the health-literate action plan**

Table S2 provides examples of how participants revised if-then plans when the original plan was considered difficult (a score of 7 or more out of 10). 35 plans were revised (although 7 participants ultimately selected the same plan the second time). Of the remaining 28 revisions, the most common ‘then’ solution to be revised was ‘…then I will eat a piece of fruit’ (N=7) and ‘then I will go for a walk’ (N=6). The most common replacement ‘then’ solutions were ‘…then I will chat to someone for 5 minutes’ (N=6) and ‘…then I will drink a large glass of water’ (N=5).

**Table S2: Examples of health-literate action plan revisions**

| **‘If’ scenario** | **‘then’ solution** | **Revised ‘then’ solution** |
| --- | --- | --- |
| If I am in front of a TV or computer | Then I will eat a piece of fruit | Then I will drink a large glass of water |
| If I have a craving | Then I will eat a smaller amount | Then I will drink tea |
| If I have a craving | Then I will eat a piece of fruit | Then I will drink a large glass of water |
| If I start with one piece and then keep eating | Then I will go for a walk | Then I will chat to someone for 5 minutes |

**Examples of standard action plans from participants with low and high health literacy**

Table S3 provides examples plans created by participants with low and high health literacy who used the standard action plan.

**Table S3: Examples of standard action plans, low and high health literacy**

| **High likelihood of inadequate health literacy** | **Adequate health literacy** |
| --- | --- |
| “Avoid snacks and drink more water and fruit”  “Don't buy snack food”  “Eat more fruit & healthier snacks If i feel down or lonely will go for walk or exercise Drink more water”  “Prepare all meals at home. BREAKFAST -LUNCH AND DINNER as per existing plan. Yoghurt for morning tea and fresh strawberries for supper. Gym on Mondays Tuesdays Thursdays and Fridays.” | “Ask my wife not to buy biscuits or chocolate so there are none in the house. Ask my wife to buy more fruit to substitute.”  “Biggest risk is the table I regularly walk past at work that has chocolates, lollies and chips. I always (habitually) stop and get some even when I'm not hungry and I know I shouldn't have them. Sometimes I buy bad foods when they’re on special, so I’ll eat them when I'm home even when I'm not hungry, or as a meal replacement”  “Eat a banana a day. Buy nuts dried apricots sultanas Blueberries. Strawberries. Smoothies - get blender going daily. No food in bedroom.”  “Eat more carrot sticks and fruit”  “Fruit for morning tea with coffee with skim milk. Nuts & dried fruit for afternoon tea with coffee and skim milk. Coffee with skim milk and low calorie biscuit for supper.”  “I plan not to eat a whole family block of chocolate in one go. I will keep it in the kitchen instead of in the lounge room”  “When I want to snack, I will either drink water first or I would eat grapes. Not snacking is doable since I do not have snacks in the house since I can't afford them.” |

**Table S4: Dates of recruitment**

|  | **From** | **To** |
| --- | --- | --- |
| **Baseline** | 14/09/2017 | 22/10/2017 |
| **Follow-up** | 12/10/2017 | 27/11/2017 |
