# Supplementary material for: Randomized trial of planning tools to reduce unhealthy snacking: Implications for health literacy
Source: PLoS One. 2019 Jan 17;14(1):e0209863. doi: 10.1371/journal.pone.0209863 (PMC6336265; doi:10.1371/journal.pone.0209863)
Supplement: S3 File — (PDF) [file pone.0209863.s003.pdf]

Screen 1 – Project Title Screen

Smart snacking: An online planning tool

(Baseline survey)

A study conducted by the School of Public Health at the University of Sydney

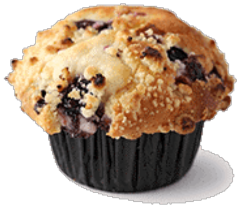 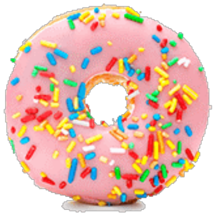 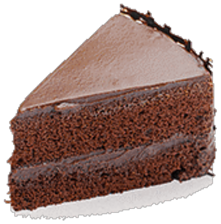 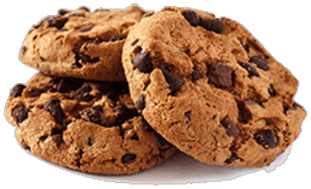

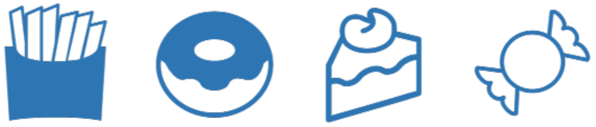

Smart Snacking

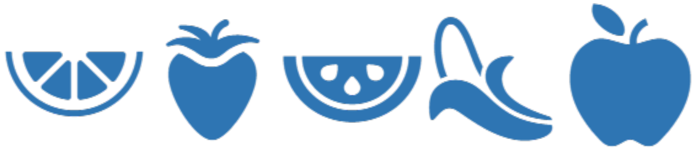

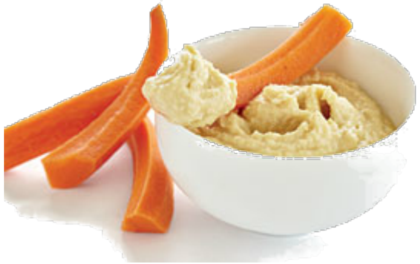 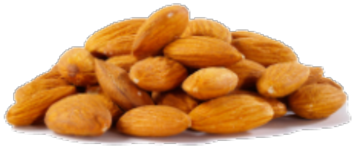 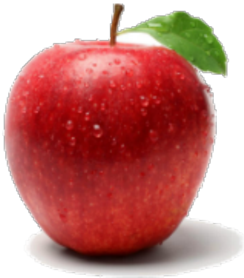

THE UNIVERSITY OF SYDNEY

Screen 1 – Participant information statement

THE UNIVERSITY OF SYDNEY

Study Information Sheet:

Smart snacking: An online planning tool

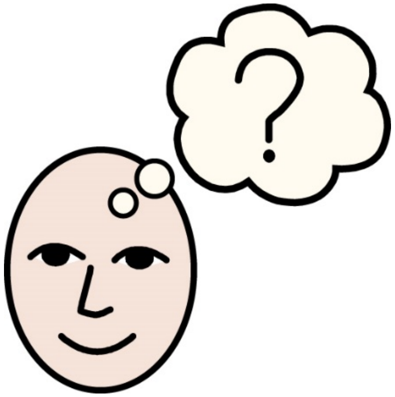

Hello. We are from the School of Public Health at the University of Sydney. Our names are

- Julie Ayre
- Dr Carissa Bonner
- Prof Kirsten McCaffery

We are doing a research study to find out more about tools to help people eat healthy snacks.

Snacks are important because they keep us going until the next meal. Sometimes though, we eat too many or choose snacks that are unhealthy. This can make us gain weight.

While many of us want to change the way we snack, this can be very hard to do. Often we make plans but have trouble sticking to them over long periods of time.

This study will look at online tools that help people stick to their plans.

We are asking you to be in our study because we are looking for people aged 30 years or more, who read and speak adequate English, and who would like to change the way they snack.

You can decide if you want to take part in the study or not. You don't have to - it's up to you.

This sheet tells you what we will ask you to do if you decide to take part in the study. Please read it carefully so that you can make up your mind about whether you want to take part.

You may stop completing the online survey at any point if you do not wish to continue, and we will not use your answers. You do not have to give a reason for not taking part. Once you have submitted your survey anonymously, your responses cannot be withdrawn.

If you have any questions, you can ask us or your family or someone else who looks after you. If you want to, you can call us any time on (02) 9351 7789.

What will happen if I say that I want to be in the study?

If you decide that you want to be in our study, we will ask you to do these things:

- Complete questions online about your demographics (for example, gender, age), the kinds of foods you eat and how you feel about your snacking behaviour
- Use the online planning tool to create a 'smart snacking' plan. You will receive a reminder message after 1 week.
- Try to follow the plan for one month, then complete some online questions about your snacking behaviour and your plan.

You can choose which questions you want to answer. If you don't want to give an answer, that's ok. You can stop answering questions at any time if you don't want to anymore.

This is an online study, so you can take part anywhere with access to the internet (smartphone or computer).

Will anyone else know what I say in the study?

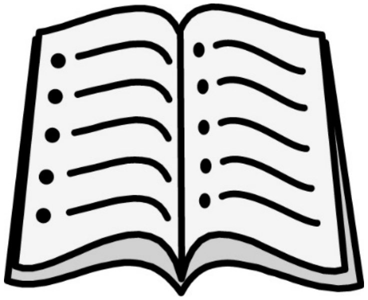

We won't tell anyone else what you say to us, except if you talk about someone hurting you or about you hurting yourself or someone else. Then we might need to tell someone to keep you and other people safe.

All of the information that we have about you from the study will be stored in a safe place and we will look after it very carefully. We will write a report about the study and show it to other people but we won't say your name in the report and no one will know that you were in the study, unless you tell us that it's ok for us to say your name.

How long will the study take?

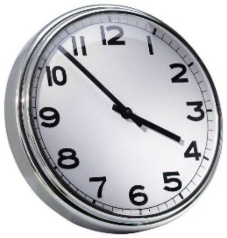

The first part of the study will take about 20 minutes to complete.

Over the following month you will be asked to try out your snacking plan.

The second part of the study will be sent to you after one month, and will take about 10 minutes to complete.

Are there any good things about being in the study?

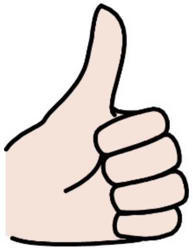

This study may help you think more about the way that you snack. This is the first step to changing your eating patterns. You may also find the tool useful for making these changes.

Are there any bad things about being in the study?

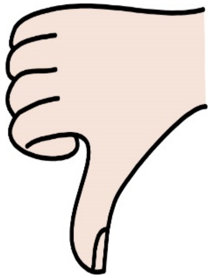

This study will take up some of your time, but we don't think it will be bad for you or cost you anything.

Will you tell me what you learnt in the study at the end?

Yes, we will if you want us to. There is a question on the next page that asks you if you want us to tell you what we learnt in the study. If you select Yes, when we finish the study we will tell you what we learnt.

What if I am not happy with the study or the people doing the study?

The ethical aspects of this study have been approved by the HREC of the University of Sydney [Project Number 2017/662].

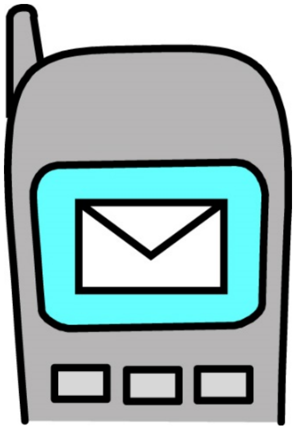

If you are not happy with how we are doing the study or how we treat you, then you or the person who looks after you can:

- **Call** the university on +61 2 8627 8176 or
- Write an **email** to human.ethics@sydney.edu.au

Screen 3 – Participant consent form

I give consent to my participation in the research project

TITLE: **Smart snacking: An online planning tool**

In giving my consent I acknowledge that:

1. The procedures required for the project and the time involved have been explained to me, and any questions I have about the project have been answered to my satisfaction.

2. I have read the Study Information Sheet and have been given the opportunity to discuss the information and my involvement in the project with the researcher/s.

3. I understand that being in this study is completely voluntary – I am not under any obligation to consent.

4. I understand that my involvement is strictly confidential. I understand that any research data gathered from the results of the study may be published however no information about me will be used in any way that is identifiable.

5. *I understand that I can withdraw from the study at any time, without affecting my treatment or my relationship with the researcher(s) or the University of Sydney now or in the future.*

6. I understand that I can stop my participation in this study at any time it I do not wish to continue and we will not use your answers.

7. *By completing the survey you have consented to be part of the study. Once you have submitted your survey anonymously, your responses cannot be withdrawn.*

I give my consent

Yes *No (if click no will not be directed to survey)*

Screen 4 – Baseline information and measures

Before we start please answer the following questions:

Age: [text box to type age]

Gender: [select gender from dropdown menu: Male/Female/Other]

English as first language: [select from dropdown menu: Yes/no], participant selects language from a list.

Highest level of Education: [Select from dropdown menu: Less than high school/high school/Certificate I/II / Certificate III/IV, Diploma, Bachelor degree or equivalent, Masters or Doctoral degree or equivalent]

Height: [participant selects unit of measurement and enters value into textbox]

Weight: [participant selects unit of measurement and enters value into textbox]

Smart snacking

Snacks are important because they keep us going until the next meal. Sometimes though, we eat too many, or choose snacks that are unhealthy.

Even though each snack is usually small, over time the snacks add up. This can make us gain weight.

Smart snacking means choosing nutritious, healthy snacks that give you energy until the next meal.

Which snacks are healthy?

Healthy snacks are low in kilojoules, fat, salt and sugars. These include fresh fruit, vegetables with dip, small amounts of dried fruit or nuts, yoghurt, coffee made with low fat milk, raisin toast, rice crackers and corn thins.

What are unhealthy snacks?

Unhealthy snacks are high in kilojoules, fat, salt and sugars. These include biscuits, cheese crackers, cakes, muffins, pastries, chocolate, lollies, potato chips, hot chips, French fries, some muesli bars and large coffees made with full cream milk.

Next

What are your snacking habits?

Before we get started on smart snacking, we'd like to know a little more about your snacking habits over the last month. For each question please answer by selecting a radio button.

Snacking habits

	Strongly disagree						Strongly agree
1. I eat unhealthy snacks frequently (all the time)	O	O	O	O	O	O	O
2. I eat unhealthy snacks automatically (without thinking)	O	O	O	O	O	O	O
3. I eat unhealthy snacks without having to consciously remember (When I eat unhealthy snacks I have not made an active decision to eat them)	O	O	O	O	O	O	O
4. I feel weird if I do not eat unhealthy snacks	O	O	O	O	O	O	O
5. I eat unhealthy snacks without thinking	O	O	O	O	O	O	O
6. It would require effort not to eat unhealthy snacks	O	O	O	O	O	O	O
7. Unhealthy snacks belong to (are part of) my routine (what I usually do)	O	O	O	O	O	O	O
8. I start eating unhealthy snacks before I realise I'm doing it	O	O	O	O	O	O	O
9. I would find it hard not to eat unhealthy snacks	O	O	O	O	O	O	O
10. I don't need to think about unhealthy snacks	O	O	O	O	O	O	O
11. Unhealthy snacking is typical (normal) for me	O	O	O	O	O	O	O
12. I've been eating unhealthy snacks for a long time	O	O	O	O	O	O	O

Snacks in the last week

Think about your snacking habits in the last week. To what extent have your snacks this week been (how true is it that your snacks this week were):

	Not at all						Very much
1. healthy (e.g. apple, banana, dried fruit)	O	O	O	O	O	O	O
2. unhealthy (e.g. chocolate, crisps, cake)	O	O	O	O	O	O	O

Yesterday's snacks

Which snacks did you eat yesterday? Do not include food eaten during breakfast, lunch or dinner.

- O Hot chips, potato gems or French fries
- O crisps or corn chips, crackers with cheese
- O Muffins, cake or doughnuts
- O biscuits
- O Pretzels
- O Pies, pasties or sausage rolls
- O Muesli bars, fruit bars, breakfast cereal bars
- O Chocolate
- O Lollies
- O Ice cream or ice blocks
- O Coffee with full cream milk
- O Coffee with skim milk
- O tea
- O Vita weat or Ryvita
- O Raisin toast
- O Apple or pear
- O Banana, mango
- O Orange or grapefruit
- O Kiwi fruit, mandarins
- O Yoghurt
- O Cherries, peaches or plums
- O Grapes or berries
- O Watermelon, melon
- O Carrot, cucumber or capsicum
- O Other fruit
- O Dip (e.g. hommos), cottage cheese or peanut butter
- O Nuts
- O Popcorn, rice crackers or corn thins
- O yoghurt
- O Other

Snack diary

1. How often do you usually eat oven baked potato gems/chips/hashbrowns, hot chips/French fries, wedges or fried potatoes?

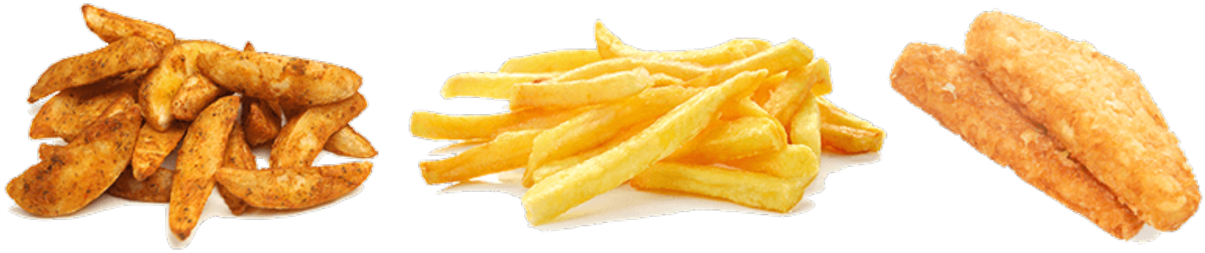

○ each week
○ each day
○ each month
○ I don't eat this

In total, how many serves of potato gems/chips/hashbrowns, hot chips/French fries, wedges or fried potatoes do you usually eat in the timeframe selected above?

1 serve =
12 fried hot chips
1 cup (60g) potato gems/hashbrowns, or wedges

[slider for answer]

2. How often do you usually eat savoury snacks such as crisps, pretzels or plain/flavoured crackers?

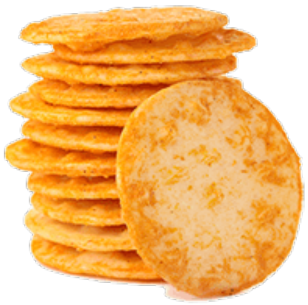 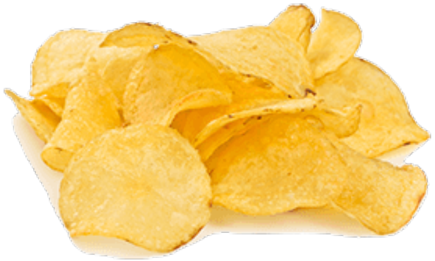 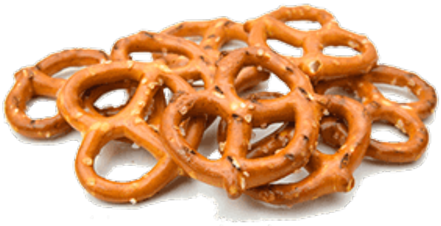

○ each week
○ each day
○ each month
○ I don’t eat this

In total, how many serves of savoury snacks such as crisps, pretzels or plain/flavoured crackers do you usually eat in the timeframe selected above?

1 serve =
½ snack size packet of crisps
1 handful (30g) of salty crackers or pretzels

[slider for answer]

3. How often do you usually have sweet biscuits/cakes/ buns/ muffins/ doughnuts? Include both home-made and bought.

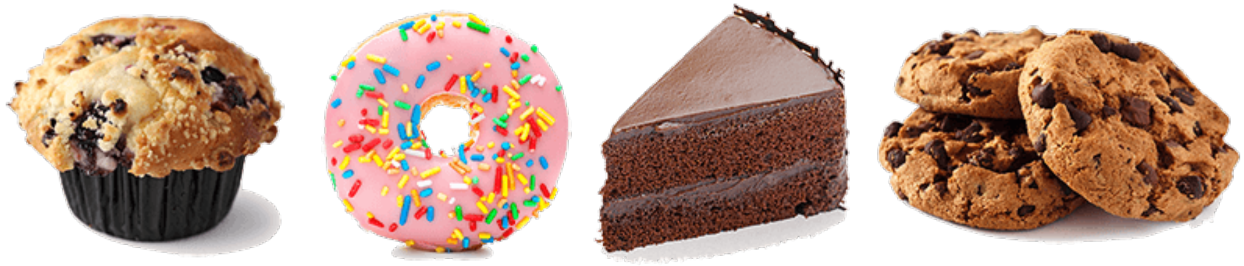

○ each week
○ each day
○ each month
○ I don’t eat this

In total, how many serves of sweet biscuits/cakes/buns/muffins/doughnuts do you usually eat in the timeframe selected above?

1 serve =
2-3 (35g) sweet biscuits
1 doughnut
1 slice (40g) of plain cake or sweet bun
1 small muffin

[slider for answer]

4. How often do you usually eat savoury pastries?

This includes pies, pasties, sausage rolls, Kransky Dogs and frankfurters wrapped in pastry.

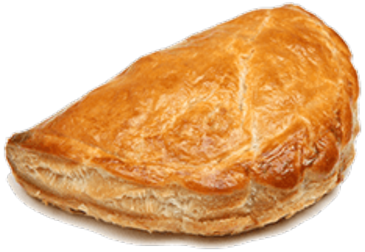 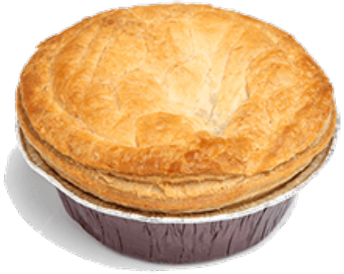 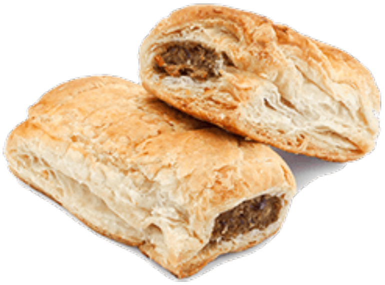

○ each week
○ each day
○ each month
○ I don't eat this

In total, how many serves of pies or savoury pastries do you usually eat in the timeframe selected above?

1 serve =
1/4 (60g) commerical meat pies or pastie
1 party size pie or sausage roll

[slider for answer]

5. How often do you usually eat snack type bars?

This includes muesli bars, fruit bars and breakfast cereal bars.

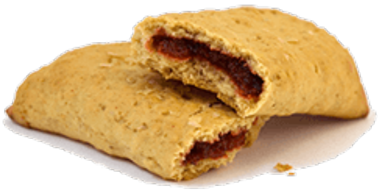 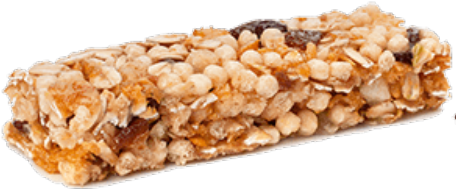 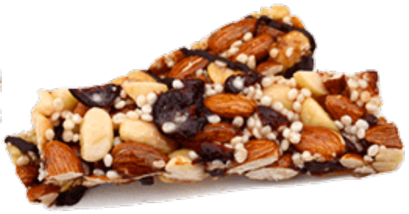

○ each week
○ each day
○ each month
○ I don't eat this

In total, how many snack type bars do you usually eat in the timeframe selected above? This includes muesli bars, fruit bars and breakfast cereal bars.

[slider for answer]

6. How often do you usually have chocolate or lollies? *This question is required.

Include all types of chocolate and both hard and soft lollies.

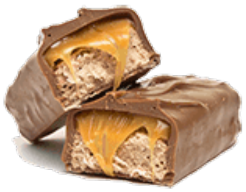 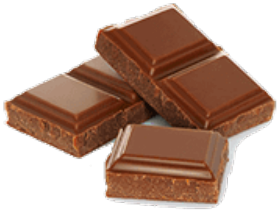 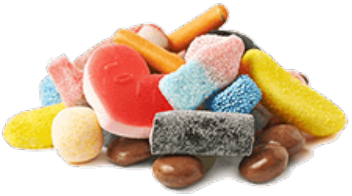 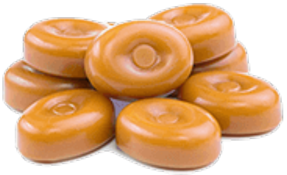

○ each week
○ each day
○ each month
○ I don’t eat this

In total, how many serves of chocolate or lollies do you usually eat in the timeframe selected above?

1 serve =
½ chocolate bar
4 pieces of chocolate (25g)
5-6 (40g) lollies

[slider for answer]

7. How often do you usually have ice-cream or ice-blocks?

This includes ice-blocks, ice-cream in a bowl or ice-creams on a stick.

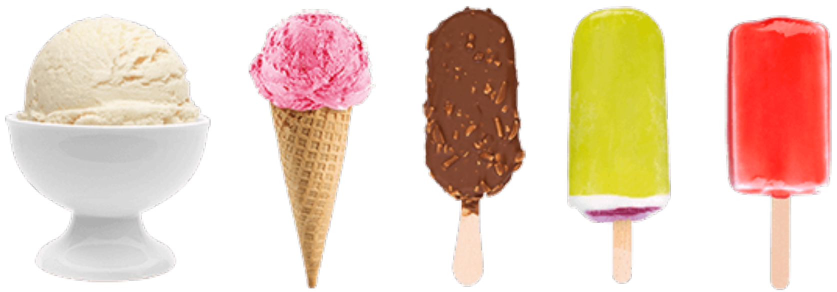

○ each week
○ each day

○ each month
○ I don't eat this

In total, how many serves of ice-cream or ice-blocks do you usually eat in the timeframe selected above? *This question is required.

1 serve =
2 scoops (60g) ice-cream
1 stick ice-cream or ice-block

[slider for answer]

What do you think about snacking?

**Over the next month:**	Strongly disagree						Strongly agree
7. I want to eat fewer unhealthy snacks	○	○	○	○	○	○	○
8. I plan to eat fewer unhealthy snacks	○	○	○	○	○	○	○
9. I intend (plan) to eat fewer unhealthy snacks	○	○	○	○	○	○	○

What snacking strategies do you already have?

**I already have clear plans about…**	Strongly disagree						Strongly agree
1. How I will change my unhealthy snacking habits	○	○	○	○	○	○	○
2. When I will change my unhealthy snacking habits	○	○	○	○	○	○	○
3. When I need to watch out so that I keep choosing healthy snacks	○	○	○	○	○	○	○
4. What to do in situations that make it hard to avoid unhealthy snacks (times when it is hard not to eat unhealthy snacks)	○	○	○	○	○	○	○
5. How to get back on track when I have eaten unhealthy snacks	○	○	○	○	○	○	○

How do you feel about changing your unhealthy snacking?

I am sure that…	Strongly disagree						Strongly agree
1. I can avoid eating unhealthy snacks for the next month	○	○	○	○	○	○	○
**I am certain that I can avoid eating unhealthy snacks even if…**							
2. Friends or family are eating unhealthy snacks	○	○	○	○	○	○	○
3. I am bored	○	○	○	○	○	○	○
4. I am craving an unhealthy snack	○	○	○	○	○	○	○

Nobody is perfect. Sometimes we have trouble sticking to our plans. Imagine you have started eating unhealthy snacks again. How confident are you about changing this habit?

I am certain I could go back to eating healthy snacks…							
5. Even after I ate 1 unhealthy snack	○	○	○	○	○	○	○
6. Even after a few days of eating unhealthy snacks	○	○	○	○	○	○	○
7. Even after a week of eating unhealthy snacks	○	○	○	○	○	○	○

Please read the nutritional panel below to answer the following questions. The panel is information on the back of a container or ice cream.

**Nutrition Facts**	
Serving Size	1/2 cup
Servings per container	4
Amount per serving	
Calories 250	Fat Cal 120
	%DV
**Total Fat** 13g	20%
Sat Fat 9g	40%
**Cholesterol** 28mg	12%
**Sodium** 55mg	2%
**Total Carbohydrate** 30g	12%
Dietary Fiber 2g	
Sugars 23g	
**Protein** 4g	8%

* Percent Daily Values (DV) are based on a 2,000 calorie diet. Your daily values may be higher or lower depending on your calorie needs.

Ingredients: Cream, Skim Milk, Liquid Sugar, Water, Egg Yolks, Brown Sugar, Milkfat, Peanut Oil, Sugar, Butter, Salt, Carrageenan, Vanilla Extract.

1. If you eat the entire container, how many calories will you eat?

2. If you are allowed to eat 60 grams of carbohydrates as a snack, how much ice cream could you have?

3. Your doctor advises you to reduce the amount of saturated fat in your diet. You usually have 42g of saturated fat each day, which includes one serving of ice cream. If you stop eating ice cream, how many grams of saturated fat would you be consuming each day?

4. If you usually eat 2,500 calories in a day, what percentage of your daily value of calories will you be eating if you eat one serving?

Pretend that you are allergic to the following substances: penicillin, peanuts, latex gloves, and bee stings.

5. Is it safe for you to eat this ice cream?
6. *[Ask only if patient responds 'no' to question 5]*: Why not?

Great! It looks like you're ready to **snack smarter**! The next step is to come up with a plan.

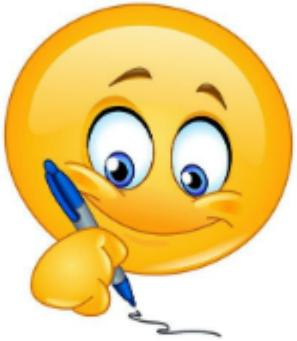

Let's get started!

The next section depends on intervention condition.

Screen 4a – Volitional help sheet condition

Smart snacking

Step 1: Snack moments

Sometimes we snack because we are hungry, but there are lots of other reasons too.

Think about your snacks in the **last week**. Below is a list of '**snack moments**.' These are times when people tend to choose unhealthy snacks or eat too much.

Choose **3** snack moments from the list that happened to you the **most often** in the last week.

I often eat unhealthy snacks when…

The snack is in front of me	I have a craving	I am bored	I am tired	I start with one piece but then keep eating	I am in front of a TV or computer
Someone offers me the snack	People around me are eating	I am happy	I am drinking alcohol	I am busy or stressed	I am about to go to bed
I am drinking tea or coffee	I am sad	I want to reward myself	it is part of a celebration or special event	it is my day off	I have arrived home

Next

Step 2: The 'key' snack moment

Below are your top 3 snack moments.

Some snack moments will be more important than others. Choose the **1** that you would be **happiest** to change.

I really want to change my habit of snacking when…

I am bored

I am sitting in front of a TV or computer

people around me are eating

Next

Step 3: Make a plan

Great! Your key snack moment is:
Eating unhealthy snacks when **I am bored [example text]**.

The last step is to come up with a plan! **Choose the solution that you think will work best for you**. Drag it into the space on the right

**I will go outside for a walk**	**I will listen to music**	**I will chat to someone for 5 minutes**
**I will drink tea**	**I will do a chore or task**	**I will eat a smaller amount**
**I will drink a large glass of water**	**I will eat a piece of fruit**	**I will take the food out of the packet and put it on a plate**
	**I will eat fresh vegetables and dip**	

If I want a snack because I am bored, I will…

Next

Step 4: Your plan is almost ready!

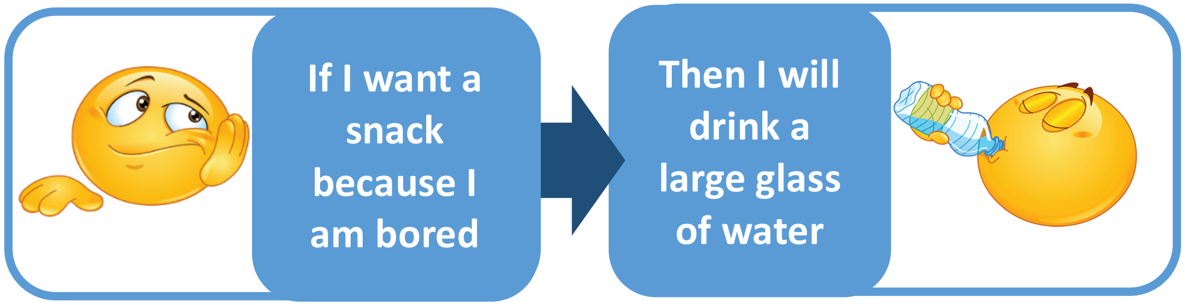

Imagine how your plan might feel. **When do you feel bored?**

Sometimes we want unhealthy snacks when we :

- waiting for a friend to arrive
- on a long train or bus trip
- watching TV
- doing long repetitive tasks

If this happens, and you want an unhealthy snack, do you think you could drink a large glass of water instead?

How hard do you think it will be to do this plan for the next month?

Very easy | Very hard

[If score for previous question is <7/10]

Your score shows that this plan may be hard for you.

Let's make a plan that is a bit easier!

I will go outside for a walk	I will listen to music	I will chat to someone for 5 minutes
I will drink tea	I will do a chore or task	I will eat a smaller amount
I will drink a large glass of water	I will eat a piece of fruit	I will take the food out of the packet and put it on a plate
	I will eat fresh vegetables and dip	

If I want a snack because I am bored, I will…

[If score for previous question is ≥7/10]

Step 4: Your plan is almost ready!

Your smart snacking plan

Well done! Try to remember this plan for the **next month**. Say it **3** times to yourself. You can also make a copy of your plan at the end of this survey.

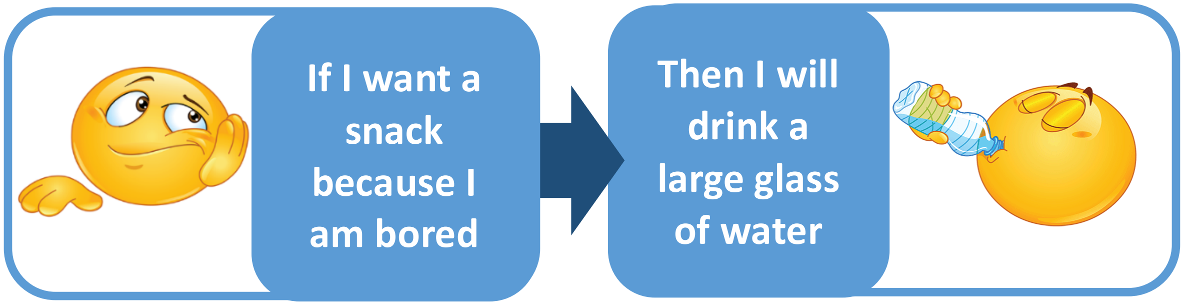

Take a screenshot

Next

Next

Screen 4b – Detailed planning condition (active control)

Smart snacking

Your healthy snacking plan

We want you to plan how you will change your unhealthy snacking behaviour each day because forming plans has been shown to improve snacking habits.

You are free to choose how you do this but we want you to formulate your plans in as much detail as possible.

Please pay attention to the **situations** in which you will implement (carry out) these plans. Focus on situations when you are not hungry but find yourself snacking.

Please enter your plan below
[Text box – e.g. When I am bored and hungry I will remember to drink a large glass of water first.]

How hard do you think it will be to do this plan for the next month?

Very easy Very hard

Next

Your healthy snacking plan

Well done! Try to remember this plan for the **next month**. Say it a few times to yourself. You may also like to write the plan down or take a screenshot. You can also download this plan as a PDF at the end of the survey.

[Example: When I am bored and hungry I will remember to drink a large glass of water first.]

Take a screenshot

Next

Screen 4c – Helpful hints sheet (passive control)

Smart snacking

Read the 'healthy snacks' fact sheet below.

Healthy snacks

Eating the right balance of healthy foods can help you live well. Snacks can help you meet your daily nutrition needs, but it’s important to make healthy choices and watch your portions to manage your weight.

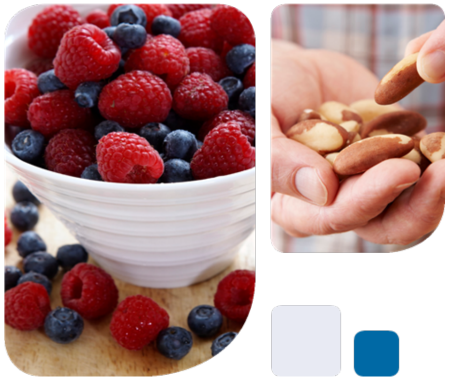

The snacks below are good choices. Try these in the recommended portions.

Fruit

» Fresh fruit: one apple/pear/orange/ large peach/large nectarine/small banana; three apricots/mandarins; two kiwifruit/plums; one cup of grapes/ cherries/melon
» Canned fruit in natural juice (drained): one cup of fruit salad/apricots/peaches
» Dried fruit: one tablespoon of sultanas; six prunes; four whole dried apricots

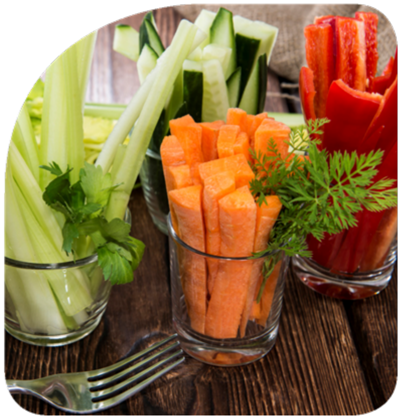

Vegetables

» ½ cup no-added-salt baked beans
» One steamed cob of corn
» Small can (125g) corn kernels
» Vegetable sticks: celery, carrot, capsicum, cucumber or snow peas with 1 slice (20g) of low-fat cheese or one tablespoon of hummus, tomato salsa or tzatziki yoghurt dip
» Celery boats with two tablespoons of low-fat cream cheese or no-added-salt peanut butter
» A handful of cherry tomatoes

Dairy and alternatives

» 100g of low-fat flavoured yoghurt or 200g of diet/natural/plain varieties. Flavour natural yoghurt with passionfruit, fresh/frozen berries, one teaspoon of honey, nuts or seeds
» 250ml of low-fat plain milk/soy milk with added calcium
» Medium cappuccino/café latte/flat white coffee with low-fat milk/soy milk

Breads and cereals

» One slice of dense grainy/seeded bread (plain or toasted) with a thin spread of margarine or avocado or low-fat ricotta or cottage cheese or hummus or chutney, with sliced tomato and sprouts
» One thin slice of raisin/wholegrain fruit bread, fresh or toasted, with a thin spread of margarine or no-added-salt peanut butter/natural nut spread or 100% fruit jam
» Half a wholegrain English muffin grilled with one slice of low-fat cheese and a sliced tomato
» One small wholemeal pita pocket or wrap with grated carrot and grated low-fat cheese
» Four rice/corn thins spread with a ¼ of an avocado and topped with sliced cucumber and tinned salmon
» Ten plain wholegrain rice crackers with low-fat cream cheese or hummus or salsa
» Two wholegrain crispbread with cottage cheese or avocado and tomato

Nuts and seeds

» 30 grams (a small handful) of plain, raw unsalted nuts: mixed, macadamias, walnuts, almonds, cashews, brazil, hazelnuts, pecans, pistachios
» 30 grams (a small handful) of pumpkin or sunflower seeds

Lean meat and alternatives

» A mini can of tuna/salmon/sardines served in half a capsicum or a lettuce cup
» A hard-boiled egg

Grains

» One cup of air-popped popcorn served plain, or sprinkled with cinnamon/ paprika/chilli

Muesli and nut bars

Muesli and nut bars are a ‘sometimes’ snack as they can be high in kilojoules, added fat and sugar. Check the nutrition information panel to choose higher fibre/ healthier options and eat these only occasionally.

Homemade snacks

Homemade snacks can be a healthy choice but they can still be high in kilojoules. Eat small portions of these only occasionally.
» Cakes, biscuits, muffins and slices baked at home can be made healthier by using olive, canola, rice bran, grapeseed or sunflower margarines or oils; less sugar, wholemeal flour, oats, nuts, seeds and fruit.

When shopping, try to limit buying foods that are high in saturated fat, sugar, salt (sodium), such as:

- sweet biscuits and some crackers
- cakes, muffins and slices,
- pastries
- chocolates and confectionary
- potato crisps, corn chips and other salty snacks
- processed and pre-packaged snack foods
- fast foods and high fat takeaways

Drinks

It’s important to stay hydrated throughout the day. Water is the best drink, but some other alternatives include:
» mineral or soda water flavoured with sliced lemon/lime/frozen berries/ cucumber/fresh mint
» black/oolong/green/herbal tea
» coffee or decaffeinated coffee with a dash of skim or low-fat milk.
As an occasional substitute, diet cordial or diet soft drink may add variety without extra sugar or kilojoules.

What is your plan? How hard do you think it will be to do this plan for the next month?

Very easy Very hard

Next

Screen 5 – Tool evaluation

Great work! Now you have a plan to try out for the next month.

We would like to ask a few more questions before you finish.

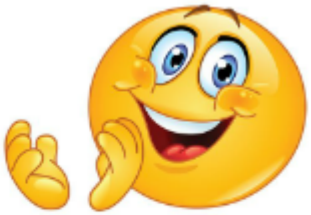

How hard was the tool to use?

Not at all hard	A little hard	Somewhat hard	Very hard	Extremely hard
○	○	○	○	○

Screen 6 – Health literacy measures

1. How confident are you filling out medical forms by yourself?

Not at all	A little bit	Somewhat	Quite a bit	Extremely
○	○	○	○	○

On a scale from very difficult to very easy, how easy would you say it is to:

	Very difficult	Fairly difficult	Fairly easy	Very easy	Don't know
1. Judge when you may need to get a second opinion from another doctor?	○	○	○	○	○
2. Use information the doctor gives you to make decisions about your illness?	○	○	○	○	○
3. Find information on how to manage mental health problems like stress or depression?	○	○	○	○	○
4. Judge if the information on health risks in the media is reliable?	○	○	○	○	○
5. Find out about activities that are good for your mental well-being?	○	○	○	○	○
6. Understand information in the media on how to get healthier?	○	○	○	○	○

That's it! Good luck with your smart snacking plan!

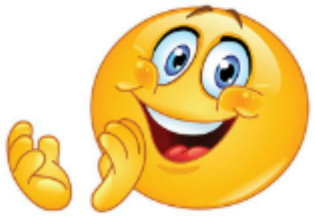

Before you leave, would you like to print or download a copy of your plan?

Print or download plan
to download a PDF select 'print as PDF' when you select the printer.

[space below will show a copy of the plan OR a copy of the tips sheet]

Next

Before we finish, it is very important that you have a copy of your plan!

You can write it down, print it out or save it as a PDF or screenshot.

Press the 'back' button if you need to make a copy. If you have a copy, click 'yes' below.

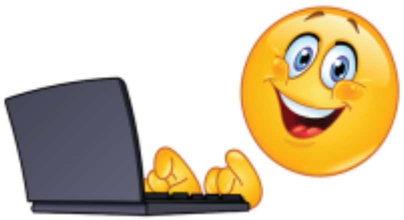

Yes, I have a copy of my plan!

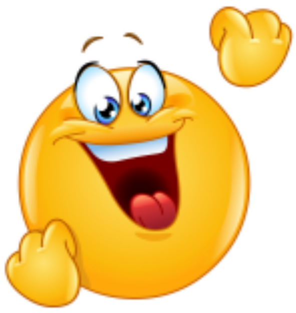

That's it! Good luck with your smart snacking plan!

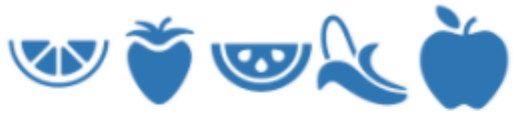

Thank you for completing this survey.

We will check in with you in **one month** to see how your plan is going.

Screen 1 – Project Title Screen

Smart snacking: An online planning tool
(Follow-up survey)

A study conducted by the School of Public Health at the University of Sydney

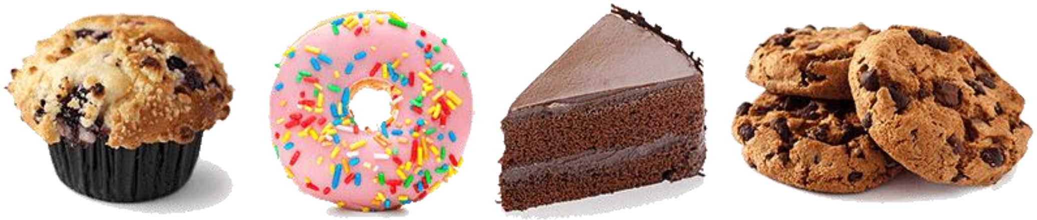

Smart Snacking

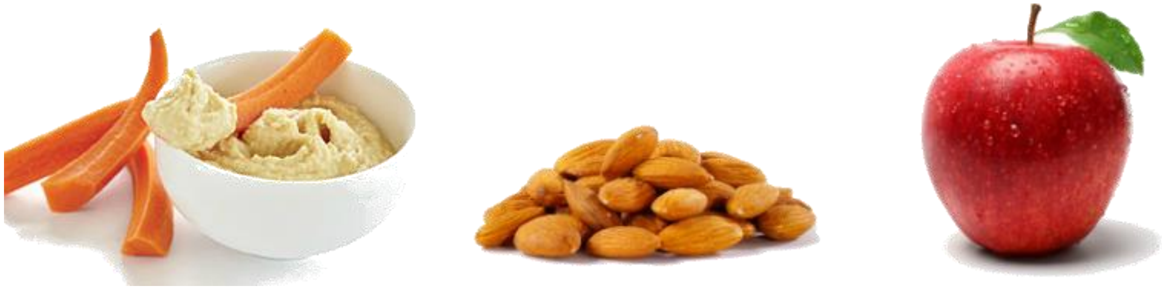

THE UNIVERSITY OF SYDNEY

Smart snacking

Snacks are important because they keep us going until the next meal. Sometimes though, we eat too many, or choose snacks that are unhealthy.

Even though each snack is usually small, over time the snacks add up. This can make us gain weight.

Smart snacking means choosing nutritious, healthy snacks that give you energy until the next meal.

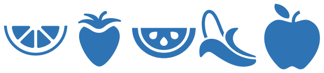

Which snacks are healthy?

Healthy snacks are low in kilojoules, fat, salt and sugars. These include fresh fruit, vegetables with dip, small amounts of dried fruit or nuts, yoghurt, coffee made with low fat milk, raisin toast, rice crackers and corn thins.

What are unhealthy snacks?

Unhealthy snacks are high in kilojoules, fat, salt and sugars. These include biscuits, cheese crackers, cakes, muffins, pastries, chocolate, lollies, potato chips, hot chips, French fries, some muesli bars and large coffees made with full cream milk.

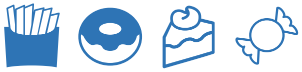

Next

What are your snacking habits?

Before we get started on smart snacking, we'd like to know a little more about your snacking habits over the last month. For each question please answer by selecting a radio button.

Snacking habits

	Strongly disagree						Strongly agree
1. I eat unhealthy snacks frequently (all the time)	O	O	O	O	O	O	O
2. I eat unhealthy snacks automatically (without thinking)	O	O	O	O	O	O	O
3. I eat unhealthy snacks without having to consciously remember (When I eat unhealthy snacks I have not made an active decision to eat them)	O	O	O	O	O	O	O
4. I feel weird if I do not eat unhealthy snacks	O	O	O	O	O	O	O
5. I eat unhealthy snacks without thinking	O	O	O	O	O	O	O
6. It would require effort not to eat unhealthy snacks	O	O	O	O	O	O	O
7. Unhealthy snacks belong to (are part of) my routine (what I usually do)	O	O	O	O	O	O	O
8. I start eating unhealthy snacks before I realise I'm doing it	O	O	O	O	O	O	O
9. I would find it hard not to eat unhealthy snacks	O	O	O	O	O	O	O
10. I don't need to think about unhealthy snacks	O	O	O	O	O	O	O
11. Unhealthy snacking is typical (normal) for me	O	O	O	O	O	O	O
12. I've been eating unhealthy snacks for a long time	O	O	O	O	O	O	O

Snacks in the last week

Think about your snacking habits in the last week. To what extent have you eaten (how true is it that you have eaten):

	Not at all						Very much
1. healthy snacks? (e.g. apple, banana, dried fruit)	O	O	O	O	O	O	O
2. unhealthy snacks? (e.g. chocolate, crisps, cake)	O	O	O	O	O	O	O

Yesterday's snacks

Which snacks did you eat yesterday? Do not include food eaten during breakfast, lunch or dinner.

- O Hot chips, potato gems or French fries
- O crisps or corn chips, crackers with cheese
- O Muffins, cake or doughnuts
- O biscuits
- O Pretzels
- O Pies, pasties or sausage rolls
- O Muesli bars, fruit bars, breakfast cereal bars
- O Chocolate
- O Lollies
- O Ice cream or ice blocks
- O Coffee with full cream milk
- O Coffee with skim milk
- O tea
- O Vita weat or Ryvita
- O Raisin toast
- O Apple or pear
- O Banana, mango
- O Orange or grapefruit
- O Kiwi fruit, mandarins
- O Yoghurt
- O Cherries, peaches or plums
- O Grapes or berries
- O Watermelon, melon
- O Carrot, cucumber or capsicum
- O Other fruit
- O Dip (e.g. hommos), cottage cheese or peanut butter
- O Nuts
- O Popcorn, rice crackers or corn thins
- O yoghurt
- O Other

Snack diary

1. How often do you usually eat oven baked potato gems/chips/hashbrowns, hot chips/French fries, wedges or fried potatoes?

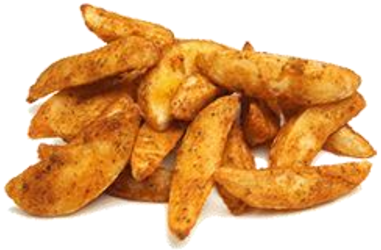
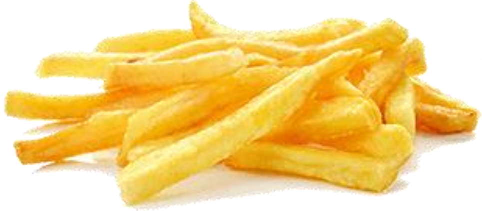
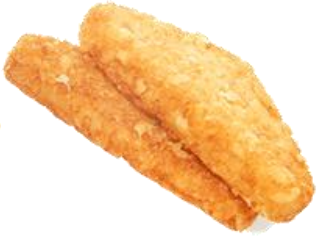

○ each week
○ each day
○ each month
○ I don't eat this

In total, how many serves of potato gems/chips/hashbrowns, hot chips/French fries, wedges or fried potatoes do you usually eat in the timeframe selected above?

1 serve =
12 fried hot chips
1 cup (60g) potato gems/hashbrowns, or wedges

[slider for answer]

2. How often do you usually eat savoury snacks such as crisps, pretzels or plain/flavoured crackers?

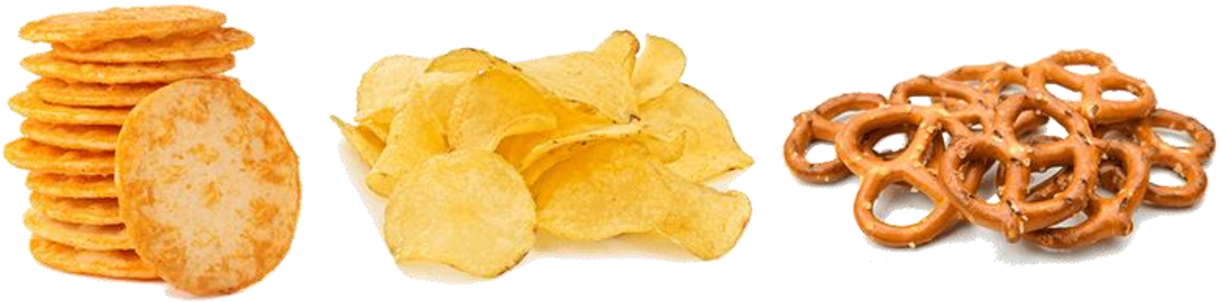

○ each week
○ each day
○ each month
○ I don’t eat this

In total, how many serves of savoury snacks such as crisps, pretzels or plain/flavoured crackers do you usually eat in the timeframe selected above?

1 serve =
½ snack size packet of crisps
1 handful (30g) of salty crackers or pretzels

[slider for answer]

3. How often do you usually have sweet biscuits/cakes/ buns/ muffins/ doughnuts? Include both home-made and bought.

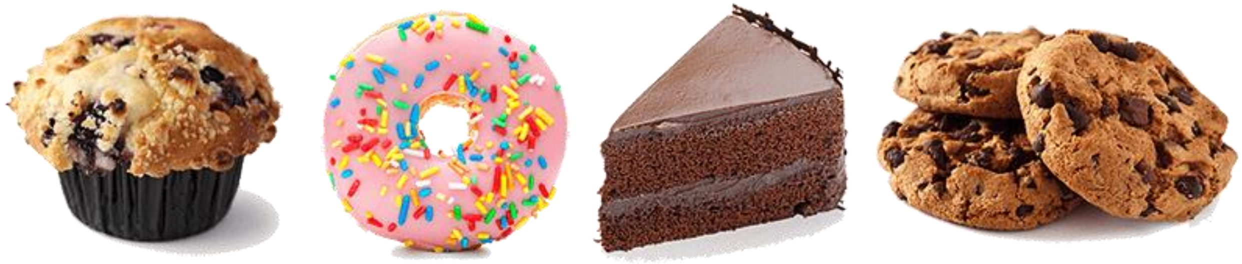

○ each week
○ each day
○ each month
○ I don’t eat this

In total, how many serves of sweet biscuits/cakes/buns/muffins/doughnuts do you usually eat in the timeframe selected above?

1 serve =
2-3 (35g) sweet biscuits
1 doughnut
1 slice (40g) of plain cake or sweet bun
1 small muffin

[slider for answer]

4. How often do you usually eat savoury pastries?

This includes pies, pasties, sausage rolls, Kransky Dogs and frankfurters wrapped in pastry.

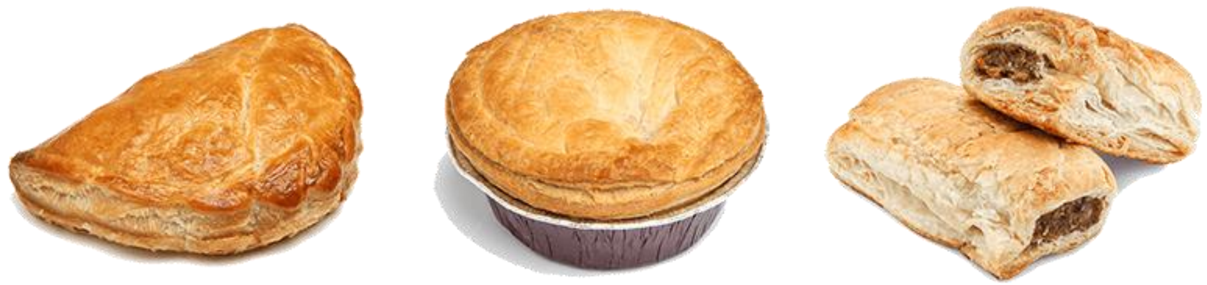

- ○ each week
- ○ each day
- ○ each month
- ○ I don’t eat this

In total, how many serves of pies or savoury pastries do you usually eat in the timeframe selected above?

1 serve =
1/4 (60g) commerical meat pies or pastie
1 party size pie or sausage roll

[slider for answer]

5. How often do you usually eat snack type bars?

This includes muesli bars, fruit bars and breakfast cereal bars.

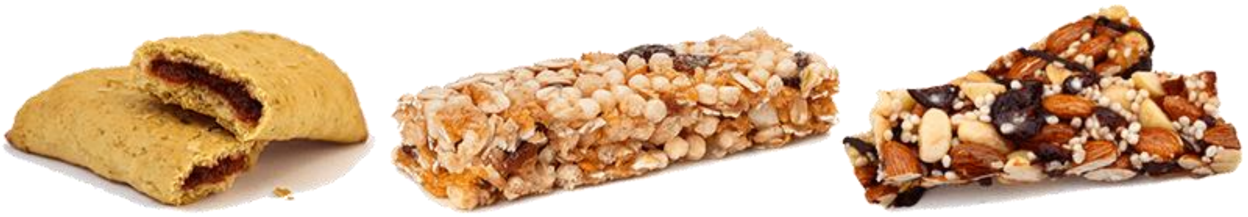

○ each week
○ each day
○ each month
○ I don’t eat this

In total, how many snack type bars do you usually eat in the timeframe selected above? This includes muesli bars, fruit bars and breakfast cereal bars.

[slider for answer]

6. How often do you usually have chocolate or lollies? *This question is required.

Include all types of chocolate and both hard and soft lollies.

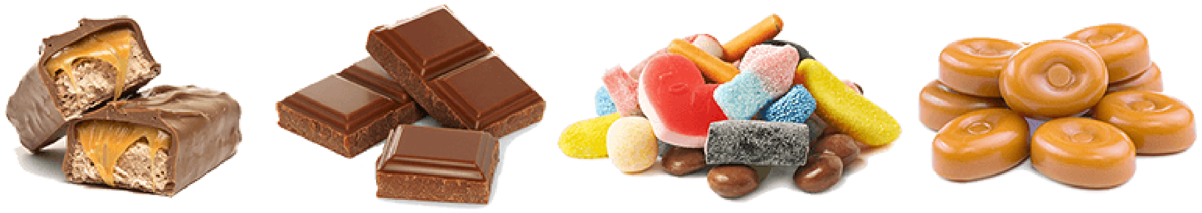

○ each week

○ each day
○ each month
○ I don't eat this

In total, how many serves of chocolate or lollies do you usually eat in the timeframe selected above?

1 serve =
½ chocolate bar
4 pieces of chocolate (25g)
5-6 (40g) lollies

[slider for answer]

7. How often do you usually have ice-cream or ice-blocks?

This includes ice-blocks, ice-cream in a bowl or ice-creams on a stick.

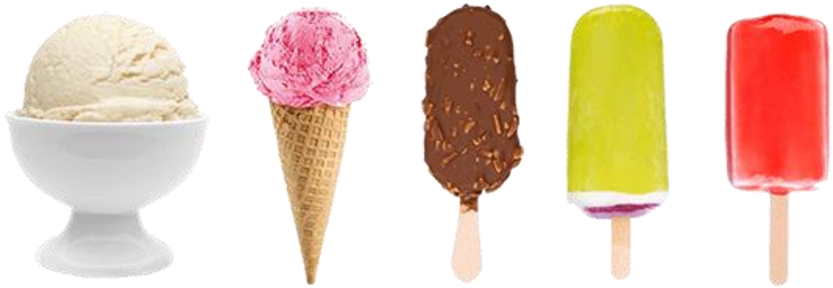

○ each week
○ each day
○ each month
○ I don't eat this

In total, how many serves of ice-cream or ice-blocks do you usually eat in the timeframe selected above? *This question is required.

1 serve =
2 scoops (60g) ice-cream
1 stick ice-cream or ice-block

[slider for answer]

What do you think about snacking?

**Over the next month:**	Strongly disagree						Strongly agree
7. I want to eat fewer unhealthy snacks	○	○	○	○	○	○	○
8. I plan to eat fewer unhealthy snacks	○	○	○	○	○	○	○
9. I intend (plan) to eat fewer unhealthy snacks	○	○	○	○	○	○	○

What snacking strategies do you already have?

**I have clear plans about…**	Strongly disagree	Strongly agree

1. How I will change my unhealthy snacking habits	○	○	○	○	○	○	○
2. When I will change my unhealthy snacking habits	○	○	○	○	○	○	○
3. When I need to watch out so that I keep choosing healthy snacks	○	○	○	○	○	○	○
4. What to do in situations that make it hard to avoid unhealthy snacks (times when it is hard not to eat unhealthy snacks)	○	○	○	○	○	○	○
5. How to get back on track when I have eaten unhealthy snacks	○	○	○	○	○	○	○

How do you feel about changing your unhealthy snacking?

**I am sure that…**	Strongly disagree						Strongly agree
1. I can avoid eating unhealthy snacks for the next month	○	○	○	○	○	○	○
**I am certain that I can avoid eating unhealthy snacks even if…**							
2. Friends or family are eating unhealthy snacks	○	○	○	○	○	○	○
3. I am bored	○	○	○	○	○	○	○
4. I am craving an unhealthy snack	○	○	○	○	○	○	○

Nobody is perfect. Sometimes we have trouble sticking to our plans. Imagine you have started eating unhealthy snacks again. How confident are you about changing this habit?

I am certain I could go back to eating healthy snacks…

5. Even after I ate 1 unhealthy snack	O	O	O	O	O	O	O
6. Even after a few days of eating unhealthy snacks	O	O	O	O	O	O	O
7. Even after a week of eating unhealthy snacks	O	O	O	O	O	O	O

How did you go with your snacking plan?

**During the last month…**	Strongly disagree						Strongly agree
I often thought about my plan to reduce the number of (eat less) unhealthy snacks I ate each day	○	○	○	○	○	○	○
I constantly (often) kept count of how many unhealthy snacks I ate to make sure it was not too much	○	○	○	○	○	○	○
I reminded myself to make sure I wasn't having too many unhealthy snacks	○	○	○	○	○	○	○
I tried my best to be consistent with my plan (stick with my plan) to eat less unhealthy snacks	○	○	○	○	○	○	○
I really tried to reduce the number of (eat less) unhealthy snacks I ate each day	○	○	○	○	○	○	○

That is the end of the survey, thank you for your participation.

If you would like more information about the study, please click here.

If you would like to receive a copy of the results of this study, please email the study office at julie.ayre@sydney.edu.au
