# Supplementary material for: Randomized trial of planning tools to reduce unhealthy snacking: Implications for health literacy
Source: PLoS One. 2019 Jan 17;14(1):e0209863. doi: 10.1371/journal.pone.0209863 (PMC6336265; doi:10.1371/journal.pone.0209863)
Supplement: S5 File — (DOCX) [file pone.0209863.s005.docx]

# Summary of trial protocol submitted and approved by the University of Sydney’s Human Research Ethics Committee

## Aims:

The aims of the study are threefold: (1) to investigate the impact of an online planning tool on unhealthy snacking behaviour at one-month follow-up; (2) to compare this effect amongst higher and lower health literacy groups, and; (3) to investigate possible mediating factors or predictors of the effectiveness of the intervention, and how this relates to health literacy.

## Hypotheses:

1. Participants using the volitional help sheet will have higher maintenance self-efficacy scores than participants using the detailed planning intervention, and participants in these groups (averaged) will have higher scores than participants in the passive control group.

2. Participants using the volitional help sheet will have lower unhealthy snacking scores than participants using the detailed planning intervention, and participants in these groups (averaged) will have lower scores than participants in the passive control group.

3. Change in behaviour will be mediated by maintenance self-efficacy

4. The effects of planning tool will be moderated by health literacy. It is hypothesised that the volitional help sheet will be more useful (i.e. reduce unhealthy snacking to a greater extent) for people with lower healthy literacy than the detailed planning intervention

## Research plan including duration of the study and/or timeline:

It is estimated that recruitment and data collection will take three months (including a month interim period between the first and second survey). The study will be conducted via two online surveys (Qualtrics). The first survey will take approximately 20mins and the second approximately 10mins.

Participant characteristics including sex, age range and inclusion/exclusion criteria (if relevant):

A sample of 435 eligible participants will be recruited through SSI, a ‘software-as-a-service’ company. They will recruit potential participants from their existing database until 435 eligible participants are identified based on screening questions to assess eligibility: at least 30 years of age, and sufficiently able to understand health-related information in English. Furthermore, in order to facilitate the involvement of people likely to be of lower health-literacy, half (50%) of all participants will have no university degree. Other than that, potential participants will be recruited to reflect the general population in terms of sex, age, cultural background, employment status, income.

## The intended sample size with a justification, and/or the participant sampling/selection strategy (as relevant to your study):

The intended sample size is approximately 435 participants. Piloting the survey will allow for a more definite sample size to be determined. Eighty-seven participants randomized to each group (totalling 261 participants) will yield ~90% power to detect moderate effect sizes (Cohen’s f = 0.25, corresponding to ANOVA effects and interaction as small as ?p 2 = 0.06) in the primary outcomes (maintenance self-efficacy scores; unhealthy snacking scores) and any secondary analyses, assuming a Bonferroni-adjusted two-sided alpha of 0.017 (allowing for four planned comparisons, and a correlation between the two primary outcome measures of ~ 0.25). Additionally, just under 50% additional cases will be recruited to account for potential missing values or attrition, totalling 435 participants (i.e., 145 per group).

## Details of where the study will be undertaken (location/site/URL):

The online survey will be conducted via the Qualtrics website (www.qualtrics.com). Survey Sampling International (SSI) will send participants specific URLs to the survey. The University of Sydney will not make any direct contact with the participants.

## Details of how data will be collected:

Data will be collected directly from participants in the form of two online surveys. These will be spaced one-month apart. See the procedure below for further details as to data collection:

## Procedure:

### Participant recruitment and informed consent

Qualtrics will host the survey, and SSI will be used for recruitment of participants. Only SSI will have access to participant details (e.g. names and email addresses). Participants will be allocated an ID (provided to SSI) and this will be used to link baseline and follow up survey data. The data stored on Qualtrics’ server will therefore be de-identified and cannot be linked to participant details. SSI will not have access to the survey results as these are stored in the Qualtrics server in the specific the University of Sydney School of Public Health account used for this project. The investigators on this project are the only individuals with access to this account. The Qualtrics servers are protected by high-end firewall systems, secure data centres, and the company does not sell or make available specific information about participants except as required by law. This is outlined in the Qualtrics security statement and the Qualtrics privacy statement. Furthermore, the data that is stored on the Qualtrics server is de-identified (as stated above, participant contact details are only accessible to SSI).

Participants selected for this experiment will be men and women aged 30 years and older. Half of all participants will have no university degree. All participants will be required to be able to read and write in English. No other quotas or restrictions will be placed on participant recruitment. In other words, participants will be representative of the Australian community in terms of age, gender, socioeconomic status etc. The University of Sydney will not obtain any contact details from potential participants.

Initial contact will be made by SSI, who will use their database to approach potential participants who meet the eligibility criteria. Participants listed on their database have already indicated a willingness to participate in online research. If participants agree and are interested in being part of the study, they will be directed to an online Participant Information Statement, Consent Form, and then the surveys themselves.

Participants are free to withdraw from the study at any time. After reading the online Participant Information Statement, potential participants will be able elect to not proceed to the survey. It will be outlined in the Participation Information Statement that there will be no consequences to the individual should they wish to withdraw, and that they are able to withdraw from the survey at any point. In addition, participants who proceed to the survey and then discontinue survey completion part way through will be treated as though consent has been withdrawn, and their responses will not be used.

Participants will not receive any financial or other reward from the University of Sydney. SSI, the company that will be recruiting the participants, have a points-system whereby points are earned for completion of surveys. Specifically, points are based on survey length rather than being offered for particular surveys. These points can be redeemed for various items such as shopping vouchers and airline vouchers. The points represent modest compensation for the time that participants forgo by participating in the study. Participants will complete two surveys, 1 month apart. They will receive points on completion of the first survey and then more points again after completing the second survey. To be able to complete a survey and to collect the points, the respondent needs to quality for a survey, i.e. profile match and pass all quality checks. The value of the points in itself is not competitive with the minimum wage per hour, to avoid encouraging fake responses. This ensures that the completed surveys are quality-assured and less likely to be influenced by ‘incentives-skewed bias.

Participants will read an online Participant Information Statement followed by an online Consent Form. At the bottom of the Consent Form, participants will be asked “Do you consent”. They may click either ‘Yes’ or ‘No’. If participants click ‘Yes’ they will continue to the survey. If participants click ‘No’ they will not be able to continue to the survey. The University will only receive data from respondents that complete the surveys. To do so they have to have clicked ‘Yes’ at the Consent Form. Therefore, completing the survey will itself self as an indicator that they have consented (consistent with the NHMRC National Statement on Ethical Conduct in Human Research [2007]). Furthermore, participants, by virtue of being on the SSI database, have already consented to being involved in online research.

### Baseline (questionnaire and intervention):

Participants will complete a series of baseline questionnaires (including a measure of health literacy) and then will be randomised to one of three interventions defined by a 3×2 factorial design: Volitional help sheet (semi-automated planning tool), Detailed plan (free-text planning tool) and Snacking tips sheet (passive control). Participants will be asked to make a copy of their plan, and after completion of the allocated tool, participants will be asked to complete an evaluation of the tool.

### Reminder messages (baseline, 1 week and 2 weeks):

One week from baseline, and after 1 week and 2 weeks, participants will receive a reminder of their plans (in the volitional help sheet or detailed plan conditions) or a copy of the snacking tip sheet (in the passive control condition).

### Follow-up questionnaire (one month):

One month from baseline, participants will complete a follow-up questionnaire.

### The three conditions:

#### Volitional help sheet:

This consists of 4 steps that guide the user through the process of developing an appropriate plan.

- Step1: Sometimes we snack because we are hungry, but there are lots of other reasons too. Think about your snacks in the last week. Below is a list of ‘snack moments.’ These are times when people tend to choose unhealthy snacks or eat too much. Choose 3 snack moments from the list that happened to you the most often in the last week. [List of snack moments].
- Step 2: Below are your top 3 snack moments. Some snack moments will be more important than others. Choose the 1 that you would be happiest to change. [User chooses from 3 previously selected snack moments]
- Step 3: Great! Your most important snack moment was snacking because you are bored.
- The last step is to come up with a plan! Choose the solution that you think will work best for you. Drag it into the space on the right. [List of solutions]
- Step 4: Imagine how your plan might feel.[examples of scenarios when this might happen]. The final step is to make sure the plan is realistic. How hard do you think it will be to do this plan for the next month [Slider from very easy to very hard. If the user selects a number 7 they will be prompted to revise the plan]

#### Detailed plan:

Text: We want you to plan how you will change your unhealthy snacking behaviour each day because forming plans has been shown to improve snacking habits.

You are free to choose how you do this but we want you to formulate your plans in as much detail as possible. Please pay attention to the situations in which you will implement (carry out) these plans. Focus on situations when you are not hungry but find yourself snacking.

#### Snacking tips sheet:

This is a modified version of the NDSS Healthy snacking tips sheet with references to diabetes, blood glucose and carbohydrate removed (see https://static.diabetesaustralia.com.au/s/fileassets/diabetes-australia/201fcd3d-3b7c-4f5f-a81a-46a200b1fa84.pdf ).

### Primary Outcome:

The main outcomes to be measured will be maintenance self-efficacy (3 items, 4-point Likert scale anchored to not at all true/exactly true) and unhealthy snacking scores (based on self-reported diet during previous week).

### Secondary Outcomes:

Secondary outcomes to be measured will be action control and self-regulatory effort, a 5 item 7-point Likert scale (strongly disagree to strongly agree).

### Details of how data will be analysed:

Randomisation will be tested using MANOVA (multivariate analysis of variance), to ensure similarity across the three groups of participants in terms of demographic and other descriptive characteristics at baseline. The effect of the planning tools (volitional help sheet; detailed planning; information sheet) will be assessed using ANCOVA (analysis of covariance), controlling for: baseline values, age, sex, and language spoken at home. Orthogonal planned contrasts will be used to test if the volitional help sheet is superior to the detailed planning intervention, and if both of these interventions are superior to the passive control intervention (information sheet) on the primary outcomes. Secondary analyses will include exploring possible mediation of behavioural change by maintenance self-efficacy by using bootstrapping procedures as outlined in Preacher and Hayes (2008; BehavResMethods 40: 879-891). The potential moderating effect of health literacy on the efficacy of planning tools will be explored by examining the interaction of intervention and health literacy in ANCOVA.

### Potential significance of the study:

This proposed study will provide new data on online planning tools to assist with health behaviour change. The results will be examined in relation to participants’ health literacy levels to ascertain whether this may be a useful tool for this subgroup. Furthermore, findings from this study will inform the design of an app for assisting patients with diabetes to manage their health behaviours, including snacking behaviour.
