# Supplementary material for: Randomized trial of planning tools to reduce unhealthy snacking: Implications for health literacy
Source: PLoS One. 2019 Jan 17;14(1):e0209863. doi: 10.1371/journal.pone.0209863 (PMC6336265; doi:10.1371/journal.pone.0209863)
Supplement: S6 File — (PDF) [file pone.0209863.s006.pdf]

THE UNIVERSITY OF SYDNEY

HUMAN RESEARCH ETHICS COMMITTEE FORM

Please Note:

This form was created via the University's online system (IRMA) and the information provided is recorded in the University's research office database.

This information is used to assess the ethics submission under the National Health and Medical Research Council's (NHMRCs) National Statement on Ethical Conduct in Human Research (2007) by the University Ethics Committee and its expert advisers, including the RPAH Clinical Trials Subcommittee.

Sign off by researchers is provided online in IRMA and will not be displayed in this document.

ADMINISTRATIVE DETAILS

Title: Online planning tool for unhealthy snacking

Chief Investigator: Prof Kirsten McCaffery

Primary Faculty/Department: School of Public Health: Public Health; Faculty of Medicine and Health

Investigators: McCaffery Kirsten; Ayre Julie; Bonner Carissa; McCaffery Kirsten;

Grants linked:

External Authorities:

Additional Information:

Snacks are important because they keep us going until the next meal. Sometimes though, we eat too many, or choose snacks that are unhealthy. Even though each snack is usually small, over time these snacks can add up. This can make us gain weight. Smart snacking means choosing nutritious, healthy snacks that give you energy until the next meal. While many of us want to change the way we snack, this can be very hard to do. Often we make plans but have trouble sticking to them over long periods of time. This study is testing a 'smart snacking' tool that will make sure that plans to snack healthily are high quality and easy to follow in the long term. To test the effect of this tool on snacking behaviour, we randomised participants to receive one of three planning tools: 1. Volitional help sheet (the online smart snacking planning tool)2. Directions to create a detailed plan3. Hints and tips to snack healthily

QUESTIONNAIRE

1 - <div align="left">Welcome to the University of Sydneyâ€™s Human Ethics Application Questionnaire. Please be aware that there is a limit of fifteen minutes to complete each individual question. If you exceed this time then your answer may not be saved by the system. We recommend

that you prepare long answers outside of IRMA before pasting it back into the report questionnaire and/or save your answers regularly.If you choose to edit a previous question, your responses to subsequent questions will be deleted. The restore button can be used to refill your subsequent answers if this happens.For further information on the application procedure, please consult our website or email the Human Ethics team at ro.humanethics@sydney.edu.auIf you experience any technical difficulties, please do not hesitate to contact Research Support using the details below:T +61 2 8627 8183E research.support@sydney.edu.au</div>

Continue

2 - SECTION ASection A is designed to distinguish between staff and student projects. In addition, this Section also seeks to identify projects that have been approved by other ethics committees.Is this project a University of Sydney student project ONLY (i.e. ethics application restricted to the activities of the student research project)?

Yes

4 - Select appropriate student classification:

PhD

3 - Indicate whether this project has been or will be submitted to any other ethics committees

No

5 - SECTION BSection B is designed to determine whether your study falls within the National Statementâ€™s definition of low or negligible risk. Throughout this section, you may be asked specific additional questions where you indicate that your study involves particular participant and/ or project types. Please note that the option â€œPossible Recruitmentâ€ with reference to specific participant populations indicates that these people MAY be recruited into your study, but are not the specific population of interest. If this population is the focus of your study, you should select â€œYesâ€. Please answer the following questionsDoes your research involve women who are pregnant and the human foetus?

Possible Recruitment

13 - Does your study involve children and/or young people (i.e. younger than 18 years)?

No

15 - Does your study involve people in existing dependent or unequal relationships with the researcher(s)?

No

29 - Does your research involve people with a cognitive impairment, an intellectual disability or a mental illness?

Possible Recruitment

33 - Does your research involve people highly dependent on medical care who may be unable to give consent?

No

38 - Does your study have the potential to discover illegal activity by participants or others? This includes research intending to expose illegal activity, as well as research not specifically designed to, but likely to discover, illegal activity.

No

41 - Does your research involve Aboriginal and/or Torres Strait Islander peoples?

Possible Recruitment

47 - Does your research involve CALD (Culturally and Linguistically Diverse) people?

Possible Recruitment

63 - Does your research involve travel overseas?

THE UNIVERSITY OF SYDNEY

No

64 - Is your study likely to cause or elicit distress in participants due to its subject matter, the procedures involved, information that might be revealed about the participant or related persons, or in some other way?

No

78 - Does your study involve research that could jeopardise a participant's employment?

No

79 - Is your proposed research a clinical trial? A clinical trial is a form of research designed to find out the effects of an intervention, including a treatment or diagnostic procedure. A clinical trial can involve testing a drug, a surgical procedure, other therapeutic procedures and devices, a preventive procedure, or a diagnostic device or procedure.

No

84 - Does your study involve the use of human tissue?

No

138 - Does your study involve human genetics or human stem cells?

No

185 - Does your study involve limited disclosure involving active concealment and/or planned deception?

No

186 - Does your study involve research that poses a risk to the physical or emotional safety or welfare of a University of Sydney student researcher (e.g. honours student or postgraduate student)? If you are a student and your research takes place off-campus a completion of a safety protocol may be necessary.

No

192 - Does your research involve any of the following: â€¢ Collection of biological samples (e.g. blood, saliva, bodily fluids).â€¢ Physical screening (e.g. blood pressure, cholesterol, physical fitness, MRI scans).â€¢ Physical exertion? (i.e. physical activity, exercise).

No

200 - Does the research ONLY involve existing collections of data or records about human beings (collected with appropriate ethical approval)?

No

201 - Is there a foreseeable risk of more than 'discomfort'? For a useful description of the differences between harm, discomfort and inconvenience please refer to the National Statement on Ethical Conduct in Human Research, Chapter 2.1

No

196 - SECTION CThe questions in Section C are designed to determine whether there are any conflicts of interests which may compromise the research process.Are any "conflict of interest" issues likely to arise in relation to this research?

No

209 - Do the researchers have any affiliation with, or financial involvement in, any organisation or entity with direct or indirect interests in the subject matter or materials of this research? (Note that such benefits must be declared in the Participant Information Statement)

No

211 - Do the researchers expect to obtain any direct or indirect financial or other benefits from conducting this research? (Note that such benefits must be declared in the Participant Information Statement)

No

213 - Have conditions already been imposed OR are likely to be imposed in the future, upon the use (e.g. publication), or ownership of the results (e.g. scientific presentations) or materials (e.g. audio-recordings), by any party other than the listed researchers?

No

215 - SECTION DThe questions in Section D are specifically directed at the consent process.Describe how you will identify and select potential participants for recruitment into the study. You should include information about how you will obtain contact details for potential participants.

Participants will be identified and selected by Qualtrics (Qualtrics, LLC), a 'software-as-a-service' company with experience in conducting survey research. Qualtrics has an extensive database of participants who are willing to be involved in online research. Participants selected for this experiment will be men and women aged 30 years and older. Half of all participants will have no university degree. All participants will be required to be able to read and write in English. No other quotas or restrictions will be placed on participant recruitment. In other words, participants will be representative of the Australian community in terms of age, gender, socioeconomic status etc. The University of Sydney will not obtain any contact details from potential participants.

217 - Describe how and where initial contact will be made with potential participants and how you will avoid real or perceived coercion. Copies of all relevant correspondence (e.g. email, letter of introduction, covering letter, circular/flyer etc.) need to be uploaded with your application. If you are using email addresses please outline how their use will not be in breach of privacy or spam legislation.

Initial contact will be made by Qualtrics, who will use their database to approach potential participants who meet the eligibility criteria. Participants listed on their database have already indicated a willingness to participate in online research. If participants agree and are interested in being part of the study, they will be directed to an online Participant Information Statement (attached), Consent Form (attached), and then the surveys themselves (attached).

218 - If a participant, or person on behalf of a participant, chooses to withdraw from the research, what specific consequences should they be made aware of, prior to giving consent? These details should be included in the Participant Information Statement.

Participants are free to withdraw from the study at any time. After reading the online Participant Information Statement, potential participants will be able elect to not proceed to the survey. It will be outlined in the Participation Information Statement that there will be no consequences to the individual should they wish to withdraw, and that they are able to withdraw from the survey at any point. In addition, participants who proceed to the survey and then discontinue survey completion part way through will be treated as though consent has been withdrawn, and their responses will not be used.

219 - Will participants receive any reimbursement of out-of-pocket expenses, or financial or other "rewards" as a result of participation?

Yes

221 - Specify the nature and value of any proposed incentive/payment (e.g. movie tickets, food vouchers) or reimbursement (e.g. travel expenses) to participants. Explain why this offer will not impair the voluntary nature of the consent, whether by participants or persons deciding for their behalf. Payment that is disproportionate to the time involved, or any other inducement that is likely to encourage participants to take risks, is ethically unacceptable. (See 2.2.10 and 2.2.11 of the National Statement). Note that monetary amounts should not be specified in advertisements, but payments should be disclosed in the Participant Information Statement in accordance with 2.2.6 (j) of the National Statement.

Participants will not receive any financial or other reward from the University of Sydney. Qualtrics, the company that will be recruiting the participants, have a points-system whereby points are earned for completion of surveys. Specifically, points are based on survey length rather than being offered for particular surveys. These points can be redeemed for various items such as shopping vouchers and airline vouchers. The points represent modest compensation for the time that participants forgo by participating in the study. Participants will complete two surveys, 1 month apart. They will receive points on completion of the first survey and then more points again after completing the second survey. To be able to complete a survey and to collect the points, the respondent needs to quality for a survey, i.e. profile match and pass all quality checks. The value of the points in itself is not

competitive with the minimum wage per hour, to avoid encouraging fake responses. This ensures that the completed surveys are quality-assured and less likely to be influenced by ‘incentives-skewed bias.

220 - How will consent be obtained (more than one may apply)

Return of a Survey

222 - Please clarify your response to the question above and justify with reference to the National Statement (e.g. sections 2.2.5, 3.1.16, 5.2.16). For instance, if you indicated that consent will be written and oral, does this refer to all participants undergoing written and oral consent or does it refer to different consent processes for different participant groups? You should also justify why you have chosen these forms of consent. If you are using oral consent, explain how it will be recorded (e.g. in field notes, using tape recording).

Participants will read an online Participant Information Statement followed by an online Consent Form. At the bottom of the Consent Form, participants will be asked “Do you consent”. They may click either ‘Yes’ or ‘No’. If participants click ‘Yes’ they will continue to the survey. If participants click ‘No’ they will not be able to continue to the survey. The University will only receive data from respondents that complete the surveys. To do so they have to have clicked ‘Yes’ at the Consent Form. Therefore, completing the survey will itself self as an indicator that they have consented (consistent with the NHMRC National Statement on Ethical Conduct in Human Research [2007]). Furthermore, participants, by virtue of being on the Qualtrics database, have already consented to being involved in online research.

223 - Will there be participants who are not fluent in English or who have difficulty understanding English?

No

224 - Will a Participant Information Statement be provided? If so, please attach this in the Documents tab.

Yes

230 - Is there an intention to recruit participants who have a physical impairment or disability that may affect the consent process (e.g. blind/vision impaired/deaf/hearing impaired/speech impaired)?

No

232 - SECTION EThe questions in Section E relate to how you will protect participantsâ€™ privacy and the confidentiality of their information in your research project. Will any part of the project involve recordings (e.g. audio, video, online surveys)?

Yes

235 - Which of the following recordings will be used in the project? More than one may apply.

Online surveys

236 - Outline how these recordings will be used and why they are necessary to achieve the aims of the research project. If your project involves online surveys, state where the surveys will be hosted and comment on any security, data ownership and privacy constraints associated with this survey host.

The online surveys will be designed in Qualtrics and distributed by Qualtrics. The surveys will capture participants’ responses and allow for quantification of the impact of an online planning tool on unhealthy snacking behaviour.

234 - Will you be collecting information/data about a participant from a third party (i.e. another individual)? Please note that this DOES NOT include agencies or organisations.

No

237 - The following questions will establish whether the HREC needs to apply federal or state/territory privacy legislation when reviewing your ethics application.Will you use, collect or disclose information about human participants from an agency, authority or organisation? This includes Commonwealth agencies, private sector organisations, state/territory agencies and international organisations. For instance, you may be using information from a medical practice, a hospital, a university, a state or

THE UNIVERSITY OF SYDNEY

federal government department. You should say â€˜yesâ€™ even if it is your own organisation (e.g. your medical practice).

No

241 - Is the research project likely to produce information or results that are of personal significance to individual participants? For instance, a project may reveal that participants are at risk of developing a particular disease, provide insight into their intellectual/other abilities, or indicate that they have physical or mental health problems.

No

335 - Is the research project likely to reveal a significant risk to the health or wellbeing of persons other than the participant (e.g. family members, colleagues, community members)?

No

319 - Does this project involve the use of information that you or your organisation had collected previously for another purpose?

No

344 - Describe how the overall results of this research project will be disseminated (e.g. journal publications and book chapters, conference presentations, student theses, creative works).

The results of the study will be disseminated through papers to be published in peer reviewed journals and/or local and international conferences.

347 - Will the confidentiality of participants and privacy of their data be protected in the dissemination of overall research results? Please note that if you propose to identify individuals in publications, you should select â€˜noâ€™ here and obtain their consent for this. Please also note that if you have obtained personal information without individual consent under a waiver of consent, you can only publish this information in de-identified form.

Yes

349 - Explain how confidentiality of participants and privacy of their data will be protected in the dissemination of research results.

There will be no respondent-identifying information that will be provided to researchers at the University of Sydney by Qualtrics. All analyses and results will be conducted using de-identified data. Furthermore, individual participants' responses are not of interest to this research question and researchers will only be analysing group results. The publications will report on the overall quantitative data of all participants.

350 - Will the information generated in this research project be used for any purpose(s) other than those outlined in this application? For example, will data be retained and used in future research projects, used to establish a database/research register, provided to a third party or to a public data sharing resource? Please note that this question does not refer to the use of the data for the purposes of this project (e.g. publication of results).

No

351 - Outline how feedback concerning the overall results of the project will be made available to participants (e.g. via a lay summary or newsletter). If participants are not to receive feedback, please justify why not.

As we will not collect participants' identifying information or contact details we will not provide results directly to respondents. Qualtrics will keep all respondent information, and if requested, can disseminate a lay summary of results to participants when available.

353 - Describe where study materials will be stored DURING the project (including electronic and hard copy files, consent forms, audio recordings, questionnaires, interview transcripts, video recordings, photographs etc). Please include building and room numbers for hard copy materials.

During the study, the survey data will be stored in password-protected files on a password-protected Research Data Store (Classic Research Data Store; RDS) – a storage solution provided and endorsed by the University of Sydney. These systems are regularly backed up, have built in redundancy, and are covered by the University's Information Security Policy. An RDS is the main

storage facility for research data at the University. There is unlimited storage and it is all stored on servers in Australia. Backed-up regularly with nightly backups kept for seven days, weekly backups kept for four weeks and monthly backups kept for twelve months Any hard copy materials will be stored in a locked filing cabinet in a locked office which can only be accessed by the Chief Investigator (Room 301F of the Edward Ford Building (A27) at the University of Sydney).

354 - Describe where study materials will be stored upon COMPLETION of the project (including electronic and hardcopy files, consent forms, audio recordings, questionnaires, interview transcripts, video recordings, photographs etc). Please include building and room numbers for hardcopy materials. Note that on conclusion of the project a copy of all materials must be kept in an accessible and secure location on University premises.

Upon completion of the study, the surveys will continue to be stored in password-protected files on a password-protected RDS. Any hard copy materials will continue to be stored in a locked filing cabinet in a locked office which can only be accessed by the Chief Investigator (Room 301F of the Edward Ford Building (A27) at the University of Sydney).

355 - Outline the security measures that will be used to protect study materials from misuse, loss or unauthorised access during and after the project (e.g. removal of identifiers, secure storage, restriction of access to appropriate personnel etc).

Study materials will be protected during and after the project as they will be stored in password-protected files on a password-protected RDS and kept in locked filing cabinets in locked offices in the Edward Ford Building (A27) at the University of Sydney.

356 - Specify how long study materials will be retained for after project completion. Please note that the options provided below are intended to facilitate compliance with relevant legislation from the State Records Authority of NSW. Data from research involving children; and from clinical trials, scanning and radioactivity studies, clinical studies, genetic manipulation, human tissue studies, and psychological research that has potential long term effects must be retained for a minimum of 20 years or until participants are 25 years of age (whichever is longer). Data from other types of studies must be retained for a minimum of 5 years. For some types of research (e.g. oral history, gene therapy) or where it is intended to reuse data in the future, it is appropriate to retain data in perpetuity (i.e. indefinitely).

5 years

357 - Explain why this storage period has been chosen.

Since this is a community survey, data will be stored for a minimum of 5 years as specified.

358 - At the end of the project, will study materials/information be stored in individually identifiable or re-identifiable form? Please note that this does not refer to the consent forms. Individually identifiable information is that from which the identity of a specific individual can reasonably be ascertained. Re-identifiable information has had identifiers removed and replaced by a code, so it is possible to identify individuals by using the code. Non-identifiable information has had all identifiers irreversibly removed or was never identifiable (see Chapter 3.2 of the National Statement for more information).

No

359 - If they are not to be kept in perpetuity, how will project materials ultimately be disposed of?

The materials for this study will ultimately be disposed of via deletion of any electronic documents or data related to the study. The IT department will be involved to ensure the deletion is complete and files are not retrievable from any source. Any hard-copy paper materials will be shredded.

361 - SECTION FThe questions in Section F concern risks to both participants and others connected with the study.Participation in research can involve potential harm to participants including physical, psychological, reputational, financial, spiritual, emotional and social distress. Please outline any potential harm and justify it with regard to the potential benefits of the project. What steps will the researchers take to minimise potential harm endured as a consequence of participation? (e.g. by providing access/information to counselling)

There is no foreseeable risk of potential harm to participants in the study. Participants are free to withdraw or stop the survey at any point and are not required to give an answer. Participants who want to improve their snacking behaviour will be provided with links to resources in the Debrief (attached) at the end of the survey.

362 - Are there any other risks involved in this research? For example, to the research team, the organisation, others? What are these risks? Explain how these risks will be negated/ minimised/ managed.

There are no other identifiable risks involved in this research.

363 - SECTION GThe questions in Section G concern details of the research study. Please answer the following questions.The nature of this project is most appropriately described as research involving (more than one may apply):

Questionnaire/survey

364 - Are you doing research in a context which requires you to get permission from an appropriate authority e.g. a school, corporation, NGO, or similar?

No

365 - Outline in lay language the theoretical, empirical and/or conceptual basis, background evidence for the research proposal with reference to the relevant literature (include at least four research citations). Note, that your study should be "based on a thorough study of the current literature, as well as previous studies" (NS 1.1 c).

Health literacy refers to the cognitive and social skills that an individual requires to understand and manage their health [1]. This is particularly relevant for health conditions that have a strong self-management component of care, including chronic diseases such as diabetes and cardiovascular disease [2]. Current theories of health literacy suggest this is because health literacy is important for factors that influence self-management, such as health knowledge, motivation, self-efficacy and problem-solving skills [1,3].

However, the evidence supporting these theories is mixed [4-7]. This is exemplified in research on type 2 diabetes. Reviews have reported consistent evidence of a relationship between health literacy and diabetes knowledge [8,9], and between self-management behaviour and glycaemic control, a key physiological outcome for diabetes [10,11]. However, evidence for a relationship between health literacy and glycaemic control and intermediary parameters such as self-efficacy (confidence in one's ability to achieve self-management goals) and self-management [8,9] are less consistent.

More broadly this research parallels a common observation in behaviour change research, the 'intention-behaviour gap' [12]. This is a discrepancy between an individual's intention (driven by knowledge, perceived risk and motivation) and their actual behaviour. Stated another way, intention alone may be insufficient to bring about behaviour change. This is further supported by meta-analyses of interventions for physical activity and healthy eating [13-18]. These studies concluded that the strategies based on control theory (such as self-monitoring, action planning and goal setting) increased the effectiveness of interventions.

The Health Action Process Approach (HAPA; [19]) model explicitly addresses the intention-behaviour gap. This model partitions health behaviour into two phases – a motivational and a volitional phase. In the motivational phase, individuals develop a behavioural intention, whilst in the volitional phase, individuals transition through to action (the latter phase encompasses many of the strategies based on control theory). Schwarzer [19] suggests that specific strategies are required to facilitate the shift into the volitional phase.

As such, the HAPA model highlights a potential limitation of current recommendations for health literacy resources. Although there are some general strategies to improve the health literacy of volitional tools, these have not been researched to the same extent as those aimed at increasing understanding and risk perception in lower health literacy populations. For example, the Universal Precautions Toolkit recommends the use of action plans [20,21], yet there has been little research considering how to optimise this tool for people with lower health literacy. Furthermore, reviews of health literacy interventions have reported that volitional (or empowerment) strategies tend to be underutilised and require further investigation [7,22].

A promising volitional strategy for people with lower health literacy is implementation intentions. An implementation intention (‘if-then’ planning) is a specific type of plan that consists of two parts: (1) the ‘if’ statement that specifies a situation; and (2) the ‘then’ statement that specifies the required action. There is strong evidence that implementation intentions can improve a range of behaviours such as healthy eating and physical activity [23]. There is also some evidence that these plans may be useful for people who have difficulty with cognitively demanding tasks (for example, those with low working memory [24], children with ADHD [25], and older adults [26]). One review suggests that these groups may benefit from implementation intentions because the cues to perform the behaviour are more accessible and demand less conscious deliberation for action [23]. As such, implementation intentions may be more effective in a lower health literacy population than other planning strategies.

In addition to this, some modes of delivery may be more cognitively challenging than others. For example, individuals are typically instructed to identify appropriate situations (‘if’ cues) and behaviours (‘then’ cues), then use these to generate plans as free-response text. The volitional help sheet is an alternative mode of delivery. This consists of two columns, one for situations and the other for relevant behaviours. The user then links the most personally relevant situation(s) and behaviour(s) to generate the implementation intentions. This approach increases the probability of a high quality plan whilst also automating some of the process. Several studies have shown positive effects of volitional help sheets on health behaviour compared to control groups, with improved physical activity, fruit intake, and diet, and reduced emotional eating [27-31].

The present study aims to build on this body of research and investigate whether a volitional help sheet is a useful tool for people with lower health literacy. The study will focus on snacking behaviour as this is a common issue that is relevant to the self-management of many chronic conditions. Several changes will be made to previous volitional help sheets to tailor the tool to a lower health literacy audience. This draws on existing recommendations for health literacy tools [20,32]. For example, the volitional help sheet will use simple language and an uncluttered layout. The tool will also be presented on an online platform to increase the potential for tailoring and automation. Lastly, the tool will use images to visually reinforce the implementation intention to reduce emphasis on the written word. This intervention will be compared to two common strategies to reduce unhealthy snacking: (1) instructions that ask the participant to create a detailed plan to change unhealthy snacking behaviour and (2) a fact sheet showing tips for healthy snacking.

References:

1. Sørensen K, Van den Broucke S, Fullam J, et al. Health literacy and public health: a systematic review and integration of definitions and models. BMC public health 2012; 12(1): 80.

2. Heijmans M, Waverijn G, Rademakers J, van der Vaart R, Rijken M. Functional, communicative and critical health literacy of chronic disease patients and their importance for self-management. Patient Education and Counseling 2015; 98(1): 41-8.

3. Paasche-Orlow MK, Wolf MS. The causal pathways linking health literacy to health outcomes. Am J Health Behav 2007; 31 Suppl 1: S19-26.

4. Berkman ND, Sheridan SL, Donahue KE, et al. Health literacy interventions and outcomes. Evidence report/technology assessment 2011; (199): 1-941.

5. Allen K, Zoellner J, Motley M, Estabrooks PA. Understanding the internal and external validity of health literacy interventions: a systematic literature review using the RE-AIM framework. Journal of health communication 2011; 16(sup3): 55-72.

6. Sheridan SL, Halpern DJ, Viera AJ, Berkman ND, Donahue KE, Crotty K. Interventions for Individuals with Low Health Literacy: A Systematic Review. Journal of Health Communication 2011; 16(sup3): 30-54.

7. Barry MM, DEath M, Sixsmith J. Interventions for Improving Population Health Literacy: Insights From a Rapid Review of the Evidence. Journal of Health Communication 2013; 18(12): 1507-22.

8. Al Sayah F, Majumdar SR, Williams B, Robertson S, Johnson JA. Health Literacy and Health Outcomes in Diabetes: A Systematic Review. Journal of General Internal Medicine 2013; 28(3): 444-52.

9. Bailey SC, Brega AG, Crutchfield TM, et al. Update on health literacy and diabetes. Diabetes Educ 2014; 40(5): 581-604.

10. Norris SL, Lau J, Smith SJ, Schmid CH, Engelgau MM. Self-Management Education for Adults With Type 2 Diabetes. A meta-analysis of the effect on glycemic control 2002; 25(7): 1159-71.

11. Schmitt A, Reimer A, Hermanns N, et al. Assessing Diabetes Self-Management with the Diabetes Self-Management Questionnaire (DSMQ) Can Help Analyse Behavioural Problems Related to Reduced Glycaemic Control. PLOS ONE 2016; 11(3): e0150774.

12. Sheeran P, Webb TL. The Intention–Behavior Gap. Social and Personality Psychology Compass 2016; 10(9): 503-18.

13. Michie S, Abraham C, Whittington C, McAteer J, Gupta S. Effective techniques in healthy eating and physical activity interventions: a meta-regression. Health Psychology 2009; 28(6): 690.

14. Lara J, Evans EH, O'Brien N, et al. Association of behaviour change techniques with effectiveness of dietary interventions among adults of retirement age: a systematic review and meta-analysis of randomised controlled trials. BMC Medicine 2014; 12(1): 177.

15. O'Brien N, McDonald S, Araújo-Soares V, et al. The features of interventions associated with long-term effectiveness of physical activity interventions in adults aged 55–70 years: a systematic review and meta-analysis. Health Psychology Review 2015; 9(4): 417-33.

16. Olander EK, Fletcher H, Williams S, Atkinson L, Turner A, French DP. What are the most effective techniques in changing obese individuals' physical activity self-efficacy and behaviour: a systematic review and meta-analysis. International Journal of Behavioral Nutrition and Physical Activity 2013; 10(1): 1.

17. Greaves CJ, Sheppard KE, Abraham C, et al. Systematic review of reviews of intervention components associated with increased effectiveness in dietary and physical activity interventions. BMC public health 2011; 11(1): 1.

18. McEwan D, Harden SM, Zumbo BD, et al. The effectiveness of multi-component goal setting interventions for changing physical activity behaviour: a systematic review and meta-analysis. Health Psychology Review 2016; 10(1): 67-88.

19. Schwarzer R. Modeling Health Behavior Change: How to Predict and Modify the Adoption and Maintenance of Health Behaviors. Applied Psychology 2008; 57(1): 1-29.

20. Quality AfHRa. Health Literacy Universal Precautions Toolkit, 2nd Edition. February 2015 2015. http://www.ahrq.gov/professionals/quality-patient-safety/quality-resources/tools/literacy-toolkit/healthlittoolkit2.html (accessed 14 June 2017.

21. DeWalt DA, Broucksou KA, Hawk V, et al. Developing and testing the health literacy universal precautions toolkit. Nursing Outlook 2011; 59(2): 85-94.

22. Kim SH, Lee A. Health-Literacy-Sensitive Diabetes Self-Management Interventions: A Systematic Review and Meta-Analysis. Worldviews on Evidence-Based Nursing 2016; 13(4): 324-33.

23. Hagger MS, Luszczynska A. Implementation Intention and Action Planning Interventions in Health Contexts: State of the Research and Proposals for the Way Forward. Applied Psychology: Health and Well-Being 2014; 6(1): 1-47.

24. Meeks JT, Pitães M, Brewer GA. The Compensatory Role of Implementation Intentions for Young Adults with Low Working Memory Capacity. Applied Cognitive Psychology 2015; 29(5): 691-701.

25. Gawrilow C, Gollwitzer PM, Oettingen G. If-Then Plans Benefit Executive Functions in Children with ADHD. Journal of Social and Clinical Psychology 2011; 30(6): 616-46.

26. Zimmermann TD, Meier B. The effect of implementation intentions on prospective memory performance across the lifespan. Applied Cognitive Psychology 2010; 24(5): 645-58.

27. Armitage CJ. Randomized test of a brief psychological intervention to reduce and prevent emotional eating in a community sample. Journal of Public Health 2015; 37(3): 438-44.

28. Armitage CJ. Field experiment of a very brief worksite intervention to improve nutrition among health care workers. Journal of Behavioral Medicine 2015; 38(4): 599-608.

29. Armitage CJ, Arden MA. A volitional help sheet to increase physical activity in people with low socioeconomic status: A randomised exploratory trial. Psychology & Health 2010; 25(10): 1129-45.

30. Armitage CJ, Norman P, Noor M, Alganem S, Arden MA. Evidence That a Very Brief Psychological Intervention Boosts Weight Loss in a Weight Loss Program. Behavior Therapy 2014; 45(5): 700-7.

31. Soureti A, Murray P, Cobain M, Chinapaw M, van Mechelen W, Hurling R. Exploratory Study of Web-Based Planning and Mobile Text Reminders in an Overweight Population. J Med Internet Res 2011; 13(4): e118.

32. OoDPaHP USDoHaHS. Health literacy online: A guide to simplifying the user experience. 2015.

367 - Outline in lay language the methodology for the research proposal. Note, that you study should be "designed or developed using methods appropriate for achieving the aims of the proposal" (NS 1.1 b). Your response should include:â€¢ Aims and hypotheses/research questionsâ€¢ Research plan including duration of the study and/or timelineâ€¢ Participant characteristics including sex, age range and inclusion/exclusion criteria (if relevant)â€¢ The intended sample size with a justification, and/or the particiant sampling/selection strategy (as relevant to your study)â€¢ Details of where the study will be undertaken (location/site/URL)â€¢ Details of how data will be collected and analysedâ€¢ Potential significance of the study

Aims and hypotheses/research question:

Aims:

The aims of the study are threefold: (1) to investigate the impact of an online planning tool on unhealthy snacking behaviour at one-month follow-up; (2) to compare this effect amongst higher and lower health literacy groups, and; (3) to investigate possible mediating factors or predictors of the effectiveness of the intervention, and how this relates to health literacy.

Hypotheses:

1. Participants using the volitional help sheet will have higher maintenance self-efficacy scores than participants using the detailed planning intervention, and participants in these groups (averaged) will have higher scores than participants in the passive control group.

2. Participants using the volitional help sheet will have lower unhealthy snacking scores than participants using the detailed planning intervention, and participants in these groups (averaged) will have lower scores than participants in the passive control group.

3. Change in behaviour will be mediated by maintenance self-efficacy

4. The effects of planning tool will be moderated by health literacy. It is hypothesised that the volitional help sheet will be more useful (i.e. reduce unhealthy snacking to a greater extent) for people with lower healthy literacy than the detailed planning intervention

Research plan including duration of the study and/or timeline:

It is estimated that recruitment and data collection will take three months (including a month interim period between the first and second survey). The study will be conducted via two online surveys (Qualtrics). The first survey will take approximately 20mins and the second approximately 10mins.

Participant characteristics including sex, age range and inclusion/exclusion criteria (if relevant):

A sample of 390 eligible participants will be recruited through Qualtrics, a 'software-as-a-service' company. They will recruit potential participants from their existing database until 390 eligible participants are identified based on screening questions to assess eligibility: at least 30 years of age, and sufficiently able to understand health-related information in English. Furthermore, in order to facilitate the involvement of people likely to be of lower health-literacy, half (50%) of all participants will have no university degree. Other than that, potential participants will be recruited to reflect the general population in terms of sex, age, cultural background, employment status, income.

The intended sample size with a justification, and/or the participant sampling/selection strategy (as relevant to your study):

The intended sample size is approximately 390 participants. Piloting the survey will allow for a more definite sample size to be determined. Eighty-seven participants randomized to each group (totalling 261 participants) will yield ~90% power to detect moderate effect sizes (Cohen's f = 0.25, corresponding to ANOVA effects and interaction as small as ?p 2 = 0.06) in the primary outcomes (maintenance self-efficacy scores; unhealthy snacking scores) and any secondary analyses, assuming a Bonferroni-adjusted two-sided alpha of 0.017 (allowing for four planned comparisons, and a correlation between the two primary outcome measures of ~ 0.25). Additionally, just under 50% additional cases will be recruited to account for potential missing values or attrition, totalling 390 participants (i.e., 130 per group).

Details of where the study will be undertaken (location/site/URL):

The online survey will be conducted via the Qualtrics website (www.qualtrics.com). Qualtrics will send participants specific URLs to the survey. The University of Sydney will not make any direct contact with the participants.

Details of how data will be collected:

Data will be collected directly from participants in the form of two online surveys. These will be spaced one-month apart. See the procedure below for further details as to data collection:

Procedure:

Baseline (questionnaire and intervention):

Participants will complete a series of baseline questionnaires and then will be randomised to one of three interventions defined by a 3×2 factorial design: Volitional help sheet (semi-automated planning tool), Detailed plan (free-text planning tool) and Snacking tips sheet (passive control). After completion of the allocated tool, participants will be asked to complete an evaluation of the tool and health literacy questionnaires.

Reminder message (1 week):

One week from baseline, participants will receive a reminder of their plans (in the volitional help sheet or detailed plan conditions) or a copy of the snacking tip sheet (in the passive control condition).

Follow-up questionnaire (one month):

One month from baseline, participants will complete a follow-up questionnaire.

The three conditions:

Volitional help sheet:

This consists of 4 steps that guide the user through the process of developing an appropriate plan.

• Step1: Sometimes we snack because we are hungry, but there are lots of other reasons too. Think about your snacks in the last week. Below is a list of 'snack moments.' These are times when people tend to choose unhealthy snacks or eat too much. Choose 3 snack moments from the list that happened to you the most often in the last week. [List of snack moments].

• Step 2: Below are your top 3 snack moments. Some snack moments will be more important than others. Choose the 1 that you would be happiest to change. [User chooses from 3 previously selected snack moments]

• Step 3: Great! Your most important snack moment was snacking because you are bored.

• The last step is to come up with a plan! Choose the solution that you think will work best for you. Drag it into the space on the right. [List of solutions]

• Step 4: Imagine how your plan might feel.[examples of scenarios when this might happen]. The final step is to make sure the plan is realistic. How hard do you think it will be to do this plan for the next month [Slider from very easy to very hard. If the user selects a number 7 they will be prompted to revise the plan]

Detailed plan:

Text: We want you to plan how you will change your unhealthy snacking behaviour each day because forming plans has been shown to improve snacking habits.

You are free to choose how you do this but we want you to formulate your plans in as much detail as possible. Please pay attention to the situations in which you will implement (carry out) these plans. Focus on situations when you are not hungry but find yourself snacking.

Snacking tips sheet:

This is a modified version of the NDSS Healthy snacking tips sheet with references to diabetes, blood glucose and carbohydrate removed (see https://static.diabetesaustralia.com.au/s/fileassets/diabetes-australia/201fcd3d-3b7c-4f5f-a81a-46a200b1fa84.pdf).

Primary Outcome:

The main outcomes to be measured will be maintenance self-efficacy (3 items, 4-point Likert scale anchored to not at all true/exactly true) and unhealthy snacking scores (based on self-reported diet during previous week).

Secondary Outcomes:

Secondary outcomes to be measured will be action control and self-regulatory effort, a 5 item 7-point Likert scale (strongly disagree to strongly agree).

Details of how data will be analysed:

Randomisation will be tested using MANOVA (multivariate analysis of variance), to ensure similarity across the three groups of participants in terms of demographic and other descriptive characteristics at baseline. The effect of the planning tools (volitional help sheet; detailed planning; information sheet) will be assessed using ANCOVA (analysis of covariance), controlling for: baseline values, age, sex, and language spoken at home. Orthogonal planned contrasts will be used to test if the volitional help sheet is superior to the detailed planning intervention, and if both of these interventions are superior to the passive control intervention (information sheet) on the primary outcomes. Secondary analyses will include exploring possible mediation of behavioural change by maintenance self-efficacy by using bootstrapping procedures as outlined in Preacher and Hayes (2008; BehavResMethods 40: 879-891). The potential moderating effect of health literacy on the efficacy of planning tools will be explored by examining the interaction of intervention and health literacy in ANCOVA.

Potential significance of the study:

This proposed study will provide new data on online planning tools to assist with health behaviour change. The results will be examined in relation to participants' health literacy levels to ascertain whether this may be a useful tool for this subgroup. Furthermore, findings from this study will inform the design of an app for assisting patients with diabetes to manage their health behaviours, including snacking behaviour.

207 - <div align="left">Thank you for completing the University of Sydneyâ€™s Human Ethics Application Questionnaire. After selecting 'Completed' below, please remember to attach any documents relevant to your application in the next tab. After completing your application, return to the 'Coversheet' tab and press the 'Submit' button. You will receive an email shortly after confirming your submission.Once again, if your require further information on the application procedure, please consult our website or email the Human Ethics team at ro.humanethics@sydney.edu.auIf you experience any technical difficulties, please do not hesitate to contact Research Support using the details below:T +61 2 8627 8183E research.support@sydney.edu.au</div>

Continue

LIST OF ATTACHED DOCUMENTS

Date Uploaded	Type	Document Name

22/06/2017	Questionnaires/Surveys	Baseline questionnaire (includes PIS and consent form)
22/06/2017	Other Type	Debriefing information sheet for participants
22/06/2017	Questionnaires/Surveys	Followup questionnaire
22/06/2017	Participant Info Statement	Participant information statement

Screen 1 – Project Title Screen

Smart snacking: An online planning tool
(Baseline survey)

A study conducted by the School of Public Health at the University of Sydney

Screen 1 – Participant information statement

Study Information Sheet:

Smart snacking: An online planning tool

Hello. We are from the School of Public Health at the University of Sydney. Our names are

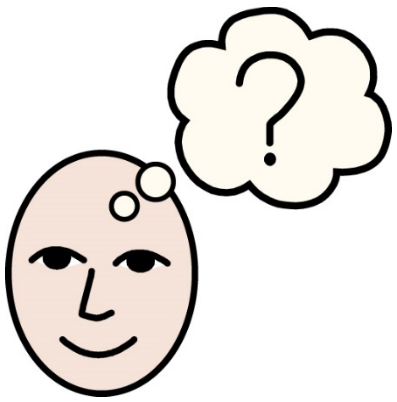

- Julie Ayre
- Dr Carissa Bonner
- Prof Kirsten McCaffery

We are doing a research study to find out more about tools to help people eat healthy snacks.

Snacks are important because they keep us going until the next meal. Sometimes though, we eat too many or choose snacks that are unhealthy. This can make us gain weight.

While many of us want to change the way we snack, this can be very hard to do. Often we make plans but have trouble sticking to them over long periods of time.

This study will look at online tools that help people stick to their plans.

We are asking you to be in our study because we are looking for people aged 30 years or more, who read and speak adequate English, and who would like to change the way they snack.

You can decide if you want to take part in the study or not. You don't have to - it's up to you.

This sheet tells you what we will ask you to do if you decide to take part in the study. Please read it carefully so that you can make up your mind about whether you want to take part.

You may stop completing the online survey at any point if you do not wish to continue, and we will not use your answers. You do not have to give a reason for not taking part. Once you have submitted your survey anonymously, your responses cannot be withdrawn.

If you have any questions, you can ask us or your family or someone else who looks after you. If you want to, you can call us any time on (02) 9351 7789.

What will happen if I say that I want to be in the study?

If you decide that you want to be in our study, we will ask you to do these things:

- Complete questions online about your demographics (for example, gender, age), the kinds of foods you eat and how you feel about your snacking behaviour
- Use the online planning tool to create a 'smart snacking' plan. You will receive a reminder message after 1 week.
- Try to follow the plan for one month, then complete some online questions about your snacking behaviour and your plan.

You can choose which questions you want to answer. If you don't want to give an answer, that's ok. You can stop answering questions at any time if you don't want to anymore.

This is an online study, so you can take part anywhere with access to the internet (smartphone or computer).

Will anyone else know what I say in the study?

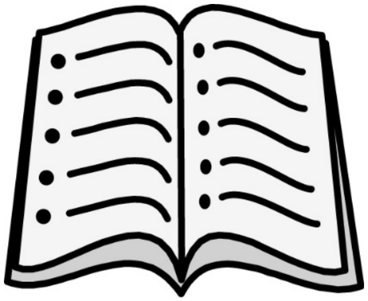

We won't tell anyone else what you say to us, except if you talk about someone hurting you or about you hurting yourself or someone else. Then we might need to tell someone to keep you and other people safe.

All of the information that we have about you from the study will be stored in a safe place and we will look after it very carefully. We will write a report about the study and show it to other people but we won't say your name in the report and no one will know that you were in the study, unless you tell us that it's ok for us to say your name.

How long will the study take?

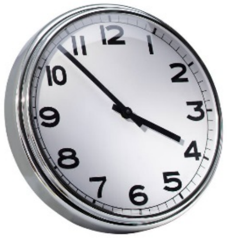

The first part of the study will take about 20 minutes to complete.

Over the following month you will be asked to try out your snacking plan.

The second part of the study will be sent to you after one month, and will take about 10 minutes to complete.

Are there any good things about being in the study?

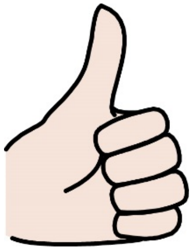

This study may help you think more about the way that you snack. This is the first step to changing your eating patterns. You may also find the tool useful for making these changes.

Are there any bad things about being in the study?

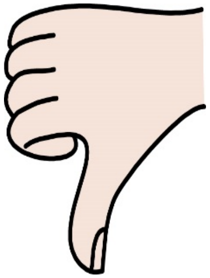

This study will take up some of your time, but we don't think it will be bad for you or cost you anything.

Will you tell me what you learnt in the study at the end?

Yes, we will if you want us to. There is a question on the next page that asks you if you want us to tell you what we learnt in the study. If you select Yes, when we finish the study we will tell you what we learnt.

What if I am not happy with the study or the people doing the study?

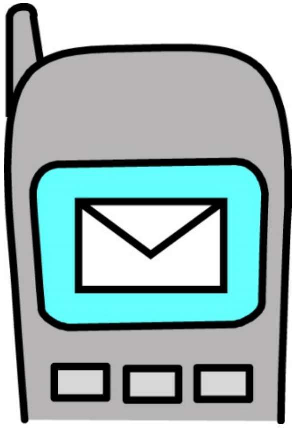

If you are not happy with how we are doing the study or how we treat you, then you or the person who looks after you can:

- **Call** the university on +61 2 8627 8176 or
- Write an **email** to human.ethics@sydney.edu.au

Screen 3 – Participant consent form

I give consent to my participation in the research project

TITLE: **Smart snacking: An online planning tool**

In giving my consent I acknowledge that:

1. The procedures required for the project and the time involved have been explained to me, and any questions I have about the project have been answered to my satisfaction.
2. I have read the Study Information Sheet and have been given the opportunity to discuss the information and my involvement in the project with the researcher/s.
3. I understand that being in this study is completely voluntary – I am not under any obligation to consent.
4. I understand that my involvement is strictly confidential. I understand that any research data gathered from the results of the study may be published however no information about me will be used in any way that is identifiable.
5. *I understand that I can withdraw from the study at any time, without affecting my treatment or my relationship with the researcher(s) or the University of Sydney now or in the future.*
6. I understand that I can stop my participation in this study at any time it I do not wish to continue and we will not use your answers.
7. *By completing the survey you have consented to be part of the study. Once you have submitted your survey anonymously, your responses cannot be withdrawn.*

I give my consent

Yes *No (if click no will not be directed to survey)*

Screen 4 – Baseline information and measures

Before we start please answer the following questions:

Age: [Select years from dropdown menu: 30/31/32/33 etc…]

Gender: [select gender from dropdown menu: Male/Female/Other]

English as first language: [select from dropdown menu: Yes/no]

Highest level of Education: [Select from dropdown menu: Less than high school/high school/Certificate I/II / Certificate III/IV, Diploma, Bachelor degree or equivalent, Masters or Doctoral degree or equivalent]

Smart snacking

Snacks are important because they keep us going until the next meal. Sometimes though, we eat too many, or choose snacks that are unhealthy. Even though each snack is usually small, over time the snacks add up. This can make us gain weight. **Smart snacking** means choosing nutritious, healthy snacks that give you energy until the next meal.

Which snacks are healthy?

Healthy snacks are low in kilojoules, fat, salt and sugars. These include fresh fruit, vegetables with dip, small amounts of dried fruit or nuts, yoghurt, coffee made with low fat milk, raisin toast, rice crackers and corn thins.

What are unhealthy snacks?

Unhealthy snacks are high in kilojoules, fat, salt and sugars. These include biscuits, cheese crackers, cakes, muffins, pastries, chocolate, lollies, potato chips, hot chips, French fries, some muesli bars and large coffees made with full cream milk.

Next

What are your snacking habits?

Before we get started on smart snacking, we'd like to know a little more about your snacking habits over the last month. For each question please answer by selecting a radio button.

Snacking habits

	Strongly disagree						Strongly agree
1. I eat unhealthy snacks frequently (all the time)	○	○	○	○	○	○	○
2. I eat unhealthy snacks automatically (without thinking)	○	○	○	○	○	○	○
3. I eat unhealthy snacks without having to consciously remember	○	○	○	○	○	○	○
4. I feel weird if I do not eat unhealthy snacks	○	○	○	○	○	○	○
5. I eat unhealthy snacks without thinking	○	○	○	○	○	○	○
6. It would require effort not to eat unhealthy snacks	○	○	○	○	○	○	○
7. Unhealthy snacks belong to (are part of) my (daily, weekly, monthly) routine	○	○	○	○	○	○	○
8. I start eating unhealthy snacks before I realise I'm doing it	○	○	○	○	○	○	○
9. I would find it hard not to eat unhealthy snacks	○	○	○	○	○	○	○
10. I don't need to think about unhealthy snacks	○	○	○	○	○	○	○
11. Unhealthy snacking is typical for me	○	○	○	○	○	○	○
12. I've been eating unhealthy snacks for a long time	○	○	○	○	○	○	○

Yesterday's snacks

Which snacks did you eat yesterday? Do not include food eaten during breakfast, lunch or dinner.

- ○ Hot chips, potato gems or French fries
- ○ Crackers, crisps or corn chips
- ○ Muffins, cake or doughnuts
- ○ biscuits
- ○ Pretzels
- ○ Pies, pasties or sausage rolls
- ○ Muesli bars, fruit bars, breakfast cereal bars
- ○ Chocolate
- ○ Lollies
- ○ Ice cream or ice blocks
- ○ Coffee with full cream milk
- ○ Coffee with skim milk
- ○ tea
- ○ Vita weat or Ryvita
- ○ Raisin toast
- ○ Apple or pear
- ○ Banana, mango
- ○ Orange or grapefruit
- ○ Kiwi fruit, mandarins
- ○ Yoghurt
- ○ Cherries, peaches or plums
- ○ Grapes or berries
- ○ Watermelon, melon
- ○ Carrot, cucumber or capsicum
- ○ Other fruit
- ○ Dip (e.g. hommos), cottage cheese or peanut butter
- ○ Nuts
- ○ Popcorn, rice crackers or corn thins
- ○ yoghurt
- ○ Other

Snacks in the last week

	Not at all						Very much
1. In the last week, too what extent (how much) have you eaten healthy snacks? (e.g. apple, banana, dried fruit)	○	○	○	○	○	○	○
2. In the last week, to what extent (how much) have you eaten unhealthy snacks? (e.g. chocolate, crisps, cake)	○	○	○	○	○	○	○

Snack diary for previous week

1. How often do you usually eat oven baked potato gems/chips/hashbrowns, hot chips/French fries, wedges or fried potatoes?

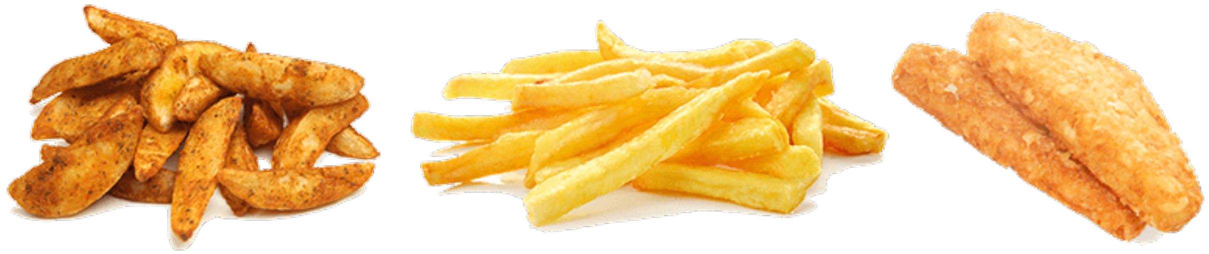

○ each week
○ each day
○ each month
○ I don’t eat this

In total, how many serves of potato gems/chips/hashbrowns, hot chips/French fries, wedges or fried potatoes do you usually eat in the timeframe selected above?

1 serve =
12 fried hot chips
60g potato gems/hashbrowns, or wedges

[slider for answer]

2. How often do you usually eat savoury snacks such as crisps, pretzels or plain/flavoured crackers?

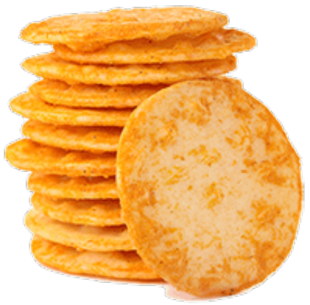 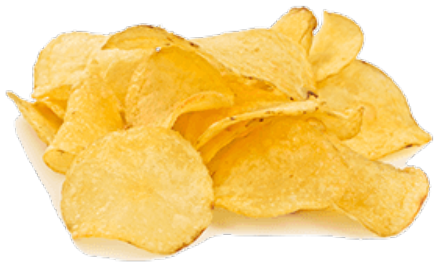 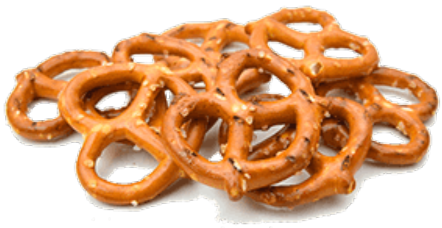

○ each week
○ each day
○ each month
○ I don’t eat this

In total, how many serves of savoury snacks such as crisps, pretzels or plain/flavoured crackers do you usually eat in the timeframe selected above?

1 serve =
½ snack size packet of crisps
30g of salty crackers or pretzels

[slider for answer]

3. How often do you usually have sweet biscuits/cakes/ buns/ muffins/ doughnuts? Include both home-made and bought.

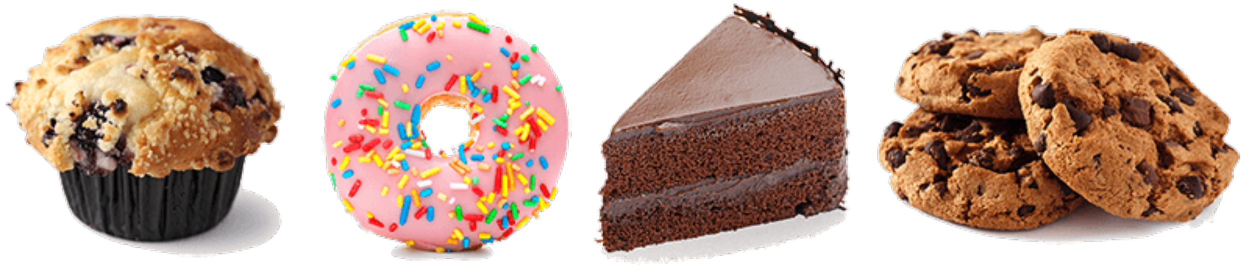

○ each week
○ each day
○ each month
○ I don't eat this

In total, how many serves of sweet biscuits/cakes/buns/muffins/doughnuts do you usually eat in the timeframe selected above?

1 serve =
2-3 (35g) sweet biscuits
1 doughnut
1 slice (40g) of plain cake or sweet bun
1 small muffin

[slider for answer]

4. How often do you usually eat savoury pastries?

This includes pies, pasties, sausage rolls, Kransky Dogs and frankfurters wrapped in pastry.

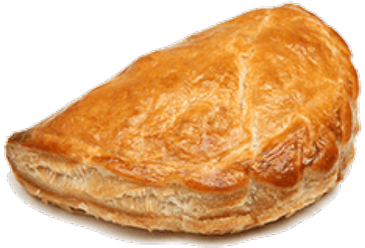
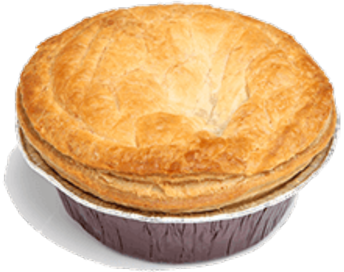
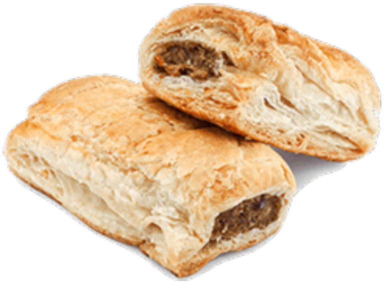

○ each week
○ each day
○ each month
○ I don't eat this

In total, how many serves of pies or savoury pastries do you usually eat in the timeframe selected above?

1 serve =
1/4 (60g) commerical meat pies or pastie
1 party size pie or sausage roll

[slider for answer]

5. How often do you usually eat snack type bars?

This includes muesli bars, fruit bars and breakfast cereal bars.

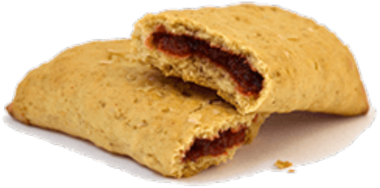 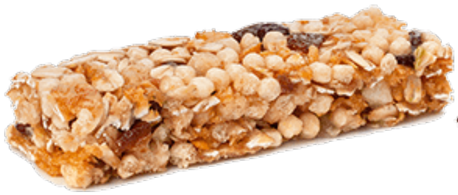 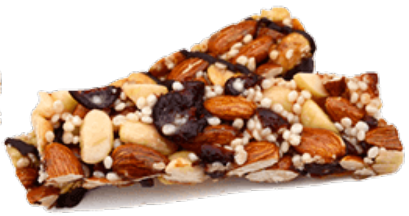

- ○ each week
- ○ each day
- ○ each month
- ○ I don’t eat this

In total, how many snack type bars do you usually eat in the timeframe selected above? This includes muesli bars, fruit bars and breakfast cereal bars.

[slider for answer]

6. How often do you usually have chocolate or lollies? *This question is required.

Include all types of chocolate and both hard and soft lollies.

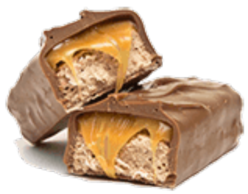 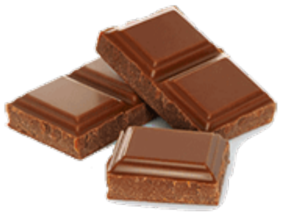 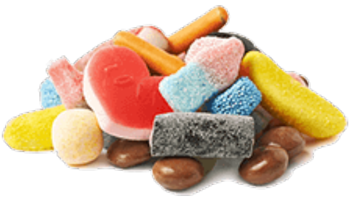 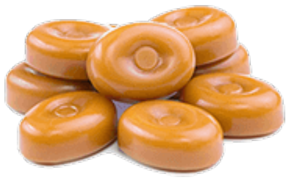

- ○ each week

○ each day
○ each month
○ I don’t eat this

In total, how many serves of chocolate or lollies do you usually eat in the timeframe selected above?

1 serve =
½ chocolate bar
4 pieces of chocolate (25g)
5-6 (40g) lollies

[slider for answer]

7. How often do you usually have ice-cream or ice-blocks?

This includes ice-blocks, ice-cream in a bowl or ice-creams on a stick.

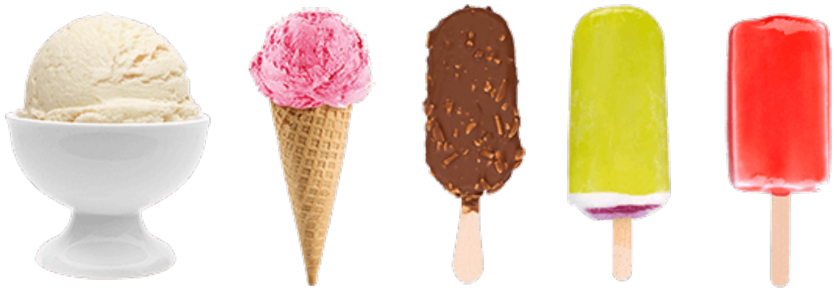

○ each week
○ each day
○ each month
○ I don’t eat this

In total, how many serves of ice-cream or ice-blocks do you usually eat in the timeframe selected above? *This question is required.

1 serve =
2 scoops (60g) ice-cream
1 stick ice-cream or ice-block

[slider for answer]

What do you think about snacking?

**If I eat healthier snacks…**	Not at all true	Barely true	Mostly true	Exactly true
1.I will feel healthier overall	○	○	○	○
2. I will feel better physically	○	○	○	○
3. I will have more energy	○	○	○	○
4. I will feel less hungry between meals	○	○	○	○
5. It will improve my body weight	○	○	○	○

	Strongly disagree						Strongly agree
6. Unhealthy snacking will make it harder to stay a healthy weight	○	○	○	○	○	○	○

**Over the next month:**	Strongly disagree	Strongly agree

7. I want to eat fewer unhealthy snacks	○	○	○	○	○	○	○
8. I plan to eat fewer unhealthy snacks	○	○	○	○	○	○	○
9. I intend to eat fewer unhealthy snacks	○	○	○	○	○	○	○

What snacking strategies do you already have?

**I already have clear plans about…**	Not at all true	Barely true	Mostly true	Exactly true
1. How I will change my unhealthy snacking habits	○	○	○	○
2. When I will change my unhealthy snacking habits	○	○	○	○
3. When I need to watch out so that I keep choosing healthy snacks	○	○	○	○
4. What to do in situations that make it hard to avoid unhealthy snacks	○	○	○	○
5. How to get back on track when I have eaten unhealthy snacks	○	○	○	○

How do you feel about changing your unhealthy snacking?

**I am sure that…**	Not at all true	Barely true	Mostly true	Exactly true

1. I can avoid eating unhealthy snacks for the next month	○	○	○	○
**I am certain that I can avoid eating unhealthy snacks even if…**				
2. Friends or family are eating unhealthy snacks	○	○	○	○
3. I am bored	○	○	○	○
4. I am craving an unhealthy snack	○	○	○	○

Nobody is perfect. Sometimes we have trouble sticking to our plans. Imagine you have started eating unhealthy snacks again. How confident are you about changing this habit?

**I am certain I could go back to eating healthy snacks…**				
5. Even after I ate 1 unhealthy snack	○	○	○	○
6. Even after a few days of eating unhealthy snacks	○	○	○	○
7. Even after a week of eating unhealthy snacks	○	○	○	○

Great! It looks like you're ready to **snack smarter**! The next step is to come up with a plan.

Let's get started!

The next section depends on intervention condition.

Screen 4a – Volitional help sheet condition

Smart snacking

Step 1: Snack moments

Sometimes we snack because we are hungry, but there are lots of other reasons too. Think about your snacks in the **last week**. Below is a list of '**snack moments**.' These are times when people tend to choose unhealthy snacks or eat too much. Choose **3** snack moments from the list that happened to you the **most often** in the last week.

The snack was in front of me	I had a craving	I was bored	I was tired	I could not stop at one piece
I was sitting in front of a TV or computer	Someone offered me the snack	People around me were eating	I was happy	I was drinking
I was busy or stressed	I arrived home	I was about to go to bed	I always have one with my tea or coffee	

Next

Step 2: The 'key' snack moment

Below are your top 3 snack moments. Some snack moments will be more important than others. Choose the **1** that you would be **happiest** to change.

I often snack when...

- I am bored
- I am sitting in front of a TV or computer
- people around me are eating

Next

Step 3: Make a plan

Great! Your most important snack moment was **snacking because you are bored [example text]**.
The last step is to come up with a plan! **Choose the solution that you think will work best for you**. Drag it into the space on the right

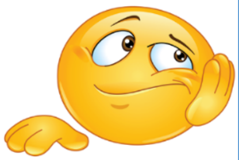

If I want a snack because I am bored → Then…

- I will go outside for a walk
- I will listen to music
- I will chat to someone for 5 minutes
- I will drink tea
- I will do a chore or task
- I will eat a smaller amount
- I will drink a large glass of water
- I will eat a piece of fruit
- I will take the food out of the packet and put it on a plate
- I will eat fresh vegetables and dip

Next

Step 4: Your plan is almost ready!

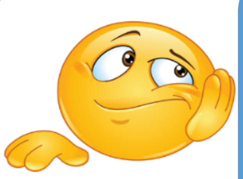

If I want a snack because I am bored

Then I will drink a large glass of water

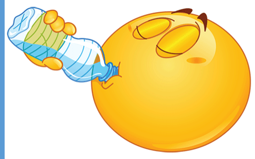

Imagine how your plan might feel. **When do you feel bored?** Here are some examples:

- waiting for a friend to arrive
- on a long train or bus trip
- watching TV
- doing long repetitive tasks

The final step is to make sure the plan is realistic.

How hard do you think it will be to do this plan for the next month?

Very easy Very hard

Next

[If score for previous question is <7/10]

Step 4: Your plan is almost ready!

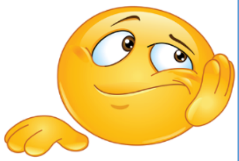

If I want a snack because I am bored

Then I will drink a large glass of water

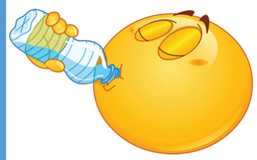

Your score shows that this plan may be quite hard for you. Would you like to choose an easier plan? We suggest you choose a different solution that you think will be easier to follow.

Change the snack moment

Change the solution

No thanks, I want to keep my plan

[If score for previous question is ≥7/10]

Your smart snacking plan

Well done! Try to remember this plan for the **next month**. Say it **3** times to yourself. You may also like to write the plan down or take a screenshot.

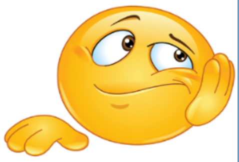

If I want a snack because I am bored

Then I will drink a large glass of water

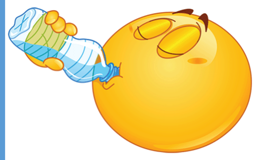

Take a screenshot

Next

Screen 4b – Detailed planning condition (active control)

Smart snacking

Your healthy snacking plan

We want you to plan how you will change your unhealthy snacking behaviour each day because forming plans has been shown to improve snacking habits.

You are free to choose how you do this but we want you to formulate your plans in as much detail as possible. Please pay attention to the **situations** in which you will implement (carry out) these plans. Focus on situations when you are not hungry but find yourself snacking.

Please enter your plan below
[Text box – e.g. When I am bored and hungry I will remember to drink a large glass of water first.]

How hard do you think it will be to do this plan for the next month?

Very easy Very hard

Next

Your healthy snacking plan

Well done! Try to remember this plan for the **next month**. Say it a few times to yourself. You may also like to write the plan down or take a screenshot.

[Example: When I am bored and hungry I will remember to drink a large glass of water first.]

Take a screenshot

Next

Screen 4c – Helpful hints sheet (passive control)

Smart snacking

Click the link below to read the 'healthy snacks' fact sheet. This will help you come up with a plan to choose healthier snacks [see attachment]. Once you have read the fact sheet, please answer the following question:

How hard do you think it will be to do this plan for the next month?

Very easy Very hard

Next

Screen 5 – Tool evaluation

Great work! Now you have a plan to try out for the next month.

We would like to ask a few more questions before you finish.

How difficult was the tool to use?

Extremely	Quite a bit	Somewhat	A little bit	Not at all
○	○	○	○	○

Screen 6 – Health literacy measures

1. How confident are you filling out medical forms by yourself?

Extremely	Quite a bit	Somewhat	A little bit	Not at all
○	○	○	○	○

Please read the nutritional panel below to answer the following questions. The panel is information on the back of a container or ice cream.

**Nutrition Facts**	
Serving Size	1/2 cup
Servings per container	4
Amount per serving	
Calories 250	Fat Cal 120
	%DV
**Total Fat** 13g	20%
Sat Fat 9g	40%
**Cholesterol** 28mg	12%
**Sodium** 55mg	2%
**Total Carbohydrate** 30g	12%
Dietary Fiber 2g	
Sugars 23g	
**Protein** 4g	8%

* Percent Daily Values (DV) are based on a 2,000 calorie diet. Your daily values may be higher or lower depending on your calorie needs.

Ingredients: Cream, Skim Milk, Liquid Sugar, Water, Egg Yolks, Brown Sugar, Milkfat, Peanut Oil, Sugar, Butter, Salt, Carrageenan, Vanilla Extract.

1. If you eat the entire container, how many calories will you eat?

2. If you are allowed to eat 60 grams of carbohydrates as a snack, how much ice cream could you have?

3. Your doctor advises you to reduce the amount of saturated fat in your diet. You usually have 42g of saturated fat each day, which includes one serving of ice cream. If you stop eating ice cream, how many grams of saturated fat would you be consuming each day?

4. If you usually eat 2,500 calories in a day, what percentage of your daily value of calories will you be eating if you eat one serving?

Pretend that you are allergic to the following substances: penicillin, peanuts, latex gloves, and bee stings.

5. Is it safe for you to eat this ice cream?
6. *[Ask only if patient responds 'no' to question 5]*: Why not?

On a scale from very difficult to very easy, how easy would you say it is to:

	Very difficult	Fairly difficult	Fairly easy	Very easy	Don’t know
1. Judge when you may need to get a second opinion from another doctor?	○	○	○	○	○
2. Use information the doctor gives you to make decisions about your illness?	○	○	○	○	○
3. Find information on how to manage mental health problems like stress or depression?	○	○	○	○	○
4. Judge if the information on health risks in the media is reliable?	○	○	○	○	○
5. Find out about activities that are good for your mental well-being?	○	○	○	○	○
6. Understand information in the media on how to get healthier?	○	○	○	○	○

That is the end of the survey, thank you for your participation.

You will be sent a planning reminder in one week.

After one month you will be asked to complete the final survey.

If you would like to receive a copy of the results of this study, please email the study office at julie.ayre@sydney.edu.au

Screen 1 – Project Title Screen

Smart snacking: An online planning tool

(Follow-up survey)

A study conducted by the School of Public Health at the University of Sydney

Screen 2 – Follow-up information and measures

Smart snacking

Snacks are important because they keep us going until the next meal. Sometimes though, we eat too many, or choose snacks that are unhealthy. Even though each snack is usually small, over time the snacks add up. This can make us gain weight. **Smart snacking** means choosing nutritious, healthy snacks that give you energy until the next meal.

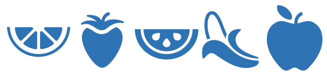

Which snacks are healthy?

Healthy snacks are low in kilojoules, fat, salt and sugars. These include fresh fruit, vegetables with dip, small amounts of dried fruit or nuts, yoghurt, coffee made with low fat milk, raisin toast, rice crackers and corn thins.

What are unhealthy snacks?

Unhealthy snacks are high in kilojoules, fat, salt and sugars. These include biscuits, cheese crackers, cakes, muffins, pastries, chocolate, lollies, potato chips, hot chips, French fries, some muesli bars and large coffees made with full cream milk.

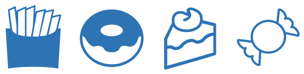

Next

What are your snacking habits?

We'd like to know a little more about your snacking habits over the last month. For each question please answer by selecting a radio button.

Snacking habits

	Strongly disagree						Strongly agree
1. I eat unhealthy snacks frequently (all the time)	○	○	○	○	○	○	○
2. I eat unhealthy snacks automatically (without thinking)	○	○	○	○	○	○	○
3. I eat unhealthy snacks without having to consciously remember	○	○	○	○	○	○	○
4. I feel weird if I do not eat unhealthy snacks	○	○	○	○	○	○	○
5. I eat unhealthy snacks without thinking	○	○	○	○	○	○	○
6. It would require effort not to eat unhealthy snacks	○	○	○	○	○	○	○
7. Unhealthy snacks belong to (are part of) my (daily, weekly, monthly) routine	○	○	○	○	○	○	○
8. I start eating unhealthy snacks before I realise I'm doing it	○	○	○	○	○	○	○
9. I would find it hard not to eat unhealthy snacks	○	○	○	○	○	○	○
10. I don't need to think about unhealthy snacks	○	○	○	○	○	○	○
11. Unhealthy snacking is typical for me	○	○	○	○	○	○	○
12. I've been eating unhealthy snacks for a long time	○	○	○	○	○	○	○

Yesterday's snacks

Which snacks did you eat yesterday? Do not include food eaten during breakfast, lunch or dinner.

- ○ Hot chips, potato gems or French fries
- ○ Crackers, crisps or corn chips
- ○ Muffins, cake or doughnuts
- ○ biscuits
- ○ Pretzels
- ○ Pies, pasties or sausage rolls
- ○ Muesli bars, fruit bars, breakfast cereal bars
- ○ Chocolate
- ○ Lollies
- ○ Ice cream or ice blocks
- ○ Coffee with full cream milk
- ○ Coffee with skim milk
- ○ tea
- ○ Vita weat or Ryvita
- ○ Raisin toast
- ○ Apple or pear
- ○ Banana, mango
- ○ Orange or grapefruit
- ○ Kiwi fruit, mandarins
- ○ Yoghurt
- ○ Cherries, peaches or plums
- ○ Grapes or berries
- ○ Watermelon, melon
- ○ Carrot, cucumber or capsicum
- ○ Other fruit
- ○ Dip (e.g. hommos), cottage cheese or peanut butter
- ○ Nuts
- ○ Popcorn, rice crackers or corn thins
- ○ yoghurt
- ○ Other

Snacks in the last week

	Not at all						Very much
1. In the last week, too what extent (how much) have you eaten healthy snacks? (e.g. apple, banana, dried fruit)	○	○	○	○	○	○	○
2. In the last week, to what extent (how much) have you eaten unhealthy snacks? (e.g. chocolate, crisps, cake)	○	○	○	○	○	○	○

Snack diary for previous week

1. How often do you usually eat oven baked potato gems/chips/hashbrowns, hot chips/French fries, wedges or fried potatoes?

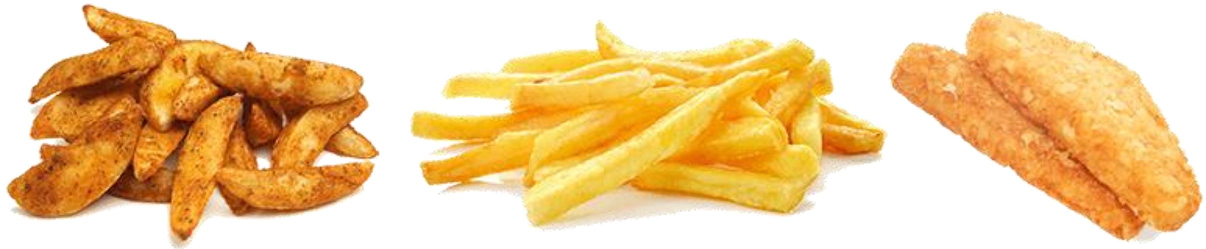

○ each week
○ each day
○ each month
○ I don’t eat this

In total, how many serves of potato gems/chips/hashbrowns, hot chips/French fries, wedges or fried potatoes do you usually eat in the timeframe selected above?

1 serve =
12 fried hot chips
60g potato gems/hashbrowns, or wedges

[slider for answer]

2. How often do you usually eat savoury snacks such as crisps, pretzels or plain/flavoured crackers?

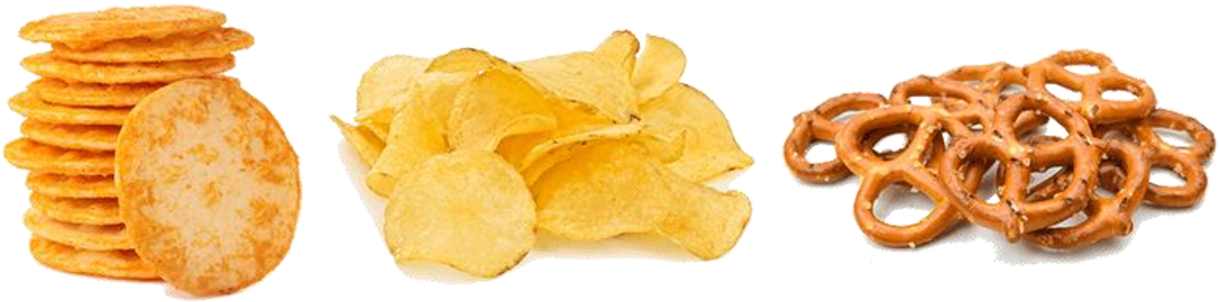

○ each week
○ each day
○ each month
○ I don’t eat this

In total, how many serves of savoury snacks such as crisps, pretzels or plain/flavoured crackers do you usually eat in the timeframe selected above?

1 serve =
½ snack size packet of crisps
30g of salty crackers or pretzels

[slider for answer]

3. How often do you usually have sweet biscuits/cakes/ buns/ muffins/ doughnuts? Include both home-made and bought.

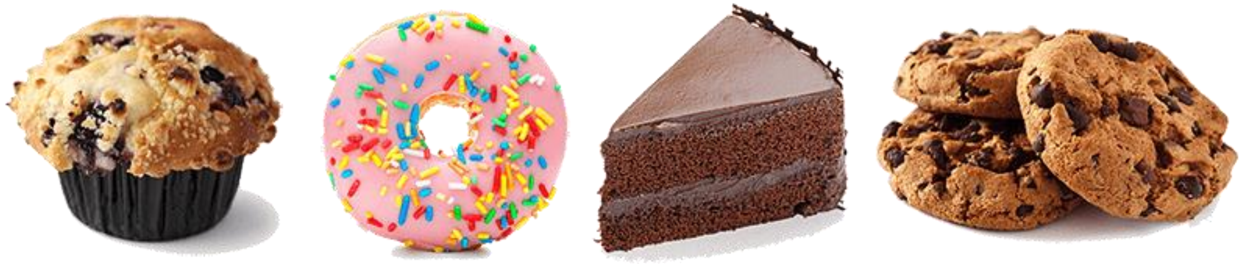

○ each week
○ each day
○ each month
○ I don’t eat this

In total, how many serves of sweet biscuits/cakes/buns/muffins/doughnuts do you usually eat in the timeframe selected above?

1 serve =
2-3 (35g) sweet biscuits
1 doughnut
1 slice (40g) of plain cake or sweet bun
1 small muffin

[slider for answer]

4. How often do you usually eat savoury pastries?

This includes pies, pasties, sausage rolls, Kransky Dogs and frankfurters wrapped in pastry.

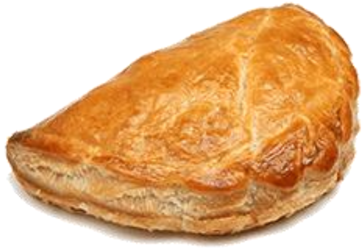 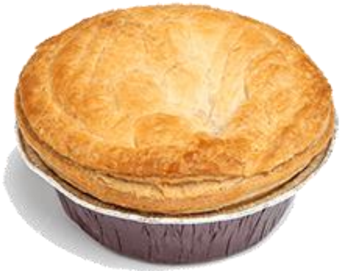 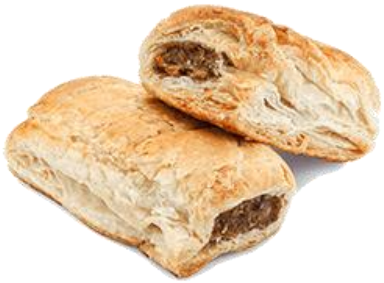

○ each week
○ each day
○ each month
○ I don’t eat this

In total, how many serves of pies or savoury pastries do you usually eat in the timeframe selected above?

1 serve =
1/4 (60g) commerical meat pies or pastie
1 party size pie or sausage roll

[slider for answer]

5. How often do you usually eat snack type bars?

This includes muesli bars, fruit bars and breakfast cereal bars.

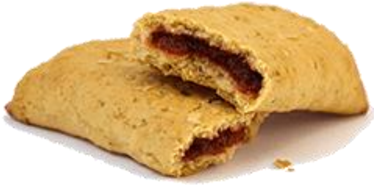 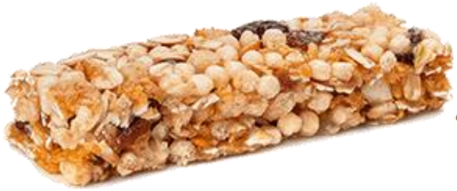 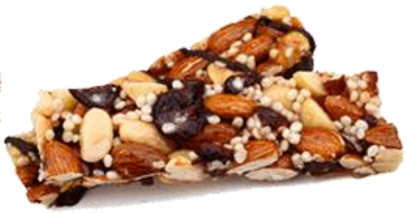

○ each week
○ each day
○ each month
○ I don't eat this

In total, how many snack type bars do you usually eat in the timeframe selected above?
This includes muesli bars, fruit bars and breakfast cereal bars.

[slider for answer]

6. How often do you usually have chocolate or lollies? *This question is required.
Include all types of chocolate and both hard and soft lollies.

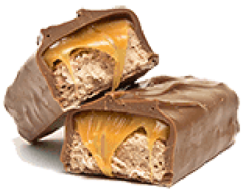 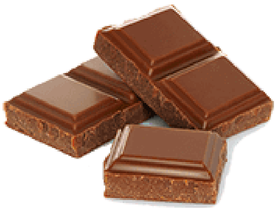 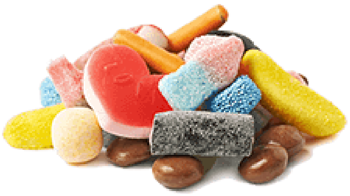 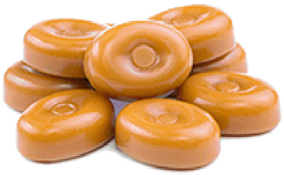

○ each week

○ each day
○ each month
○ I don’t eat this

In total, how many serves of chocolate or lollies do you usually eat in the timeframe selected above?

1 serve =
½ chocolate bar
4 pieces of chocolate (25g)
5-6 (40g) lollies

[slider for answer]

7. How often do you usually have ice-cream or ice-blocks?

This includes ice-blocks, ice-cream in a bowl or ice-creams on a stick.

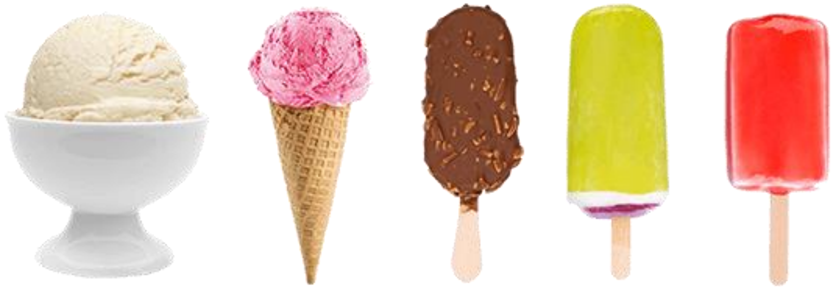

○ each week
○ each day
○ each month
○ I don’t eat this

In total, how many serves of ice-cream or ice-blocks do you usually eat in the timeframe selected above? *This question is required.

1 serve =
2 scoops (60g) ice-cream
1 stick ice-cream or ice-block

[slider for answer]

What do you think about snacking?

**If I eat healthier snacks…**	Not at all true	Barely true	Mostly true	Exactly true
1.I will feel healthier overall	○	○	○	○
2. I will feel better physically	○	○	○	○
3. I will have more energy	○	○	○	○
4. I will feel less hungry between meals	○	○	○	○
5. It will improve my body weight	○	○	○	○

	Strongly disagree						Strongly agree
6. Unhealthy snacking will make it harder to stay a healthy weight	○	○	○	○	○	○	○

Over the next month: Strongly disagree Strongly agree

7. I want to eat fewer unhealthy snacks	○	○	○	○	○	○	○
8. I plan to eat fewer unhealthy snacks	○	○	○	○	○	○	○
9. I intend to eat fewer unhealthy snacks	○	○	○	○	○	○	○

What snacking strategies do you have?

**I have clear plans about…**	Not at all true	Barely true	Mostly true	Exactly true
1. How I will change my unhealthy snacking habits	○	○	○	○
2. When I will change my unhealthy snacking habits	○	○	○	○
3. When I need to watch out so that I keep choosing healthy snacks	○	○	○	○
4. What to do in situations that make it hard to avoid unhealthy snacks	○	○	○	○
5. How to get back on track when I have eaten unhealthy snacks	○	○	○	○

How do you feel about changing your unhealthy snacking?

**I am sure that…**	Not at all true	Barely true	Mostly true	Exactly true

1. I can avoid eating unhealthy snacks for the next month ○ ○ ○ ○

I am certain that I can avoid eating unhealthy snacks even if…

2. Friends or family are eating unhealthy snacks ○ ○ ○ ○
3. I am bored ○ ○ ○ ○
4. I am craving an unhealthy snack ○ ○ ○ ○

Nobody is perfect. Sometimes we have trouble sticking to our plans. Imagine you have started eating unhealthy snacks again. How confident are you about changing this habit?

I am certain I could go back to eating healthy snacks…

5. Even after I ate 1 unhealthy snack ○ ○ ○ ○
6. Even after a few days of eating unhealthy snacks ○ ○ ○ ○
7. Even after a week of eating unhealthy snacks ○ ○ ○ ○

How did you was your snacking plan?

**During the last month…**	Strongly disagree						Strongly agree
I often thought about my plan to reduce the number of unhealthy snacks I ate each day	○	○	○	○	○	○	○
I constantly kept count of how many unhealthy snacks I ate to make sure it was not too much	○	○	○	○	○	○	○

I reminded myself to make sure I wasn't having too many unhealthy snacks	○	○	○	○	○	○	○
I tried my best to be consistent with my plan to eat less unhealthy snacks	○	○	○	○	○	○	○
I really tried to reduce the number of unhealthy snacks I ate each day	○	○	○	○	○	○	○

That is the end of the survey, thank you for your participation.

You will be sent a planning reminder in one week.

After one month you will be asked to complete the final survey.

If you would like to receive a copy of the results of this study, please email the study office at julie.ayre@sydney.edu.au

Study Information Sheet:
Smart snacking: An online planning tool

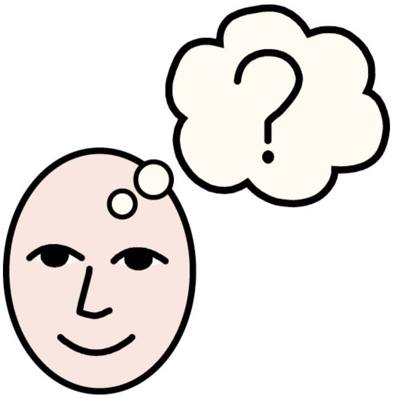

Hello. We are from the School of Public Health at the University of Sydney. Our names are

- Julie Ayre
- Carissa Bonner
- Kirsten McCaffery

We are doing a research study to find out more about tools to help people eat healthy snacks.

Snacks are important because they keep us going until the next meal. Sometimes though, we eat too many or choose snacks that are unhealthy. This can make us gain weight.

While many of us want to change the way we snack, this can be very hard to do. Often we make plans but have trouble sticking to them over long periods of time.
This study will look at online tools that help people stick to their plans.

We are asking you to be in our study because we are looking for people aged 30 years or more, who read and speak adequate English, and who would like to change the way they snack.

You can decide if you want to take part in the study or not. You don't have to - it's up to you.

This sheet tells you what we will ask you to do if you decide to take part in the study. Please read it carefully so that you can make up your mind about whether you want to take part.

You may stop completing the online survey at any point if you do not wish to continue, and we will not use your answers. You do not have to give a reason for not taking part. Once you have submitted your survey anonymously, your responses cannot be withdrawn.

If you have any questions, you can ask us or your family or someone else who looks after you. If you want to, you can call us any time on (02) 9351 7789.

What will happen if I say that I want to be in the study?

If you decide that you want to be in our study, we will ask you to do these things:

- Complete questions online about your demographics (for example, gender, age), the kinds of foods you eat and how you feel about your snacking behaviour
- Use the online planning tool to create a 'smart snacking' plan. You will receive a reminder message after 1 week.

- Try to follow the plan for one month, then complete some online questions about your snacking behaviour and your plan.

You can choose which questions you want to answer. If you don't want to give an answer, that's ok. You can stop answering questions at any time if you don't want to anymore.

This is an online study, so you can take part anywhere with access to the internet (smartphone or computer).

Will anyone else know what I say in the study?

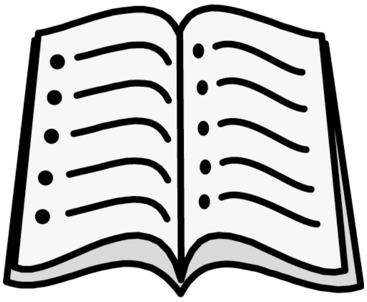

We won't tell anyone else what you say to us, except if you talk about someone hurting you or about you hurting yourself or someone else. Then we might need to tell someone to keep you and other people safe.

All of the information that we have about you from the study will be stored in a safe place and we will look after it very carefully. We will write a report about the study and show it to other people but we won't say your name in the report and no one will know that you were in the study, unless you tell us that it's ok for us to say your name.

How long will the study take?

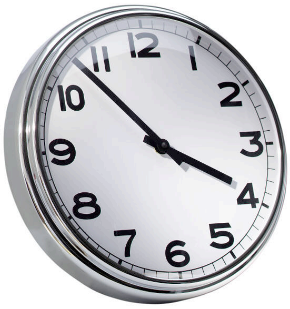

The first part of the study will take about 20 minutes to complete.

Over the following month you will be asked to try out your snacking plan.

The second part of the study will be sent to you after one month, and will take about 10 minutes to complete.

Are there any good things about being in the study?

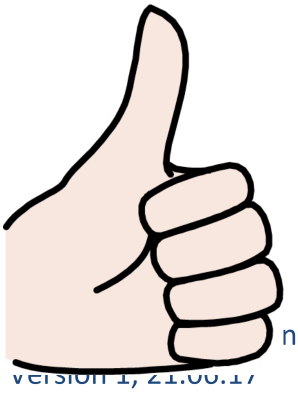

This study may help you think more about the way that you snack. This is the first step to changing your eating patterns. You may also find the tool useful for making these changes.

Are there any bad things about being in the study?

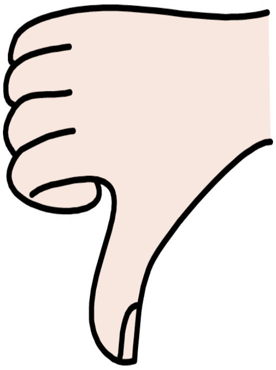

This study will take up some of your time, but we don't think it will be bad for you or cost you anything.

Will you tell me what you learnt in the study at the end?

Yes, we will if you want us to. There is a question on the next page that asks you if you want us to tell you what we learnt in the study. If you select Yes, when we finish the study we will tell you what we learnt.

What if I am not happy with the study or the people doing the study?

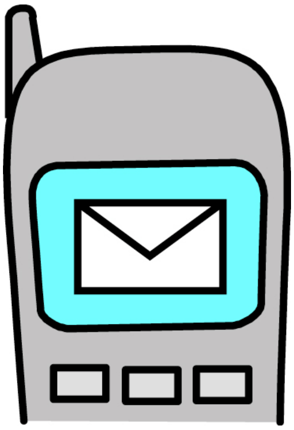

If you are not happy with how we are doing the study or how we treat you, then you or the person who looks after you can:

- **Call** the university on +61 2 8627 8176 or
- Write an **email** to human.ethics@sydney.edu.au

This sheet is for you to keep.

Smart snacking: An online planning tool

About the study

Snacks are important because they keep us going until the next meal. Sometimes though, we eat too many, or choose snacks that are unhealthy. Even though each snack is usually small, over time these snacks can add up. This can make us gain weight. Smart snacking means choosing nutritious, healthy snacks that give you energy until the next meal.

While many of us *want* to change the way we snack, this can be very hard to do. Often we make plans but have trouble sticking to them over long periods of time. This study is testing a 'smart snacking' tool that will make sure that plans to snack healthily are high quality and easy to follow in the long term.

To test the effect of this tool on snacking behaviour, we randomised participants to receive one of three planning tools:

1. Volitional help sheet (the online smart snacking planning tool)
2. Directions to create a detailed plan
3. Hints and tips to snack healthily

We are interested in finding out if the volitional help sheet helps people to snack more healthily, compared to the other two conditions. We will also examine whether this tool helps people feel more confident when they come up against barriers or problems changing their snacking behaviour. Lastly, we will see whether this tool is better suited to people with lower health literacy.

Further information on smart snacking

- Baker IDI Healthy Snacks Fact Sheet
- Make Healthy Normal Healthy Snacking Tips
- Eat for Health (Australian Dietary Guidelines)

How can I improve my snacking?

On the next page is a paper version of the volitional help sheet. Start off with one plan and you can gradually add more as these become habits. The tool is based on 'implementation intentions,' a type of plan that interrupts habits and builds new ones.

Smart snacking

Snacks are important because they keep us going until the next meal. Sometimes though, we eat too many, or choose snacks that are unhealthy. Even though each snack is usually small, over time the snacks add up. This can make us gain weight. **Smart snacking** means choosing nutritious, healthy snacks that give you energy until the next meal.

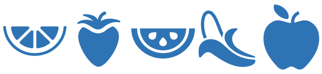

Which snacks are healthy?

Healthy snacks are low in kilojoules, fat, salt and sugars. These include fresh fruit, vegetables with dip, small amounts of dried fruit or nuts, yoghurt, coffee made with low fat milk, raisin toast, rice crackers and corn thins.

What are unhealthy snacks?

Unhealthy snacks are high in kilojoules, fat, salt and sugars. These include biscuits, cheese crackers, cakes, muffins, pastries, chocolate, lollies, potato chips, hot chips, French fries, some muesli bars and large coffees made with full cream milk.

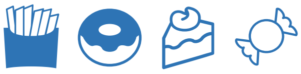

Step 1: Snack moments

Sometimes we snack because we are hungry, but there are lots of other reasons too. Think about your snacks in the **last week.** On the next page there is a list of '**snack moments**.' These are times when people tend to choose unhealthy snacks or eat too much.

Choose **3** snack moments from the list that happened to you the most often in the last week. (Place a ✓ on the left).

Step 2: The 'key' snack moment

Some snack moments will be more important than others. Choose **1** snack moment above that you would be happiest to change. Place a star next to this snack moment.

Step 3: Make a plan

Great! The next step is to come up with a plan! Choose the solution that you think will work best for you. **Draw a line** between your key snack moment and your solution.

Step 4: Your plan is almost ready!

Imagine how your plan might feel. When does the snack moment usually happen?

The final step is to make sure the plan is realistic:

How hard do you think it will be to do this plan for the next month?

Very easy									Very hard
1	2	3	4	5	6	7	8	9	10
○	○	○	○	○	○	○	○	○	○

If you scored 7 or more then this plan may be quite hard for you. We suggest you choose a different solution that you think will be easier to follow.

Steps 1 – 4: Creating the plan

**Snack moments**<br>(Over the last week I often snacked when…)	**Solutions**<br>(Instead of an unhealthy snack…)
The snack was in front of me	I will go outside for a walk
I had a craving	I will listen to music
I was bored	I will chat to someone for 5 minutes
I was tired	I will drink tea
I could not stop at one piece	I will do a chore or task
I was happy	I will eat a smaller amount
I was sitting in front of a TV or computer	I will drink a large glass of water
I was drinking	I will eat a piece of fruit
I was about to go to bed	I will take the food out of the packet and put it on a plate
Someone offered me the snack	I will eat fresh vegetables and dip
I was busy or stressed	
I always have one with my tea or coffee	
People around me were eating	
I arrived home	

Your smart snacking plan

Well done! Write the plan down in the space below. Say it 3 times to yourself. Try to remember this plan for the **next month**.

If I want a snack because: ____

Then I will: ____

HUMAN RESEARCH ETHICS COMMITTEE FORM

Please Note:

This form was created via the University's online system (IRMA) and the information provided is recorded in the University's research office database.

This information is used to assess the ethics submission under the National Health and Medical Research Council's (NHMRCs) National Statement on Ethical Conduct in Human Research (2007) by the University Ethics Committee and its expert advisers, including the RPAH Clinical Trials Subcommittee.

Sign off by researchers is provided online in IRMA and will not be displayed in this document.

ADMINISTRATIVE DETAILS

Title: Online planning tool for unhealthy snacking

Chief Investigator: Prof Kirsten McCaffery

Primary Faculty/Department: School of Public Health: Public Health; Faculty of Medicine and Health

Investigators: McCaffery Kirsten; Ayre Julie; Bonner Carissa;

Grants linked:

External Authorities:

Additional Information:

Please see attached documents including cover letter response to request for ethics modifications.

QUESTIONNAIRE

LIST OF ATTACHED DOCUMENTS

Date Uploaded	Type	Document Name
14/08/2017	Cover Letter/Correspondence	cover letter
14/08/2017	Participant Info Statement	Participant information statement vs 2 (clean)

14/08/2017	Participant Info Statement	Participant information statement vs 2 (tracked changes)
14/08/2017	Questionnaires/Surveys	Smart snacking planning tool baseline v2 (clean)
14/08/2017	Questionnaires/Surveys	Smart snacking planning tool baseline v2 (tracked changes)
14/08/2017	Questionnaires/Surveys	Smart snacking planning tool followup v2 (clean)
14/08/2017	Questionnaires/Surveys	Smart snacking planning tool followup v2 (tracked changes)
14/08/2017	Other Type	SSI recruitment details

THE UNIVERSITY OF SYDNEY

School of Public Health
Faculty of Medicine

ABN 15 211 513 464

PROFESSOR Kirsten McCaffery
School of Public Health

Room 301F
Edward Ford Building (A27)
The University of Sydney
NSW 2006 AUSTRALIA
Telephone: +61 2 9351 7220
Email: Kirsten.mccaffery@sydney.edu.au
Web: http://www.sydney.edu.au/

Friday August 11, 2017

Human Research Ethics Committee (Category B Chair)

Re: Project No. 2017/662 – "Online planning tool for unhealthy snacking"

Thank you for your response to the above study application reviewed by the Human Ethics Review Committee on 17 July 2017.

Please find attached a detailed response to each point. This has resulted in changes to the following documents (see tracked changes or clean documents):

- **PIS easy v2**
- **Smart snacking planning tool baseline v2**
- **Smart snacking planning tool follow up v2**

In addition, piloting and discussion with the recruitment company SSI indicated the need for some minor changes to the planning tool and survey questions which are outlined in the attached response.

We look forward to hearing back from you.

Sincerely,

KJ McCaffery

Professor Kirsten McCaffery
School of Public Health
University of Sydney

Data collection and storage

1. *Q367 of the application questionnaire states "The online survey will be conducted via the Qualtrics website". The Committee was unclear as to the security of the Qualtrics website and therefore raised concern regarding the collection, transfer and storage of participant data using this platform.*

a. Please comment on the security of the Qualtrics website for the collection and storage of participant data.

b. Please clarify how the participant data is managed by Qualtrics. (E.g. Does Qualtrics keep a record of all collected data once they send the data to the researchers? For how long? When and how do they securely destroy the participant data from their system?).

Qualtrics will host the survey, and Survey Sampling International (SSI) will be used for recruitment of participants. Only SSI will have access to participant details (e.g. names and email addresses). Participants will be allocated an ID (provided to SSI) and this will be used to link baseline and follow up survey data. The data stored on Qualtrics' server will therefore be de-identified and cannot be linked to participant details. SSI will not have access to the survey results as these are stored in the Qualtrics server in the specific School of Public Health account used for this project. The investigators on this project are the only individuals with access to this account.

The Qualtrics servers are protected by high-end firewall systems, secure data centres, and the company does not sell or make available specific information about participants except as required by law. This is outlined in the Qualtrics security statement and the Qualtrics privacy statement. Furthermore, the data that is stored on the Qualtrics server is de-identified (as stated above, participant contact details are only accessible to SSI).

For details on how SSI recruits participants, please see attachment of a new document, **'SSI recruitment details'**

Survey time

2. *The Committee considered that the survey may require longer than 20 minutes.*
a. Please consider piloting the survey before the project commences to obtain a more accurate estimation for the time required and accordingly revise the PIS with the amended time estimate.

To ensure a 20 minute completion time, questions related to outcome expectancies/risk perception and action control/self-regulatory effort have been removed from the baseline survey as they were not essential to the research question. Outcome expectancies/risk perception questions have been removed from the follow up survey. These revised questionnaires were piloted and took 15-20 minutes to complete (10 minutes for the follow-up questionnaire). Please see attached documents, **'smart snacking planning tool baseline_v2 tracked changes**' and '**smart snacking planning tool followup_v2 tracked changes**'

Administrative concerns

3. *Please revise the PIS to include the University of Sydney logo.*
4. Please revise the PIS to include the blank section to insert the HREC approval number.
The PIS now includes the University of Sydney logo and the HREC approval number. Please see attached **PIS easy v2 (tracked changes)**.

Other modifications

Sample size has been increased to 435 to accommodate a higher attrition rate from baseline to follow-up. This is based on advice about attrition rates obtained from the participant recruiter, SSI.

We have included **2 additional reminder emails,** such that there are no reminder emails at baseline, 1 week and 2 weeks. Previously there was only a reminder email at 1 week.

Planning tool and survey questions
Below is a summary of modifications to the planning tool and survey questions. Please refer to smart **snacking planning tool baseline_v2 (tracked changes)** for tracked changes. All relevant changes also apply to the follow-up tool (see **snacking planning tool followup_v2 (tracked changes)**.

- Changes to PIS as requested were incorporated into the planning tool
- Items for outcome expectancies/risk perception (baseline and follow up) and action control/self-regulatory effort (baseline) were deleted, for reasons outlined above
- Items for weight, height and language spoken at home were added
- Some text was simplified and more white space and images now included
- Some items were adjusted from a 4-point to a 7-point Likert scale
- The NVS health literacy measure was moved earlier in the baseline survey
- Some additional snack moments and solutions were incorporated into the volitional help sheet condition
- There are additional reminders and instructions to make a copy of the plan throughout the baseline survey
- The text on the final page of the follow up survey will also now provide a link to debriefing material.

THE UNIVERSITY OF SYDNEY

Study Information Sheet:
Smart snacking: An online planning tool

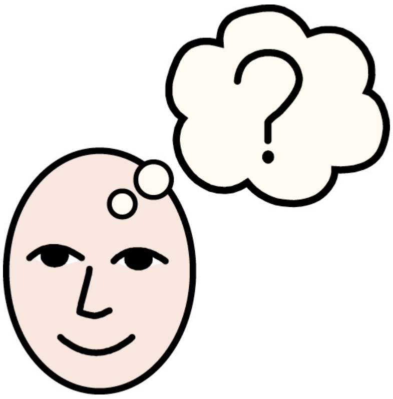

Hello. We are from the School of Public Health at the University of Sydney. Our names are

- Julie Ayre
- Carissa Bonner
- Kirsten McCaffery

We are doing a research study to find out more about tools to help people eat healthy snacks.

Snacks are important because they keep us going until the next meal. Sometimes though, we eat too many or choose snacks that are unhealthy. This can make us gain weight.

While many of us want to change the way we snack, this can be very hard to do. Often we make plans but have trouble sticking to them over long periods of time.
This study will look at online tools that help people stick to their plans.

We are asking you to be in our study because we are looking for people aged 30 years or more, who read and speak adequate English, and who would like to change the way they snack.

You can decide if you want to take part in the study or not. You don't have to - it's up to you.

This sheet tells you what we will ask you to do if you decide to take part in the study. Please read it carefully so that you can make up your mind about whether you want to take part.

You may stop completing the online survey at any point if you do not wish to continue, and we will not use your answers. You do not have to give a reason for not taking part. Once you have submitted your survey anonymously, your responses cannot be withdrawn.

If you have any questions, you can ask us or your family or someone else who looks after you. If you want to, you can call us any time on (02) 9351 7789.

What will happen if I say that I want to be in the study?

If you decide that you want to be in our study, we will ask you to do these things:

- Complete questions online about your demographics (for example, gender, age), the kinds of foods you eat and how you feel about your snacking behaviour
- Use the online planning tool to create a 'smart snacking' plan. You will receive a reminder message after 1 week.
- Try to follow the plan for one month, then complete some online questions about your snacking behaviour and your plan.

You can choose which questions you want to answer. If you don't want to give an answer, that's ok. You can stop answering questions at any time if you don't want to anymore.

This is an online study, so you can take part anywhere with access to the internet (smartphone or computer).

Will anyone else know what I say in the study?

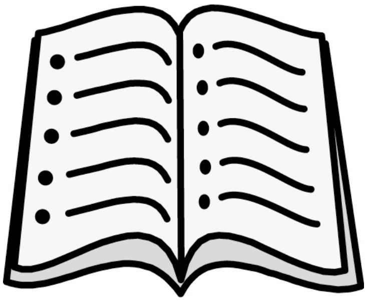

We won't tell anyone else what you say to us, except if you talk about someone hurting you or about you hurting yourself or someone else. Then we might need to tell someone to keep you and other people safe.

All of the information that we have about you from the study will be stored in a safe place and we will look after it very carefully. We will write a report about the study and show it to other people but we won't say your name in the report and no one will know that you were in the study, unless you tell us that it's ok for us to say your name.

How long will the study take?

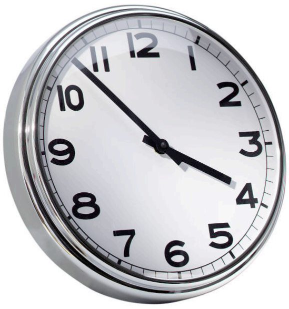

The first part of the study will take about 20 minutes to complete.

Over the following month you will be asked to try out your snacking plan.

The second part of the study will be sent to you after one month, and will take about 10 minutes to complete.

Are there any good things about being in the study?

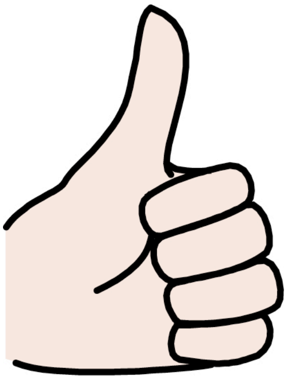

This study may help you think more about the way that you snack. This is the first step to changing your eating patterns. You may also find the tool useful for making these changes.

Are there any bad things about being in the study?

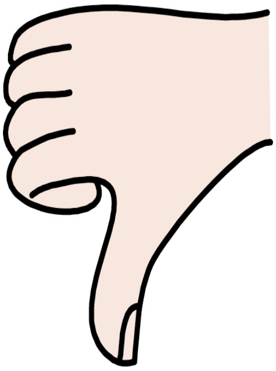

This study will take up some of your time, but we don't think it will be bad for you or cost you anything.

Will you tell me what you learnt in the study at the end?

Yes, we will if you want us to. There is a question on the next page that asks you if you want us to tell you what we learnt in the study. If you select Yes, when we finish the study we will tell you what we learnt.

What if I am not happy with the study or the people doing the study?

The ethical aspects of this study have been approved by the HREC of the University of Sydney [Project Number 2017/662].

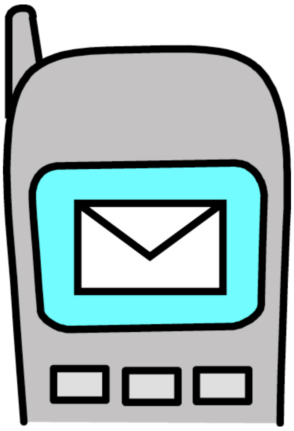

If you are not happy with how we are doing the study or how we treat you, then you or the person who looks after you can:

- **Call** the university on +61 2 8627 8176 or
- Write an **email** to human.ethics@sydney.edu.au

This sheet is for you to keep.

Study Information Sheet:
Smart snacking: An online planning tool

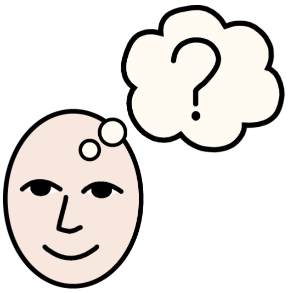

Hello. We are from the School of Public Health at the University of Sydney. Our names are

- Julie Ayre
- Carissa Bonner
- Kirsten McCaffery

We are doing a research study to find out more about tools to help people eat healthy snacks.

Snacks are important because they keep us going until the next meal. Sometimes though, we eat too many or choose snacks that are unhealthy. This can make us gain weight.

While many of us want to change the way we snack, this can be very hard to do. Often we make plans but have trouble sticking to them over long periods of time.
This study will look at online tools that help people stick to their plans.

We are asking you to be in our study because we are looking for people aged 30 years or more, who read and speak adequate English, and who would like to change the way they snack.

You can decide if you want to take part in the study or not. You don't have to - it's up to you.

This sheet tells you what we will ask you to do if you decide to take part in the study. Please read it carefully so that you can make up your mind about whether you want to take part.

You may stop completing the online survey at any point if you do not wish to continue, and we will not use your answers. You do not have to give a reason for not taking part. Once you have submitted your survey anonymously, your responses cannot be withdrawn.

If you have any questions, you can ask us or your family or someone else who looks after you. If you want to, you can call us any time on (02) 9351 7789.

What will happen if I say that I want to be in the study?

Formatted: Centered

Deleted: 1

Deleted: 21.06.17

If you decide that you want to be in our study, we will ask you to do these things:

- Complete questions online about your demographics (for example, gender, age), the kinds of foods you eat and how you feel about your snacking behaviour
- Use the online planning tool to create a 'smart snacking' plan. You will receive a reminder message after 1 week.
- Try to follow the plan for one month, then complete some online questions about your snacking behaviour and your plan.

You can choose which questions you want to answer. If you don't want to give an answer, that's ok. You can stop answering questions at any time if you don't want to anymore.

This is an online study, so you can take part anywhere with access to the internet (smartphone or computer).

Will anyone else know what I say in the study?

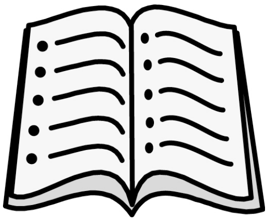

We won't tell anyone else what you say to us, except if you talk about someone hurting you or about you hurting yourself or someone else. Then we might need to tell someone to keep you and other people safe.

All of the information that we have about you from the study will be stored in a safe place and we will look after it very carefully. We will write a report about the study and show it to other people but we won't say your name in the report and no one will know that you were in the study, unless you tell us that it's ok for us to say your name.

How long will the study take?

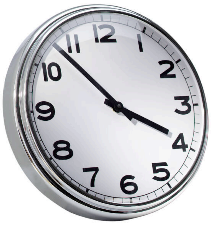

The first part of the study will take about 20 minutes to complete.

Over the following month you will be asked to try out your snacking plan.

The second part of the study will be sent to you after one month, and will take about 10 minutes to complete.

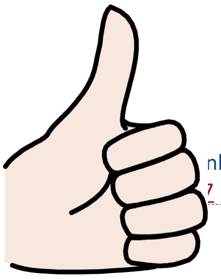

Are there any good things about being in the study?

Deleted: 1

Deleted: 21.06.17

This study may help you think more about the way that you snack. This is the first step to changing your eating patterns. You may also find the tool useful for making these changes.

Are there any bad things about being in the study?

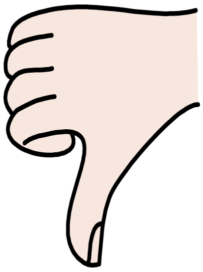

This study will take up some of your time, but we don't think it will be bad for you or cost you anything.

Will you tell me what you learnt in the study at the end?

Yes, we will if you want us to. There is a question on the next page that asks you if you want us to tell you what we learnt in the study. If you select Yes, when we finish the study we will tell you what we learnt.

What if I am not happy with the study or the people doing the study?

The ethical aspects of this study have been approved by the HREC of the University of Sydney [Project Number 2017/662].

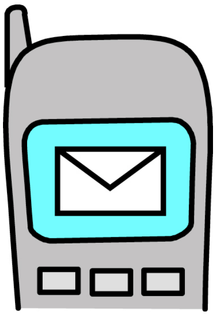

If you are not happy with how we are doing the study or how we treat you, then you or the person who looks after you can:

- **Call** the university on +61 2 8627 8176 or
- Write an **email** to human.ethics@sydney.edu.au

This sheet is for you to keep.

Deleted: 1

Deleted: 21.06.17

Screen 1 – Project Title Screen

Smart snacking: An online planning tool

(Baseline survey)

A study conducted by the School of Public Health at the University of Sydney

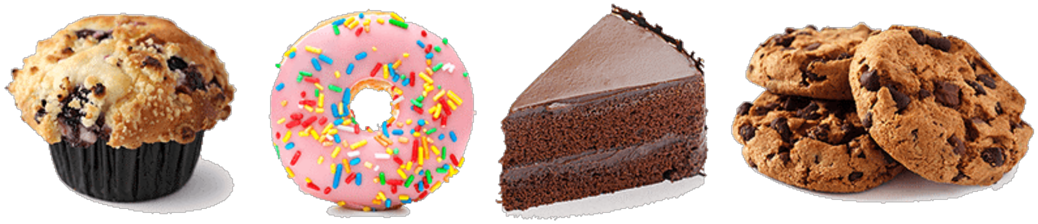

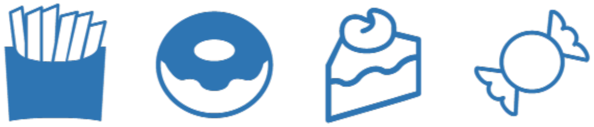

Smart Snacking

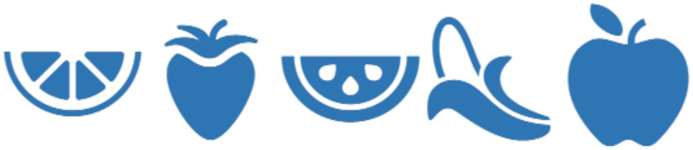

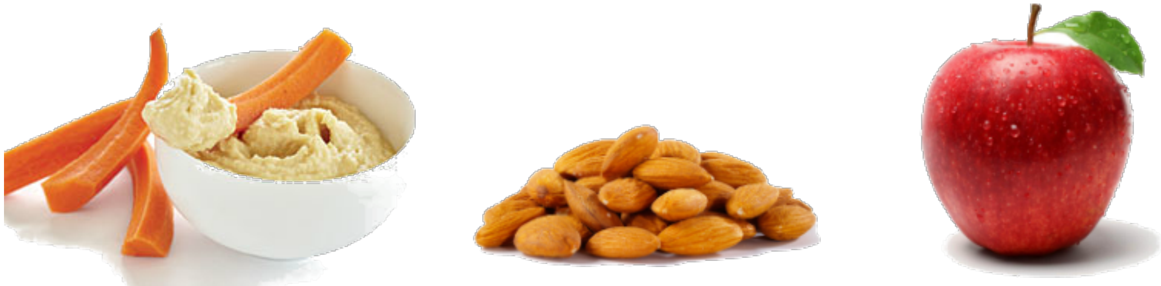

THE UNIVERSITY OF SYDNEY

Screen 1 – Participant information statement

Study Information Sheet:

Smart snacking: An online planning tool

Hello. We are from the School of Public Health at the University of Sydney. Our names are

- Julie Ayre
- Dr Carissa Bonner
- Prof Kirsten McCaffery

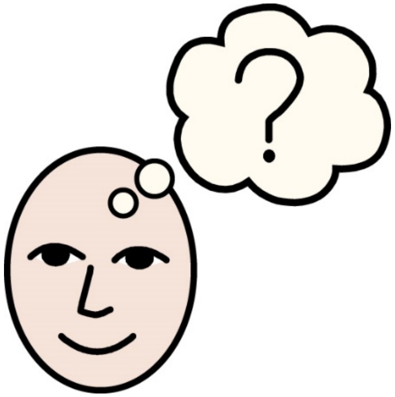

We are doing a research study to find out more about tools to help people eat healthy snacks.

Snacks are important because they keep us going until the next meal. Sometimes though, we eat too many or choose snacks that are unhealthy. This can make us gain weight.

While many of us want to change the way we snack, this can be very hard to do. Often we make plans but have trouble sticking to them over long periods of time.

This study will look at online tools that help people stick to their plans.

We are asking you to be in our study because we are looking for people aged 30 years or more, who read and speak adequate English, and who would like to change the way they snack.

You can decide if you want to take part in the study or not. You don't have to - it's up to you.

This sheet tells you what we will ask you to do if you decide to take part in the study. Please read it carefully so that you can make up your mind about whether you want to take part.

You may stop completing the online survey at any point if you do not wish to continue, and we will not use your answers. You do not have to give a reason for not taking part. Once you have submitted your survey anonymously, your responses cannot be withdrawn.

If you have any questions, you can ask us or your family or someone else who looks after you. If you want to, you can call us any time on (02) 9351 7789.

What will happen if I say that I want to be in the study?

If you decide that you want to be in our study, we will ask you to do these things:

- Complete questions online about your demographics (for example, gender, age), the kinds of foods you eat and how you feel about your snacking behaviour
- Use the online planning tool to create a 'smart snacking' plan. You will receive a reminder message after 1 week.
- Try to follow the plan for one month, then complete some online questions about your snacking behaviour and your plan.

You can choose which questions you want to answer. If you don't want to give an answer, that's ok. You can stop answering questions at any time if you don't want to anymore.

This is an online study, so you can take part anywhere with access to the internet (smartphone or computer).

Will anyone else know what I say in the study?

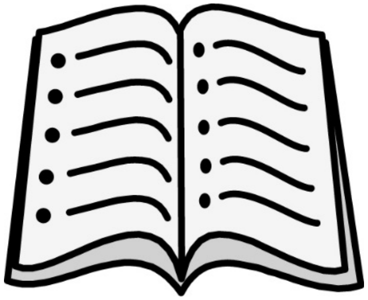

We won't tell anyone else what you say to us, except if you talk about someone hurting you or about you hurting yourself or someone else. Then we might need to tell someone to keep you and other people safe.

All of the information that we have about you from the study will be stored in a safe place and we will look after it very carefully. We will write a report about the study and show it to other people but we won't say your name in the report and no one will know that you were in the study, unless you tell us that it's ok for us to say your name.

How long will the study take?

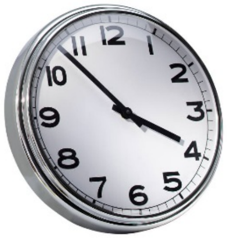

The first part of the study will take about 20 minutes to complete.

Over the following month you will be asked to try out your snacking plan.

The second part of the study will be sent to you after one month, and will take about 10 minutes to complete.

Are there any good things about being in the study?

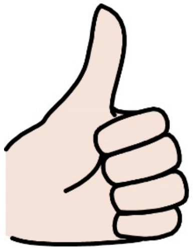

This study may help you think more about the way that you snack. This is the first step to changing your eating patterns. You may also find the tool useful for making these changes.

Are there any bad things about being in the study?

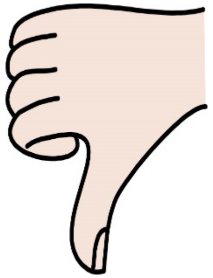

This study will take up some of your time, but we don't think it will be bad for you or cost you anything.

Will you tell me what you learnt in the study at the end?

Yes, we will if you want us to. There is a question on the next page that asks you if you want us to tell you what we learnt in the study. If you select Yes, when we finish the study we will tell you what we learnt.

What if I am not happy with the study or the people doing the study?

The ethical aspects of this study have been approved by the HREC of the University of Sydney [Project Number 2017/662].

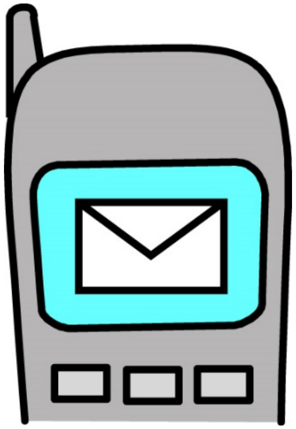

If you are not happy with how we are doing the study or how we treat you, then you or the person who looks after you can:

- **Call** the university on +61 2 8627 8176 or
- Write an **email** to human.ethics@sydney.edu.au

Screen 3 – Participant consent form

I give consent to my participation in the research project

TITLE: **Smart snacking: An online planning tool**

In giving my consent I acknowledge that:

1. The procedures required for the project and the time involved have been explained to me, and any questions I have about the project have been answered to my satisfaction.

2. I have read the Study Information Sheet and have been given the opportunity to discuss the information and my involvement in the project with the researcher/s.

3. I understand that being in this study is completely voluntary – I am not under any obligation to consent.

4. I understand that my involvement is strictly confidential. I understand that any research data gathered from the results of the study may be published however no information about me will be used in any way that is identifiable.

5. *I understand that I can withdraw from the study at any time, without affecting my treatment or my relationship with the researcher(s) or the University of Sydney now or in the future.*

6. I understand that I can stop my participation in this study at any time it I do not wish to continue and we will not use your answers.

7. *By completing the survey you have consented to be part of the study. Once you have submitted your survey anonymously, your responses cannot be withdrawn.*

I give my consent

Yes *No (if click no will not be directed to survey)*

Screen 4 – Baseline information and measures

Before we start please answer the following questions:

Age: [text box to type age]

Gender: [select gender from dropdown menu: Male/Female/Other]

English as first language: [select from dropdown menu: Yes/no], participant selects language from a list.

Highest level of Education: [Select from dropdown menu: Less than high school/high school/Certificate I/II / Certificate III/IV, Diploma, Bachelor degree or equivalent, Masters or Doctoral degree or equivalent]

Height: [participant selects unit of measurement and enters value into textbox]

Weight: [participant selects unit of measurement and enters value into textbox]

Smart snacking

Snacks are important because they keep us going until the next meal. Sometimes though, we eat too many, or choose snacks that are unhealthy.

Even though each snack is usually small, over time the snacks add up. This can make us gain weight.

Smart snacking means choosing nutritious, healthy snacks that give you energy until the next meal.

Which snacks are healthy?

Healthy snacks are low in kilojoules, fat, salt and sugars. These include fresh fruit, vegetables with dip, small amounts of dried fruit or nuts, yoghurt, coffee made with low fat milk, raisin toast, rice crackers and corn thins.

What are unhealthy snacks?

Unhealthy snacks are high in kilojoules, fat, salt and sugars. These include biscuits, cheese crackers, cakes, muffins, pastries, chocolate, lollies, potato chips, hot chips, French fries, some muesli bars and large coffees made with full cream milk.

Next

What are your snacking habits?

Before we get started on smart snacking, we'd like to know a little more about your snacking habits over the last month. For each question please answer by selecting a radio button.

Snacking habits

	Strongly disagree						Strongly agree
1. I eat unhealthy snacks frequently (all the time)	○	○	○	○	○	○	○
2. I eat unhealthy snacks automatically (without thinking)	○	○	○	○	○	○	○
3. I eat unhealthy snacks without having to consciously remember (When I eat unhealthy snacks I have not made an active decision to eat them)	○	○	○	○	○	○	○
4. I feel weird if I do not eat unhealthy snacks	○	○	○	○	○	○	○
5. I eat unhealthy snacks without thinking	○	○	○	○	○	○	○
6. It would require effort not to eat unhealthy snacks	○	○	○	○	○	○	○
7. Unhealthy snacks belong to (are part of) my routine (what I usually do)	○	○	○	○	○	○	○
8. I start eating unhealthy snacks before I realise I'm doing it	○	○	○	○	○	○	○
9. I would find it hard not to eat unhealthy snacks	○	○	○	○	○	○	○
10. I don't need to think about unhealthy snacks	○	○	○	○	○	○	○
11. Unhealthy snacking is typical (normal) for me	○	○	○	○	○	○	○
12. I've been eating unhealthy snacks for a long time	○	○	○	○	○	○	○

Snacks in the last week

Think about your snacking habits in the last week. To what extent have your snacks this week been (how true is it that your snacks this week were):

	Not at all						Very much
1. healthy (e.g. apple, banana, dried fruit)	O	O	O	O	O	O	O
2. unhealthy (e.g. chocolate, crisps, cake)	O	O	O	O	O	O	O

Yesterday's snacks

Which snacks did you eat yesterday? Do not include food eaten during breakfast, lunch or dinner.

- O Hot chips, potato gems or French fries
- O crisps or corn chips, crackers with cheese
- O Muffins, cake or doughnuts
- O biscuits
- O Pretzels
- O Pies, pasties or sausage rolls
- O Muesli bars, fruit bars, breakfast cereal bars
- O Chocolate
- O Lollies
- O Ice cream or ice blocks
- O Coffee with full cream milk
- O Coffee with skim milk
- O tea
- O Vita weat or Ryvita
- O Raisin toast
- O Apple or pear
- O Banana, mango
- O Orange or grapefruit
- O Kiwi fruit, mandarins
- O Yoghurt
- O Cherries, peaches or plums
- O Grapes or berries
- O Watermelon, melon
- O Carrot, cucumber or capsicum
- O Other fruit
- O Dip (e.g. hommos), cottage cheese or peanut butter
- O Nuts
- O Popcorn, rice crackers or corn thins
- O yoghurt
- O Other

Snack diary

1. How often do you usually eat oven baked potato gems/chips/hashbrowns, hot chips/French fries, wedges or fried potatoes?

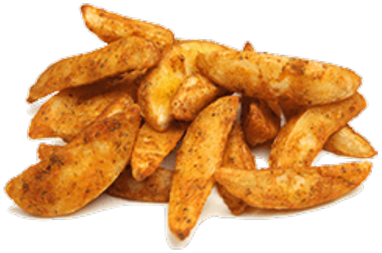
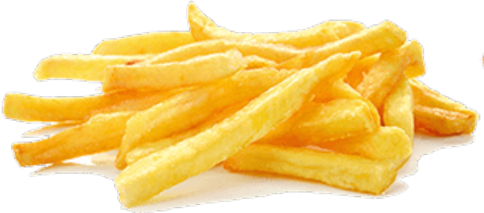
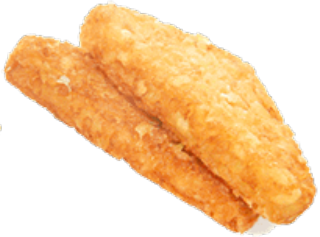

○ each week
○ each day
○ each month
○ I don't eat this

In total, how many serves of potato gems/chips/hashbrowns, hot chips/French fries, wedges or fried potatoes do you usually eat in the timeframe selected above?

1 serve =
12 fried hot chips
1 cup (60g) potato gems/hashbrowns, or wedges

[slider for answer]

2. How often do you usually eat savoury snacks such as crisps, pretzels or plain/flavoured crackers?

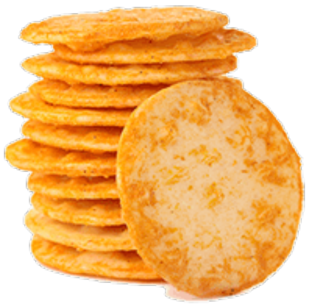 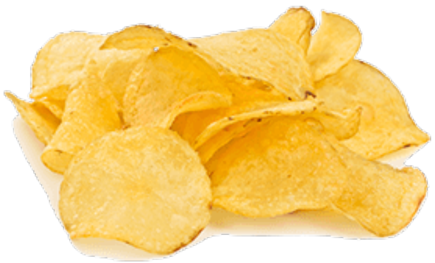 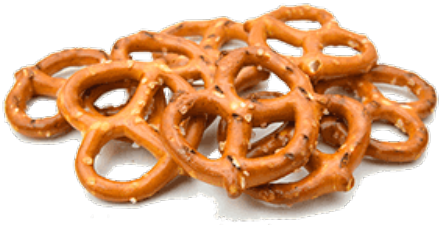

○ each week
○ each day
○ each month
○ I don’t eat this

In total, how many serves of savoury snacks such as crisps, pretzels or plain/flavoured crackers do you usually eat in the timeframe selected above?

1 serve =
½ snack size packet of crisps
1 handful (30g) of salty crackers or pretzels

[slider for answer]

3. How often do you usually have sweet biscuits/cakes/ buns/ muffins/ doughnuts? Include both home-made and bought.

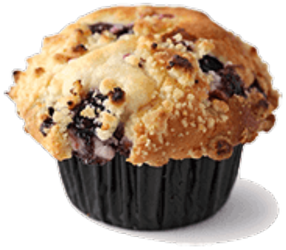 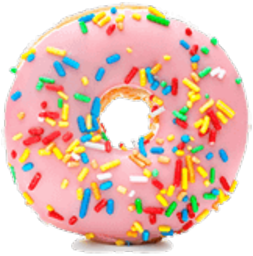 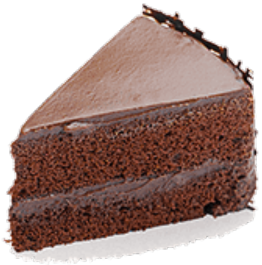 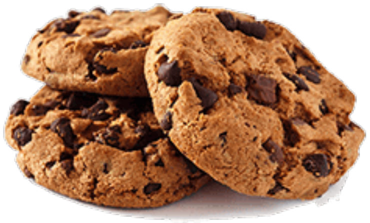

○ each week
○ each day
○ each month
○ I don’t eat this

In total, how many serves of sweet biscuits/cakes/buns/muffins/doughnuts do you usually eat in the timeframe selected above?

1 serve =
2-3 (35g) sweet biscuits
1 doughnut
1 slice (40g) of plain cake or sweet bun
1 small muffin

[slider for answer]

4. How often do you usually eat savoury pastries?

This includes pies, pasties, sausage rolls, Kransky Dogs and frankfurters wrapped in pastry.

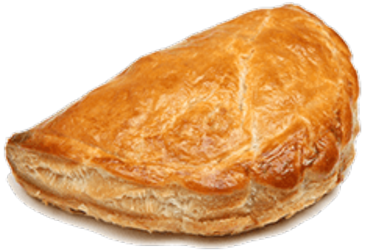 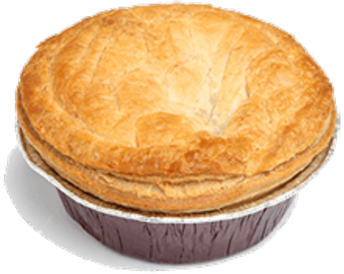 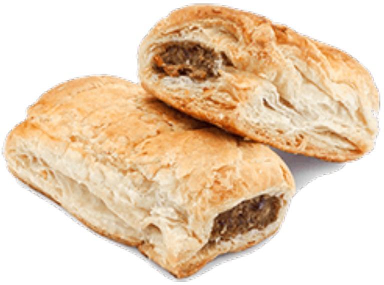

○ each week
○ each day
○ each month
○ I don’t eat this

In total, how many serves of pies or savoury pastries do you usually eat in the timeframe selected above?

1 serve =
1/4 (60g) commerical meat pies or pastie
1 party size pie or sausage roll

[slider for answer]

5. How often do you usually eat snack type bars?

This includes muesli bars, fruit bars and breakfast cereal bars.

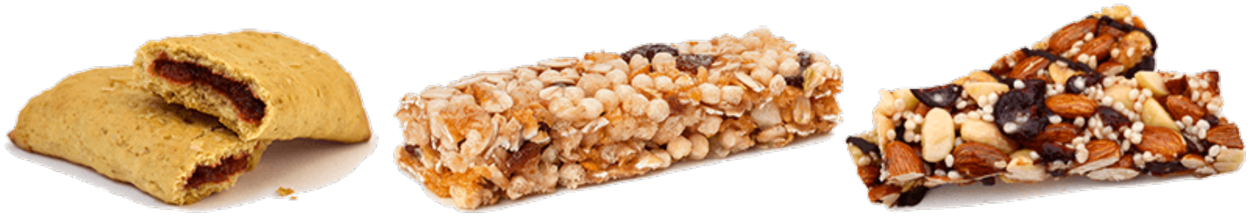

O each week
O each day
O each month
O I don’t eat this

In total, how many snack type bars do you usually eat in the timeframe selected above? This includes muesli bars, fruit bars and breakfast cereal bars.

[slider for answer]

6. How often do you usually have chocolate or lollies? *This question is required.
Include all types of chocolate and both hard and soft lollies.

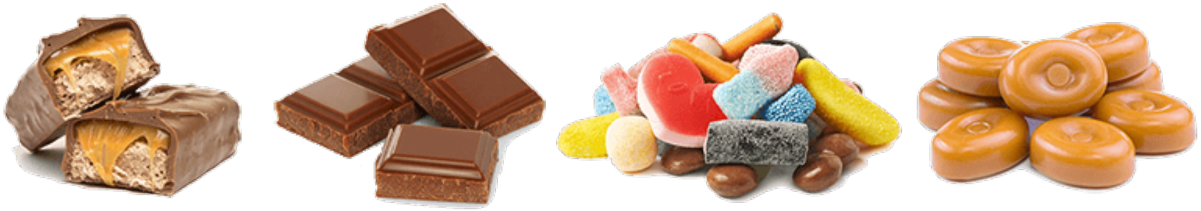

○ each week
○ each day
○ each month
○ I don't eat this

In total, how many serves of chocolate or lollies do you usually eat in the timeframe selected above?

1 serve =
½ chocolate bar
4 pieces of chocolate (25g)
5-6 (40g) lollies

[slider for answer]

7. How often do you usually have ice-cream or ice-blocks?

This includes ice-blocks, ice-cream in a bowl or ice-creams on a stick.

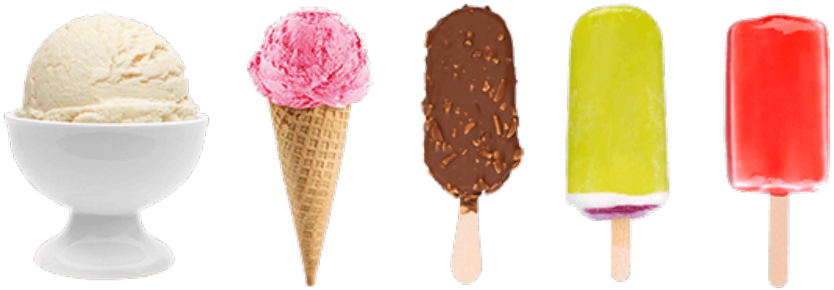

○ each week
○ each day

○ each month
○ I don't eat this

In total, how many serves of ice-cream or ice-blocks do you usually eat in the timeframe selected above? *This question is required.

1 serve =
2 scoops (60g) ice-cream
1 stick ice-cream or ice-block

[slider for answer]

What do you think about snacking?

**Over the next month:**	Strongly disagree						Strongly agree
7. I want to eat fewer unhealthy snacks	○	○	○	○	○	○	○
8. I plan to eat fewer unhealthy snacks	○	○	○	○	○	○	○
9. I intend (plan) to eat fewer unhealthy snacks	○	○	○	○	○	○	○

What snacking strategies do you already have?

**I already have clear plans about…**	Strongly disagree						Strongly agree
1. How I will change my unhealthy snacking habits	○	○	○	○	○	○	○
2. When I will change my unhealthy snacking habits	○	○	○	○	○	○	○
3. When I need to watch out so that I keep choosing healthy snacks	○	○	○	○	○	○	○
4. What to do in situations that make it hard to avoid unhealthy snacks (times when it is hard not to eat unhealthy snacks)	○	○	○	○	○	○	○
5. How to get back on track when I have eaten unhealthy snacks	○	○	○	○	○	○	○

How do you feel about changing your unhealthy snacking?

I am sure that…	Strongly disagree						Strongly agree
1. I can avoid eating unhealthy snacks for the next month	○	○	○	○	○	○	○
**I am certain that I can avoid eating unhealthy snacks even if…**							
2. Friends or family are eating unhealthy snacks	○	○	○	○	○	○	○
3. I am bored	○	○	○	○	○	○	○
4. I am craving an unhealthy snack	○	○	○	○	○	○	○

Nobody is perfect. Sometimes we have trouble sticking to our plans. Imagine you have started eating unhealthy snacks again. How confident are you about changing this habit?

I am certain I could go back to eating healthy snacks…							
5. Even after I ate 1 unhealthy snack	○	○	○	○	○	○	○
6. Even after a few days of eating unhealthy snacks	○	○	○	○	○	○	○
7. Even after a week of eating unhealthy snacks	○	○	○	○	○	○	○

Please read the nutritional panel below to answer the following questions. The panel is information on the back of a container or ice cream.

**Nutrition Facts**	
Serving Size	1/2 cup
Servings per container	4
Amount per serving	
Calories 250	Fat Cal 120
	%DV
**Total Fat** 13g	20%
Sat Fat 9g	40%
**Cholesterol** 28mg	12%
**Sodium** 55mg	2%
**Total Carbohydrate** 30g	12%
Dietary Fiber 2g	
Sugars 23g	
**Protein** 4g	8%

* Percent Daily Values (DV) are based on a 2,000 calorie diet. Your daily values may be higher or lower depending on your calorie needs.

Ingredients: Cream, Skim Milk, Liquid Sugar, Water, Egg Yolks, Brown Sugar, Milkfat, Peanut Oil, Sugar, Butter, Salt, Carrageenan, Vanilla Extract.

1. If you eat the entire container, how many calories will you eat?

2. If you are allowed to eat 60 grams of carbohydrates as a snack, how much ice cream could you have?

3. Your doctor advises you to reduce the amount of saturated fat in your diet. You usually have 42g of saturated fat each day, which includes one serving of ice cream. If you stop eating ice cream, how many grams of saturated fat would you be consuming each day?

4. If you usually eat 2,500 calories in a day, what percentage of your daily value of calories will you be eating if you eat one serving?

Pretend that you are allergic to the following substances: penicillin, peanuts, latex gloves, and bee stings.

5. Is it safe for you to eat this ice cream?
6. *[Ask only if patient responds 'no' to question 5]*: Why not?

Great! It looks like you’re ready to **snack smarter**! The next step is to come up with a plan.

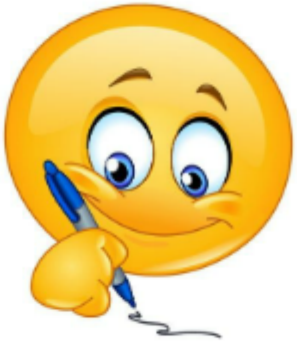

Let’s get started!

The next section depends on intervention condition.

Screen 4a – Volitional help sheet condition

Smart snacking

Step 1: Snack moments

Sometimes we snack because we are hungry, but there are lots of other reasons too.

Think about your snacks in the **last week**. Below is a list of '**snack moments**.' These are times when people tend to choose unhealthy snacks or eat too much.

Choose **3** snack moments from the list that happened to you the **most often** in the last week.

I often eat unhealthy snacks when…

The snack is in front of me	I have a craving	I am bored	I am tired	I start with one piece but then keep eating	I am in front of a TV or computer
Someone offers me the snack	People around me are eating	I am happy	I am drinking alcohol	I am busy or stressed	I am about to go to bed
I am drinking tea or coffee	I am sad	I want to reward myself	it is part of a celebration or special event	it is my day off	I have arrived home

Next

Step 2: The ‘key’ snack [illegible]

Below are your top 3 snack moments.

Some snack moments will be more important than others. Choose the **1** that you would be **happiest** to change.

I really want to change my habit of snacking when…

I am bored

I am sitting in front of a TV or computer

people around me are eating

Next

Step 3: Make a plan

Great! Your key snack moment is:
Eating unhealthy snacks when **I am bored [example text]**.

The last step is to come up with a plan! **Choose the solution that you think will work best for you**. Drag it into the space on the right

I will go outside for a walk	I will listen to music	I will chat to someone for 5 minutes
I will drink tea	I will do a chore or task	I will eat a smaller amount
I will drink a large glass of water	I will eat a piece of fruit	I will take the food out of the packet and put it on a plate
	I will eat fresh vegetables and dip	

If I want a snack because I am bored, I will…

Next

Step 4: Your plan is almost ready!

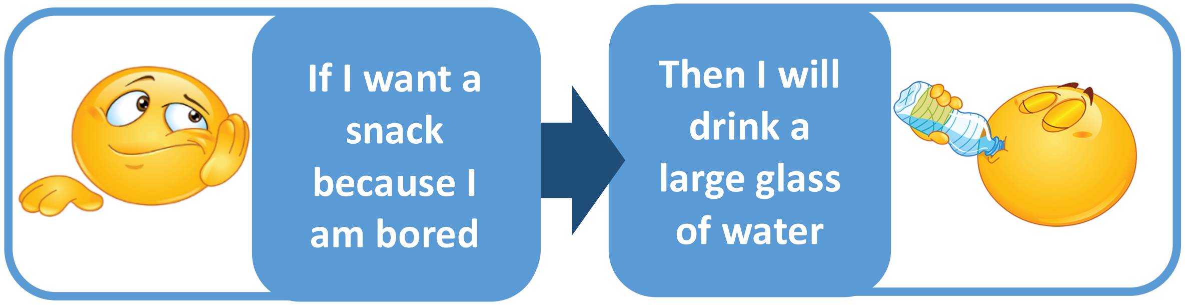

Imagine how your plan might feel. **When do you feel bored?**

Sometimes we want unhealthy snacks when we :

- waiting for a friend to arrive
- on a long train or bus trip
- watching TV
- doing long repetitive tasks

If this happens, and you want an unhealthy snack, do you think you could drink a large glass of water instead?

How hard do you think it will be to do this plan for the next month?

Very easy | Very hard

[If score for previous question is <7/10]

Your score shows that this plan may be hard for you.

Let's make a plan that is a bit easier!

I will go outside for a walk	I will listen to music	I will chat to someone for 5 minutes
I will drink tea	I will do a chore or task	I will eat a smaller amount
I will drink a large glass of water	I will eat a piece of fruit	I will take the food out of the packet and put it on a plate
	I will eat fresh vegetables and dip	

If I want a snack because I am bored, I will...

[If score for previous question is ≥7/10]

Step 4: Your plan is almost ready!

Your smart snacking plan

Well done! Try to remember this plan for the **next month**. Say it **3** times to yourself. You can also make a copy of your plan at the end of this survey.

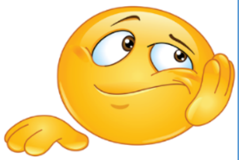

If I want a snack because I am bored

Then I will drink a large glass of water

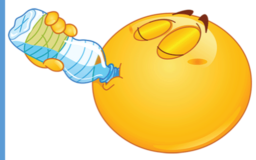

Take a screenshot

Next

Next

Screen 4b – Detailed planning condition (active control)

Smart snacking

Your healthy snacking plan

We want you to plan how you will change your unhealthy snacking behaviour each day because forming plans has been shown to improve snacking habits.

You are free to choose how you do this but we want you to formulate your plans in as much detail as possible.

Please pay attention to the **situations** in which you will implement (carry out) these plans. Focus on situations when you are not hungry but find yourself snacking.

Please enter your plan below
[Text box – e.g. When I am bored and hungry I will remember to drink a large glass of water first.]

How hard do you think it will be to do this plan for the next month?

Very easy Very hard

Next

Your healthy snacking plan

Well done! Try to remember this plan for the **next month**. Say it a few times to yourself. You may also like to write the plan down or take a screenshot. You can also download this plan as a PDF at the end of the survey.

[Example: When I am bored and hungry I will remember to drink a large glass of water first.]

Take a screenshot

Next

Screen 4c – Helpful hints sheet (passive control)

Smart snacking

Read the 'healthy snacks' fact sheet below.

Healthy snacks

Eating the right balance of healthy foods can help you live well. Snacks can help you meet your daily nutrition needs, but it's important to make healthy choices and watch your portions to manage your weight.

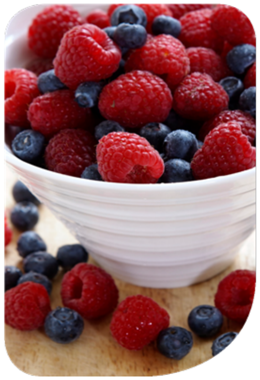

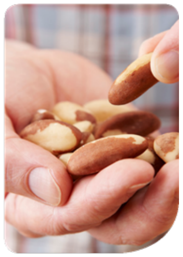

The snacks below are good choices. Try these in the recommended portions.

Fruit

» Fresh fruit: one apple/pear/orange/ large peach/large nectarine/small banana; three apricots/mandarins; two kiwifruit/plums; one cup of grapes/ cherries/melon
» Canned fruit in natural juice (drained): one cup of fruit salad/apricots/peaches
» Dried fruit: one tablespoon of sultanas; six prunes; four whole dried apricots

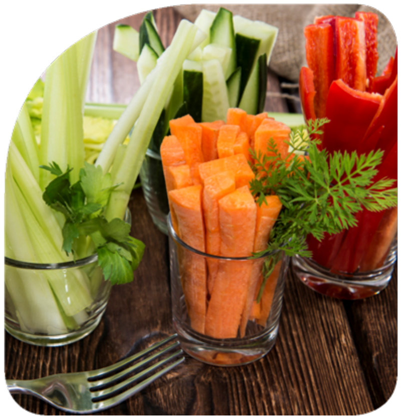

Vegetables

» ½ cup no-added-salt baked beans
» One steamed cob of corn
» Small can (125g) corn kernels
» Vegetable sticks: celery, carrot, capsicum, cucumber or snow peas with 1 slice (20g) of low-fat cheese or one tablespoon of hummus, tomato salsa or tzatziki yoghurt dip
» Celery boats with two tablespoons of low-fat cream cheese or no-added-salt peanut butter
» A handful of cherry tomatoes

Dairy and alternatives

» 100g of low-fat flavoured yoghurt or 200g of diet/natural/plain varieties. Flavour natural yoghurt with passionfruit, fresh/frozen berries, one teaspoon of honey, nuts or seeds
» 250ml of low-fat plain milk/soy milk with added calcium
» Medium cappuccino/café latte/flat white coffee with low-fat milk/soy milk

Breads and cereals

» One slice of dense grainy/seeded bread (plain or toasted) with a thin spread of margarine or avocado or low-fat ricotta or cottage cheese or hummus or chutney, with sliced tomato and sprouts
» One thin slice of raisin/wholegrain fruit bread, fresh or toasted, with a thin spread of margarine or no-added-salt peanut butter/natural nut spread or 100% fruit jam
» Half a wholegrain English muffin grilled with one slice of low-fat cheese and a sliced tomato
» One small wholemeal pita pocket or wrap with grated carrot and grated low-fat cheese
» Four rice/corn thins spread with a ¼ of an avocado and topped with sliced cucumber and tinned salmon
» Ten plain wholegrain rice crackers with low-fat cream cheese or hummus or salsa
» Two wholegrain crispbread with cottage cheese or avocado and tomato

Nuts and seeds

» 30 grams (a small handful) of plain, raw unsalted nuts: mixed, macadamias, walnuts, almonds, cashews, brazil, hazelnuts, pecans, pistachios
» 30 grams (a small handful) of pumpkin or sunflower seeds

Lean meat and alternatives

» A mini can of tuna/salmon/sardines served in half a capsicum or a lettuce cup
» A hard-boiled egg

Grains

» One cup of air-popped popcorn served plain, or sprinkled with cinnamon/ paprika/chilli

Muesli and nut bars

Muesli and nut bars are a 'sometimes' snack as they can be high in kilojoules, added fat and sugar. Check the nutrition information panel to choose higher fibre/ healthier options and eat these only occasionally.

Homemade snacks

Homemade snacks can be a healthy choice but they can still be high in kilojoules. Eat small portions of these only occasionally.
» Cakes, biscuits, muffins and slices baked at home can be made healthier by using olive, canola, rice bran, grapeseed or sunflower margarines or oils; less sugar, wholemeal flour, oats, nuts, seeds and fruit.

When shopping, try to limit buying foods that are high in saturated fat, sugar, salt (sodium), such as:

- sweet biscuits and some crackers
- cakes, muffins and slices,
- pastries
- chocolates and confectionary
- potato crisps, corn chips and other salty snacks
- processed and pre-packaged snack foods
- fast foods and high fat takeaways

Drinks

It's important to stay hydrated throughout the day. Water is the best drink, but some other alternatives include:
» mineral or soda water flavoured with sliced lemon/lime/frozen berries/ cucumber/fresh mint
» black/oolong/green/herbal tea
» coffee or decaffeinated coffee with a dash of skim or low-fat milk.
As an occasional substitute, diet cordial or diet soft drink may add variety without extra sugar or kilojoules.

What is your plan? How hard do you think it will be to do this plan for the next month?

Very easy — Very hard

Next

Screen 5 – Tool evaluation

Great work! Now you have a plan to try out for the next month.

We would like to ask a few more questions before you finish.

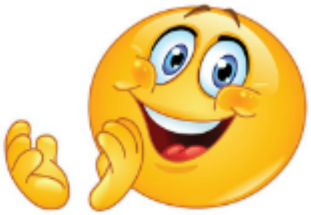

How hard was the tool to use?

Not at all hard	A little hard	Somewhat hard	Very hard	Extremely hard
○	○	○	○	○

Screen 6 – Health literacy measures

1. How confident are you filling out medical forms by yourself?

Not at all	A little bit	Somewhat	Quite a bit	Extremely
○	○	○	○	○

On a scale from very difficult to very easy, how easy would you say it is to:

	Very difficult	Fairly difficult	Fairly easy	Very easy	Don't know
1. Judge when you may need to get a second opinion from another doctor?	○	○	○	○	○
2. Use information the doctor gives you to make decisions about your illness?	○	○	○	○	○
3. Find information on how to manage mental health problems like stress or depression?	○	○	○	○	○
4. Judge if the information on health risks in the media is reliable?	○	○	○	○	○
5. Find out about activities that are good for your mental well-being?	○	○	○	○	○
6. Understand information in the media on how to get healthier?	○	○	○	○	○

That's it! Good luck with your smart snacking plan!

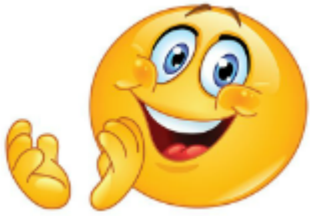

Before you leave, would you like to print or download a copy of your plan?

Print or download plan
to download a PDF select 'print as PDF' when you select the printer.

[space below will show a copy of the plan OR a copy of the tips sheet]

Next

Before we finish, it is very important that you have a copy of your plan!

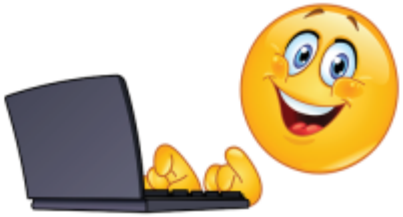

You can write it down, print it out or save it as a PDF or screenshot.

Press the 'back' button if you need to make a copy. If you have a copy, click 'yes' below.

Yes, I have a copy of my plan!

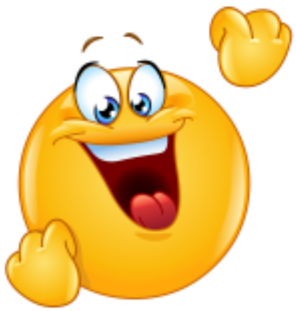

That's it! Good luck with your smart snacking plan!

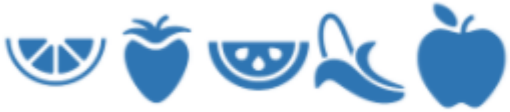

Thank you for completing this survey.

We will check in with you in **one month** to see how your plan is going.

Screen 1 – Project Title Screen

Smart snacking: An online planning tool
(Baseline survey)

THE UNIVERSITY OF SYDNEY

A study conducted by the School of Public Health at the University of Sydney

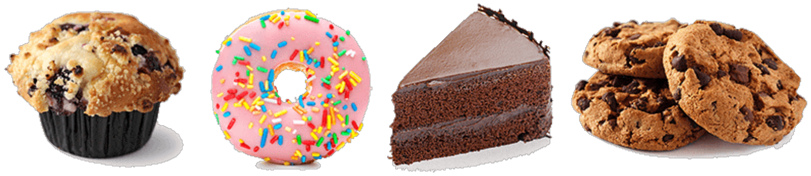

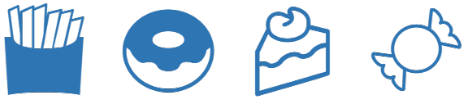

Smart Snacking

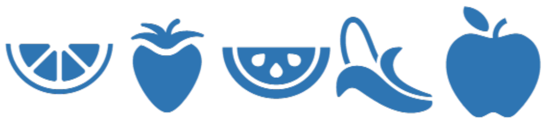

Deleted:

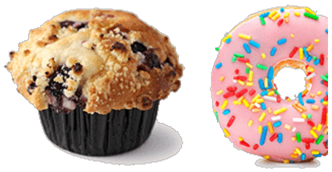

Moved down [1]:

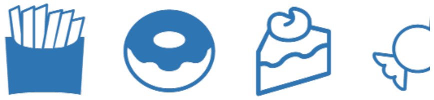

Smart Snacking

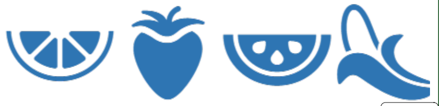

... [1]

Formatted: Font:(Default) Arial, 13.5 pt, Font color: R,G,B (67,70,77)

Formatted: Font:(Default) Arial, 13.5 pt, Font color: R,G,B (67,70,77)

Formatted: Font:(Default) Arial, 13.5 pt, Font color: R,G,B (67,70,77)

Formatted: Font:(Default) Arial, 13.5 pt, Font color: R,G,B (67,70,77)

Moved (insertion) [1]

Formatted: Font:(Default) Arial, 13.5 pt, Font color: R,G,B (67,70,77)

Formatted: Font:(Default) Arial, 13.5 pt, Font color: R,G,B (67,70,77)

Formatted: Font:(Default) Arial, 13.5 pt, Font color: R,G,B (67,70,77)

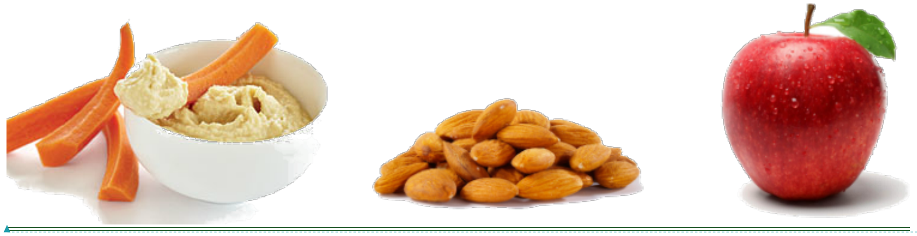

THE UNIVERSITY OF SYDNEY

Formatted: Font:(Default) Arial, 13.5 pt, Font color: R,G,B (67,70,77)

Formatted: Font:(Default) Arial, Bold

Formatted: Centered

Screen 1 – Participant information statement

Formatted: Font:(Default) Arial, Bold

THE UNIVERSITY OF SYDNEY

Study Information Sheet:

Smart snacking: An online planning tool

Hello. We are from the School of Public Health at the University of Sydney. Our names are

- Julie Ayre
- Dr Carissa Bonner
- Prof Kirsten McCaffery

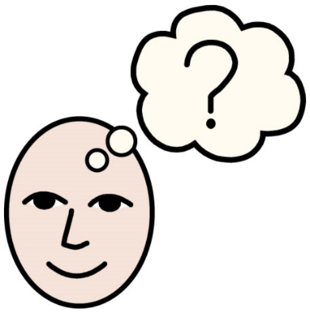

We are doing a research study to find out more about tools to help people eat healthy snacks.

Snacks are important because they keep us going until the next meal. Sometimes though, we eat too many or choose snacks that are unhealthy. This can make us gain weight.

While many of us want to change the way we snack, this can be very hard to do. Often we make plans but have trouble sticking to them over long periods of time.

This study will look at online tools that help people stick to their plans.

We are asking you to be in our study because we are looking for people aged 30 years or more, who read and speak adequate English, and who would like to change the way they snack.

You can decide if you want to take part in the study or not. You don't have to - it's up to you.

This sheet tells you what we will ask you to do if you decide to take part in the study. Please read it carefully so that you can make up your mind about whether you want to take part.

You may stop completing the online survey at any point if you do not wish to continue, and we will not use your answers. You do not have to give a reason for not taking part. Once you have submitted your survey anonymously, your responses cannot be withdrawn.

If you have any questions, you can ask us or your family or someone else who looks after you. If you want to, you can call us any time on (02) 9351 7789.

What will happen if I say that I want to be in the study?

If you decide that you want to be in our study, we will ask you to do these things:

- Complete questions online about your demographics (for example, gender, age), the kinds of foods you eat and how you feel about your snacking behaviour
- Use the online planning tool to create a 'smart snacking' plan. You will receive a reminder message after 1 week.
- Try to follow the plan for one month, then complete some online questions about your snacking behaviour and your plan.

You can choose which questions you want to answer. If you don't want to give an answer, that's ok. You can stop answering questions at any time if you don't want to anymore.

This is an online study, so you can take part anywhere with access to the internet (smartphone or computer).

Will anyone else know what I say in the study?

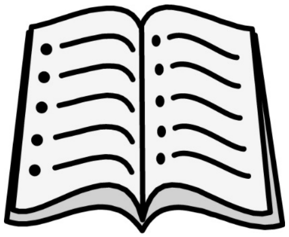

We won't tell anyone else what you say to us, except if you talk about someone hurting you or about you hurting yourself or someone else. Then we might need to tell someone to keep you and other people safe.

All of the information that we have about you from the study will be stored in a safe place and we will look after it very carefully. We will write a report about the study and show it to other people but we won't say your name in the report and no one will know that you were in the study, unless you tell us that it's ok for us to say your name.

How long will the study take?

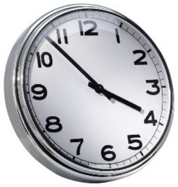

The first part of the study will take about 20 minutes to complete.

Over the following month you will be asked to try out your snacking plan.

The second part of the study will be sent to you after one month, and will take about 10 minutes to complete.

Are there any good things about being in the study?

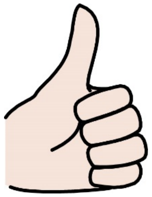

This study may help you think more about the way that you snack. This is the first step to changing your eating patterns. You may also find the tool useful for making these changes.

Are there any bad things about being in the study?

This study will take up some of your time, but we don't think it will be bad for you or cost you anything.

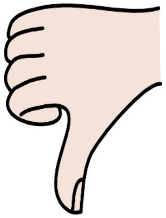

Will you tell me what you learnt in the study at the end?

Yes, we will if you want us to. There is a question on the next page that asks you if you want us to tell you what we learnt in the study. If you select Yes, when we finish the study we will tell you what we learnt.

What if I am not happy with the study or the people doing the study?

The ethical aspects of this study have been approved by the HREC of the University of Sydney [Project Number 2017/662].

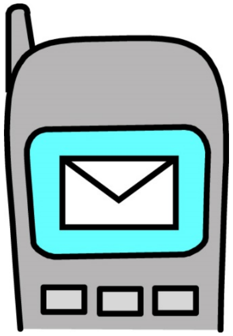

If you are not happy with how we are doing the study or how we treat you, then you or the person who looks after you can:

- **Call** the university on +61 2 8627 8176 or
- Write an **email** to human.ethics@sydney.edu.au

Screen 3 – Participant consent form

I give consent to my participation in the research project

TITLE: **Smart snacking: An online planning tool**

In giving my consent I acknowledge that:

1. The procedures required for the project and the time involved have been explained to me, and any questions I have about the project have been answered to my satisfaction.
2. I have read the Study Information Sheet and have been given the opportunity to discuss the information and my involvement in the project with the researcher/s.
3. I understand that being in this study is completely voluntary – I am not under any obligation to consent.
4. I understand that my involvement is strictly confidential. I understand that any research data gathered from the results of the study may be published however no information about me will be used in any way that is identifiable.
5. *I understand that I can withdraw from the study at any time, without affecting my treatment or my relationship with the researcher(s) or the University of Sydney now or in the future.*
6. I understand that I can stop my participation in this study at any time it I do not wish to continue and we will not use your answers.
7. *By completing the survey you have consented to be part of the study. Once you have submitted your survey anonymously, your responses cannot be withdrawn.*

I give my consent

Yes *No (if click no will not be directed to survey)*

Screen 4 – Baseline information and measures

Before we start please answer the following questions:

Age: [text box to type age]

Gender: [select gender from dropdown menu: Male/Female/Other]

English as first language: [select from dropdown menu: Yes/no], participant selects language from a list.

Highest level of Education: [Select from dropdown menu: Less than high school/high school/Certificate I/II / Certificate III/IV, Diploma, Bachelor degree or equivalent, Masters or Doctoral degree or equivalent]

Height: [participant selects unit of measurement and enters value into textbox]

Weight: [participant selects unit of measurement and enters value into textbox]

Deleted: Select years from dropdown menu: 30/31/32/33 etc...

Formatted: Font:Not Bold

Smart snacking

Snacks are important because they keep us going until the next meal. Sometimes though, we eat too many, or choose snacks that are unhealthy.

Even though each snack is usually small, over time the snacks add up. This can make us gain weight.

Smart snacking means choosing nutritious, healthy snacks that give you energy until the next meal.

Which snacks are healthy?

Healthy snacks are low in kilojoules, fat, salt and sugars. These include fresh fruit, vegetables with dip, small amounts of dried fruit or nuts, yoghurt, coffee made with low fat milk, raisin toast, rice crackers and corn thins.

What are unhealthy snacks?

Unhealthy snacks are high in kilojoules, fat, salt and sugars. These include biscuits, cheese crackers, cakes, muffins, pastries, chocolate, lollies, potato chips, hot chips, French fries, some muesli bars and large coffees made with full cream milk.

Next

What are your snacking habits?

Before we get started on smart snacking, we'd like to know a little more about your snacking habits over the last month. For each question please answer by selecting a radio button.

Snacking habits

	Strongly disagree						Strongly agree
1. I eat unhealthy snacks frequently (all the time)	○	○	○	○	○	○	○
2. I eat unhealthy snacks automatically (without thinking)	○	○	○	○	○	○	○
3. I eat unhealthy snacks without having to consciously remember (When I eat unhealthy snacks I have not made an active decision to eat them)	○	○	○	○	○	○	○
4. I feel weird if I do not eat unhealthy snacks	○	○	○	○	○	○	○
5. I eat unhealthy snacks without thinking	○	○	○	○	○	○	○
6. It would require effort not to eat unhealthy snacks	○	○	○	○	○	○	○
7. Unhealthy snacks belong to (are part of) my routine (what I usually do)	○	○	○	○	○	○	○
8. I start eating unhealthy snacks before I realise I'm doing it	○	○	○	○	○	○	○
9. I would find it hard not to eat unhealthy snacks	○	○	○	○	○	○	○
10. I don't need to think about unhealthy snacks	○	○	○	○	○	○	○
11. Unhealthy snacking is typical (normal) for me	○	○	○	○	○	○	○
12. I've been eating unhealthy snacks for a long time	○	○	○	○	○	○	○

Deleted: (daily, weekly, monthly)

Snacks in the last week

Think about your snacking habits in the last week. To what extent have your snacks this week been (how true is it that your snacks this week were):

	Not at all						Very much
1. healthy (e.g. apple, banana, dried fruit)	O	O	O	O	O	O	O
2. unhealthy (e.g. chocolate, crisps, cake)	O	O	O	O	O	O	O

Yesterday's snacks

Which snacks did you eat yesterday? Do not include food eaten during breakfast, lunch or dinner.

- O Hot chips, potato gems or French fries
- O crisps or corn chips, crackers with cheese
- O Muffins, cake or doughnuts
- O biscuits
- O Pretzels
- O Pies, pasties or sausage rolls
- O Muesli bars, fruit bars, breakfast cereal bars
- O Chocolate
- O Lollies
- O Ice cream or ice blocks
- O Coffee with full cream milk
- O Coffee with skim milk
- O tea
- O Vita weat or Ryvita
- O Raisin toast
- O Apple or pear
- O Banana, mango
- O Orange or grapefruit
- O Kiwi fruit, mandarins
- O Yoghurt
- O Cherries, peaches or plums
- O Grapes or berries
- O Watermelon, melon
- O Carrot, cucumber or capsicum
- O Other fruit
- O Dip (e.g. hommos), cottage cheese or peanut butter
- O Nuts
- O Popcorn, rice crackers or corn thins
- O yoghurt
- O Other

Deleted: Crackers,

Deleted: Snacks in the last week ... [2]

Snack diary

Deleted: for previous week

1. How often do you usually eat oven baked potato gems/chips/hashbrowns, hot chips/French fries, wedges or fried potatoes?

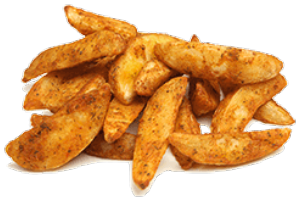 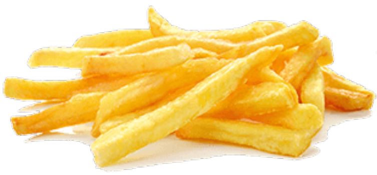 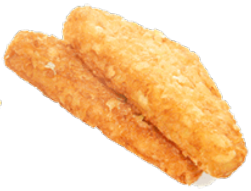

○ each week
○ each day
○ each month
○ I don't eat this

In total, how many serves of potato gems/chips/hashbrowns, hot chips/French fries, wedges or fried potatoes do you usually eat in the timeframe selected above?

1 serve =
12 fried hot chips
1 cup (60g) potato gems/hashbrowns, or wedges

[slider for answer]

2. How often do you usually eat savoury snacks such as crisps, pretzels or plain/flavoured crackers?

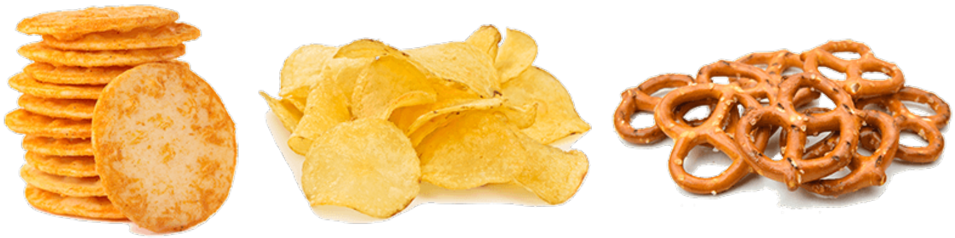

○ each week
○ each day
○ each month
○ I don't eat this

In total, how many serves of savoury snacks such as crisps, pretzels or plain/flavoured crackers do you usually eat in the timeframe selected above?

1 serve =
½ snack size packet of crisps
1 handful (30g) of salty crackers or pretzels

[slider for answer]

3. How often do you usually have sweet biscuits/cakes/ buns/ muffins/ doughnuts? Include both home-made and bought.

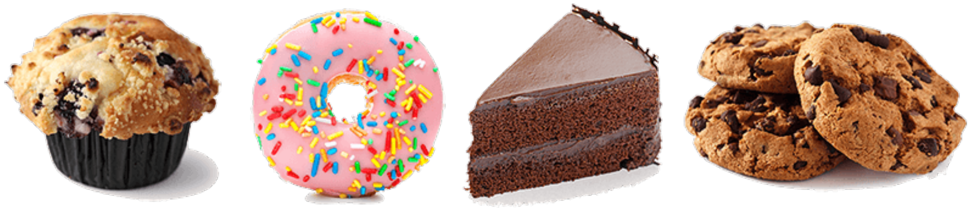

○ each week
○ each day
○ each month
○ I don't eat this

In total, how many serves of sweet biscuits/cakes/buns/muffins/doughnuts do you usually eat in the timeframe selected above?

1 serve =
2-3 (35g) sweet biscuits
1 doughnut
1 slice (40g) of plain cake or sweet bun
1 small muffin

[slider for answer]

4. How often do you usually eat savoury pastries?

This includes pies, pasties, sausage rolls, Kransky Dogs and frankfurters wrapped in pastry.

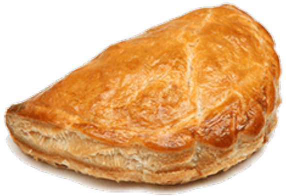

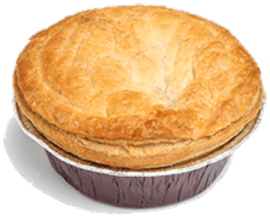

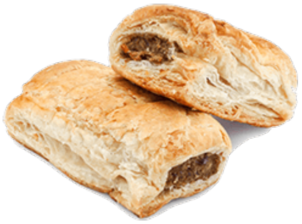

○ each week
○ each day
○ each month
○ I don't eat this

In total, how many serves of pies or savoury pastries do you usually eat in the timeframe selected above?

1 serve =
1/4 (60g) commerical meat pies or pastie
1 party size pie or sausage roll

[slider for answer]

5. How often do you usually eat snack type bars?

This includes muesli bars, fruit bars and breakfast cereal bars.

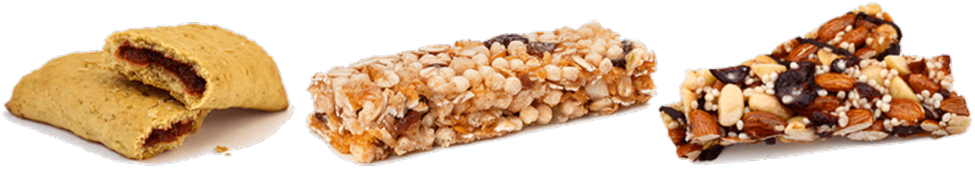

○ each week
○ each day
○ each month
○ I don't eat this

In total, how many snack type bars do you usually eat in the timeframe selected above? This includes muesli bars, fruit bars and breakfast cereal bars.

[slider for answer]

6. How often do you usually have chocolate or lollies? *This question is required.
Include all types of chocolate and both hard and soft lollies.

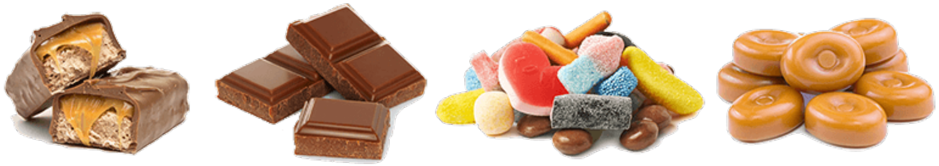

○ each week

○ each day
○ each month
○ I don't eat this

In total, how many serves of chocolate or lollies do you usually eat in the timeframe selected above?

1 serve =
½ chocolate bar
4 pieces of chocolate (25g)
5-6 (40g) lollies

[slider for answer]

7. How often do you usually have ice-cream or ice-blocks?

This includes ice-blocks, ice-cream in a bowl or ice-creams on a stick.

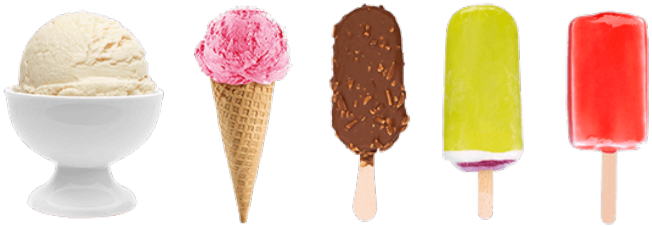

○ each week
○ each day
○ each month
○ I don't eat this

In total, how many serves of ice-cream or ice-blocks do you usually eat in the timeframe selected above? *This question is required.

1 serve =
2 scoops (60g) ice-cream
1 stick ice-cream or ice-block

[slider for answer]

What do you think about snacking?

**Over the next month:**	Strongly disagree						Strongly agree
7. I want to eat fewer unhealthy snacks	○	○	○	○	○	○	○
8. I plan to eat fewer unhealthy snacks	○	○	○	○	○	○	○
9. I intend (plan) to eat fewer unhealthy snacks	○	○	○	○	○	○	○

What snacking strategies do you already have?

**I already have clear plans about…**	Strongly disagree	Strongly agree

Deleted: If I eat healthier snacks... ... [3]

Deleted: Strongly disagree ... [4]

Deleted: Not at all true

Deleted: Barely true

Deleted: Mostly true

Deleted: Exactly true

Formatted Table

1. How I will change my unhealthy snacking habits	O	O	O	O	O	O	O
2. When I will change my unhealthy snacking habits	O	O	O	O	O	O	O
3. When I need to watch out so that I keep choosing healthy snacks	O	O	O	O	O	O	O
4. What to do in situations that make it hard to avoid unhealthy snacks (times when it is hard not to eat unhealthy snacks)	O	O	O	O	O	O	O
5. How to get back on track when I have eaten unhealthy snacks	O	O	O	O	O	O	O

How do you feel about changing your unhealthy snacking?

**I am sure that…**	Strongly disagree						Strongly agree
1. I can avoid eating unhealthy snacks for the next month	O	O	O	O	O	O	O
**I am certain that I can avoid eating unhealthy snacks even if…**							
2. Friends or family are eating unhealthy snacks	O	O	O	O	O	O	O
3. I am bored	O	O	O	O	O	O	O
4. I am craving an unhealthy snack	O	O	O	O	O	O	O

Deleted: Barely true

Deleted: Mostly true

Deleted: Exactly true

Formatted Table

Deleted: Not at all true

Nobody is perfect. Sometimes we have trouble sticking to our plans. Imagine you have started eating unhealthy snacks again. How confident are you about changing this habit?

I am certain I could go back to eating healthy snacks…

5. Even after I ate 1 unhealthy snack	O	O	O	O	O	O	O
6. Even after a few days of eating unhealthy snacks	O	O	O	O	O	O	O
7. Even after a week of eating unhealthy snacks	O	O	O	O	O	O	O

Please read the nutritional panel below to answer the following questions. The panel is information on the back of a container or ice cream.

**Nutrition Facts**	
Serving Size	1/2 cup
Servings per container	4
Amount per serving	
Calories 250	Fat Cal 120
	%DV
**Total Fat** 13g	20%
Sat Fat 9g	40%
**Cholesterol** 28mg	12%
**Sodium** 55mg	2%
**Total Carbohydrate** 30g	12%
Dietary Fiber 2g	
Sugars 23g	
**Protein** 4g	8%

* Percent Daily Values (DV) are based on a 2,000 calorie diet. Your daily values may be higher or lower depending on your calorie needs.

Ingredients: Cream, Skim Milk, Liquid Sugar, Water, Egg Yolks, Brown Sugar, Milkfat, Peanut Oil, Sugar, Butter, Salt, Carrageenan, Vanilla Extract.

1. If you eat the entire container, how many calories will you eat?

2. If you are allowed to eat 60 grams of carbohydrates as a snack, how much ice cream could you have?

3. Your doctor advises you to reduce the amount of saturated fat in your diet. You usually have 42g of saturated fat each day, which includes one serving of ice cream. If you stop eating ice cream, how many grams of saturated fat would you be consuming each day?

4. If you usually eat 2,500 calories in a day, what percentage of your daily value of calories will you be eating if you eat one serving?

Pretend that you are allergic to the following substances: penicillin, peanuts, latex gloves, and bee stings.

5. Is it safe for you to eat this ice cream?
6. *[Ask only if patient responds 'no' to question 5]*: Why not?

Moved (insertion) [4]

Formatted: Font:14 pt

Great! It looks like you're ready to **snack smarter**! The next step is to come up with a plan.

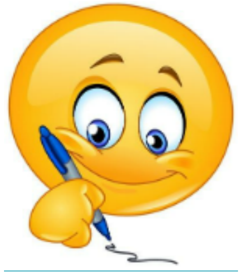

Let's get started!

Formatted: Centered

The next section depends on intervention condition.

Screen 4a – Volitional help sheet condition

Smart snacking

Step 1: Snack moments

Sometimes we snack because we are hungry, but there are lots of other reasons too.

Think about your snacks in the **last week**. Below is a list of '**snack moments**.' These are times when people tend to choose unhealthy snacks or eat too much.

Choose **3** snack moments from the list that happened to you the **most often** in the last week.

I often eat unhealthy snacks when...

The snack is in front of me	I have a craving	I am bored	I am tired	I start with one piece but then keep eating	I am in front of a TV or computer
Someone offers me the snack	People around me are eating	I am happy	I am drinking alcohol	I am busy or stressed	I am about to go to bed
I am drinking tea or coffee	I am sad	I want to reward myself	it is part of a celebration or special event	it is my day off	I have arrived home

Formatted: Font:Bold

Next

Step 2: The 'key' snack moment

Below are your top 3 snack moments.

Some snack moments will be more important than others. Choose the **1** that you would be **happiest** to change.

I really want to change my habit of snacking when...

Deleted: often snack when

I am bored	I am sitting in front of a TV or computer	people around me are eating

Next

Step 3: Make a plan

Great! Your key snack moment is:
Eating unhealthy snacks when I am **bored [example text]**.

The last step is to come up with a plan! **Choose the solution that you think will work best for you**. Drag it into the space on the right

I will go outside for a walk	I will listen to music	I will chat to someone for 5 minutes
I will drink tea	I will do a chore or task	I will eat a smaller amount
I will drink a large glass of water	I will eat a piece of fruit	I will take the food out of the packet and put it on a plate
	I will eat fresh vegetables and dip	

If I want a snack because I am bored, I will...

Next

Deleted: most important
Deleted: was
Deleted: snacking becaus
Deleted: e you are
Deleted: <sp>
Formatted: Font:18 pt
Formatted: Centered

Step 4: Your plan is almost ready!

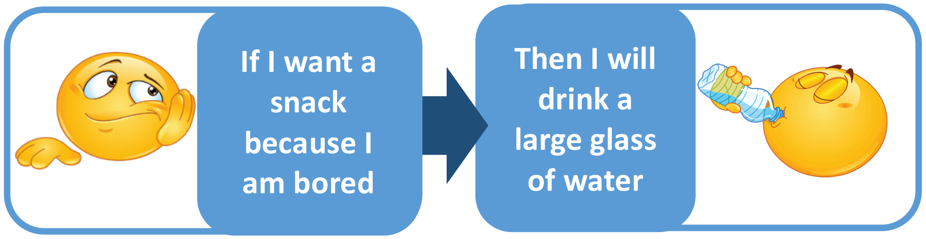

Imagine how your plan might feel. **When do you feel bored?**
Sometimes we want unhealthy snacks when we :

- waiting for a friend to arrive
- on a long train or bus trip
- watching TV
- doing long repetitive tasks

If this happens, and you want an unhealthy snack, do you think you could drink a large glass of water instead?

How hard do you think it will be to do this plan for the next month?

Very easy Very hard

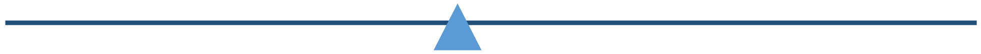

Deleted: Here are some examples

Deleted: The final step is to make sure the plan is realistic.

Next

[If score for previous question is <7/10]

Your score shows that this plan may be hard for you.

Let's make a plan that is a bit easier!

I will go outside for a walk	I will listen to music	I will chat to someone for 5 minutes
I will drink tea	I will do a chore or task	I will eat a smaller amount
I will drink a large glass of water	I will eat a piece of fruit	I will take the food out of the packet and put it on a plate
	I will eat fresh vegetables and dip	

If I want a snack because I am bored, I will…

Moved down [2]: Step 4: Your plan is almost ready!

Deleted:

Deleted: quite

Deleted: Would you like to choose an easier plan? We suggest you choose a different solution that you think will be easier to follow.

Deleted: <sp>

Formatted: Font:14 pt

Formatted: Font:14 pt, Bold, Font color: Red

Formatted: Font:18 pt

Formatted: Centered

[If score for previous question is ≥7/10]

Step 4: Your plan is almost ready!

Moved (insertion) [2]

Your smart snacking plan

Well done! Try to remember this plan for the **next month**. Say it **3** times to yourself. You can also make a copy of your plan at the end of this survey.

Deleted: You may also like to write the plan down or take a screenshot.

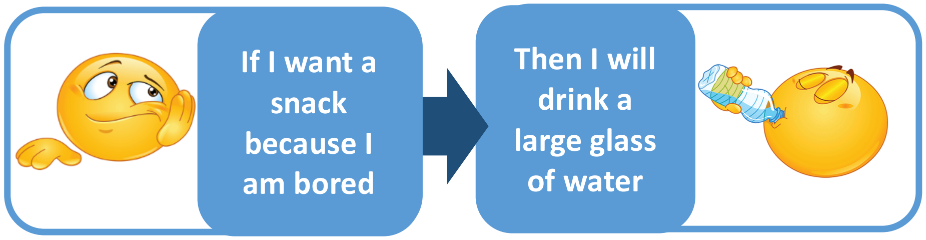

Take a screenshot

Next

Screen 4b – Detailed planning condition (active control)

Smart snacking

Your healthy snacking plan

We want you to plan how you will change your unhealthy snacking behaviour each day because forming plans has been shown to improve snacking habits.

You are free to choose how you do this but we want you to formulate your plans in as much detail as possible.

Please pay attention to the **situations** in which you will implement (carry out) these plans. Focus on situations when you are not hungry but find yourself snacking.

Please enter your plan below
[Text box – e.g. When I am bored and hungry I will remember to drink a large glass of water first.]

How hard do you think it will be to do this plan for the next month?

Very easy Very hard

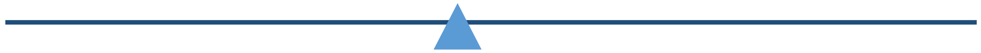

Next

Your healthy snacking plan

Well done! Try to remember this plan for the **next month**. Say it a few times to yourself. You may also like to write the plan down or take a screenshot. You can also download this plan as a PDF at the end of the survey.

[Example: When I am bored and hungry I will remember to drink a large glass of water first.]

Take a screenshot | **Next**

Screen 4c – Helpful hints sheet (passive control)

Smart snacking

Read the 'healthy snacks' fact sheet below.

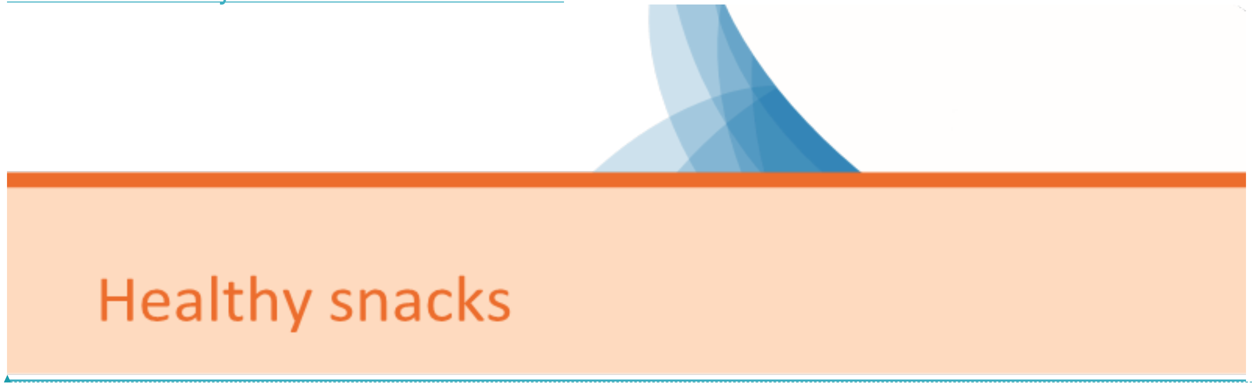

Eating the right balance of healthy foods can help you live well. Snacks can help you meet your daily nutrition needs, but it's important to make healthy choices and watch your portions to manage your weight.

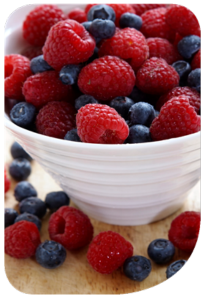

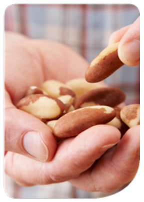

The snacks below are good choices. Try these in the recommended portions.

Fruit

» Fresh fruit: one apple/pear/orange/ large peach/large nectarine/small banana; three apricots/mandarins; two kiwifruit/plums; one cup of grapes/ cherries/melon
» Canned fruit in natural juice (drained): one cup of fruit salad/apricots/peaches
» Dried fruit: one tablespoon of sultanas; six prunes; four whole dried apricots

Formatted: Font:(Default) Times New Roman, 12 pt

Formatted: Font:(Default) Arial, 13.5 pt, Font color: R,G,B (67,70,77)

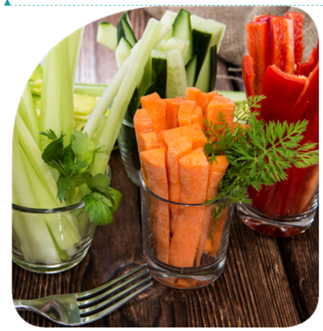

Vegetables

» ½ cup no-added-salt baked beans
» One steamed cob of corn
» Small can (125g) corn kernels
» Vegetable sticks: celery, carrot, capsicum, cucumber or snow peas with 1 slice (20g) of low-fat cheese or one tablespoon of hummus, tomato salsa or tzatziki yoghurt dip
» Celery boats with two tablespoons of low-fat cream cheese or no-added-salt peanut butter
» A handful of cherry tomatoes

Dairy and alternatives

» 100g of low-fat flavoured yoghurt or 200g of diet/natural/plain varieties. Flavour natural yoghurt with passionfruit, fresh/frozen berries, one teaspoon of honey, nuts or seeds
» 250ml of low-fat plain milk/soy milk with added calcium
» Medium cappuccino/café latte/flat white coffee with low-fat milk/soy milk

Breads and cereals

» One slice of dense grainy/seeded bread (plain or toasted) with a thin spread of margarine or avocado or low-fat ricotta or cottage cheese or hummus or chutney, with sliced tomato and sprouts
» One thin slice of raisin/wholegrain fruit bread, fresh or toasted, with a thin spread of margarine or no-added-salt peanut butter/natural nut spread or 100% fruit jam
» Half a wholegrain English muffin grilled with one slice of low-fat cheese and a sliced tomato
» One small wholemeal pita pocket or wrap with grated carrot and grated low-fat cheese
» Four rice/corn thins spread with a ¼ of an avocado and topped with sliced cucumber and tinned salmon
» Ten plain wholegrain rice crackers with low-fat cream cheese or hummus or salsa
» Two wholegrain crispbread with cottage cheese or avocado and tomato

Nuts and seeds

» 30 grams (a small handful) of plain, raw unsalted nuts: mixed, macadamias, walnuts, almonds, cashews, brazil, hazelnuts, pecans, pistachios
» 30 grams (a small handful) of pumpkin or sunflower seeds

Formatted: Font:(Default) Arial, 13.5 pt, Font color: R,G,B (67,70,77)

Lean meat and alternatives

» A mini can of tuna/salmon/sardines served in half a capsicum or a lettuce cup

» A hard-boiled egg

Grains

» One cup of air-popped popcorn served plain, or sprinkled with cinnamon/ paprika/chilli

Muesli and nut bars

Muesli and nut bars are a 'sometimes' snack as they can be high in kilojoules, added fat and sugar. Check the nutrition information panel to choose higher fibre/ healthier options and eat these only occasionally.

Homemade snacks

Homemade snacks can be a healthy choice but they can still be high in kilojoules. Eat small portions of these only occasionally.

» Cakes, biscuits, muffins and slices baked at home can be made healthier by using olive, canola, rice bran, grapeseed or sunflower margarines or oils; less sugar, wholemeal flour, oats, nuts, seeds and fruit.

When shopping, try to limit buying foods that are high in saturated fat, sugar, salt (sodium), such as:

- sweet biscuits and some crackers
- cakes, muffins and slices,
- pastries
- chocolates and confectionary
- potato crisps, corn chips and other salty snacks
- processed and pre-packaged snack foods
- fast foods and high fat takeaways

Drinks

It's important to stay hydrated throughout the day. Water is the best drink, but some other alternatives include:

» mineral or soda water flavoured with sliced lemon/lime/frozen berries/ cucumber/fresh mint

» black/oolong/green/herbal tea

» coffee or decaffeinated coffee with a dash of skim or low-fat milk.

As an occasional substitute, diet cordial or diet soft drink may add variety without extra sugar or kilojoules.

What is your plan? How hard do you think it will be to do this plan for the next month?

Very easy — Very hard

Formatted: Font:(Default) Arial, 13.5 pt, Font color: R,G,B (67,70,77)

Formatted: Font:(Default) Arial, 13.5 pt, Font color: R,G,B (67,70,77)

Formatted: Font:(Default) Helvetica, 14 pt, Font color: R,G,B (64,64,64)

Deleted: Click the link below to read the 'healthy snacks' fact sheet. This will help you come up with a plan to choose healthier snacks [see attachment]. Once you have read the fact sheet, please answer the following question:

Deleted: How hard do you think it will be to do this plan for the next month?

Next

Screen 5 – Tool evaluation

Great work! Now you have a plan to try out for the next month.

We would like to ask a few more questions before you finish.

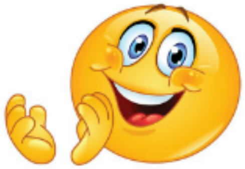

How hard was the tool to use?

Not at all hard	A little hard	Somewhat hard	Very hard	Extremely hard
○	○	○	○	○

Deleted: difficult

Deleted: Extremely

Deleted: Quite a bit

Deleted: A little bit

Deleted: Not at all

Screen 6 – Health literacy measures

1. How confident are you filling out medical forms by yourself?

Not at all	A little bit	Somewhat	Quite a bit	Extremely
○	○	○	○	○

Deleted: Extremely
Deleted: Quite a bit
Deleted: A little bit
Deleted: Not at all

On a scale from very difficult to very easy, how easy would you say it is to:

Moved (insertion) [3]

	Very difficult	Fairly difficult	Fairly easy	Very easy	Don't know
1. Judge when you may need to get a second opinion from another doctor?	○	○	○	○	○
2. Use information the doctor gives you to make decisions about your illness?	○	○	○	○	○
3. Find information on how to manage mental health problems like stress or depression?	○	○	○	○	○
4. Judge if the information on health risks in the media is reliable?	○	○	○	○	○
5. Find out about activities that are good for your mental well-being?	○	○	○	○	○
6. Understand information in the media on how to get healthier?	○	○	○	○	○

7.
8.
9.
10.

11.
12.

Deleted: Page Break

Moved up [4]: Please read the nutritional panel below to answer the following questions. The panel is information on the back of a container or ice cream.

If you eat the entire container, how many calories will you eat?

If you are allowed to eat 60 grams of carbohydrates as a snack, how much ice cream could you have?

Your doctor advises you to reduce the amount of saturated fat in your diet. You usually have 42g of saturated fat each day, which includes one serving of ice cream. If you stop eating ice cream, how many grams of saturated fat would you be consuming each day?

If you usually eat 2,500 calories in a day, what percentage of your daily value of calories will you be eating if you eat one serving?

Pretend that you are allergic to the following substances: penicillin, peanuts, latex gloves, and bee stings.
Is it safe for you to eat this ice cream?
[Ask only if patient responds 'no' to question 5]: Why not?
Page Break

Formatted: Font:14 pt

Moved up [3]: On a scale from very difficult to very easy, how easy would you say it is to: ... [5]

That's it! Good luck with your smart snacking plan!

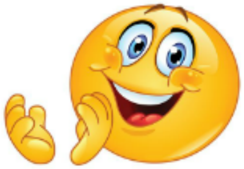

Before you leave, would you like to print or download a copy of your plan?

Print or download plan
to download a PDF select 'print as PDF' when you select the printer.

[space below will show a copy of the plan OR a copy of the tips sheet]

Next

Formatted: Font:(Default) Arial, 13.5 pt, Font color: R,G,B (67,70,77)

Formatted: Font:14 pt

Before we finish, it is very important that you have a copy of your plan!

You can write it down, print it out or save it as a PDF or screenshot.

Press the 'back' button if you need to make a copy. If you have a copy, click 'yes' below.

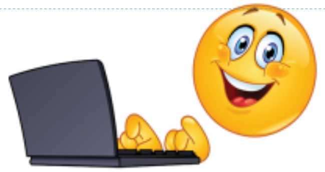

Yes, I have a copy of my plan!

Formatted: Font:Not Bold

Formatted: Font:14 pt

Formatted: Font:Not Bold

Deleted: Next

Formatted: Font color: Background 1

Formatted: Font color: Background 1

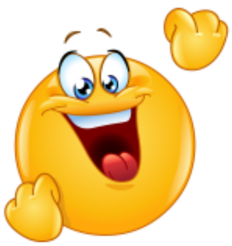

That's it! Good luck with your smart snacking plan!

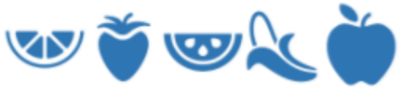

Thank you for completing this survey.

We will check in with you in **one month** to see how your plan is going.

Formatted: Font:(Default) Arial, 14 pt

Deleted: That is the end of the survey, thank you for your participation. ... [6]

Formatted: Font:Not Bold

Page 2: [1] Moved to page 2 (Move #1)	Julie Ayre	8/7/17 2:30:00 PM

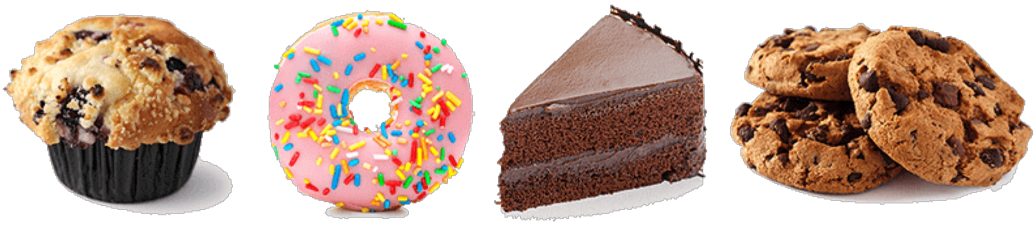

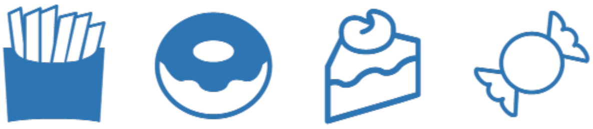

Smart Snacking

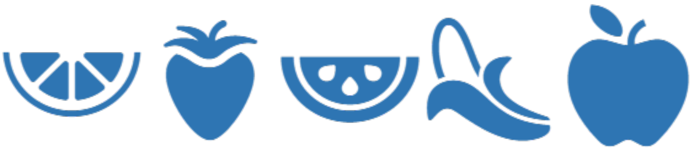

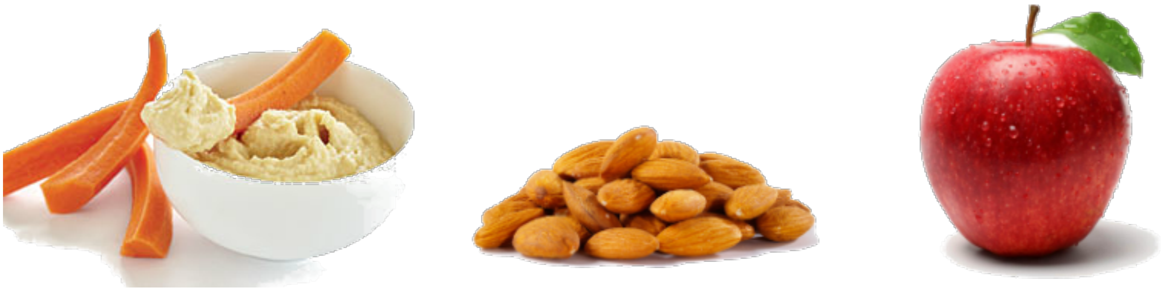

Page 12: [2] Deleted	Julie Ayre	8/7/17 2:56:00 PM

Snacks in the last week

	Not at all		
1. In the last week, too what extent (how much) have you eaten healthy snacks? (e.g. apple, banana, dried fruit)	○	○	○
2. In the last week, to what extent (how much) have you eaten unhealthy snacks? (e.g. chocolate, crisps, cake)	○	○	○

Page 19: [3] Deleted	Julie Ayre	8/7/17 2:59:00 PM

**If I eat healthier snacks…**	Not at all true	Barely true	Mostly true	Exactly true
1.I will feel healthier overall	○	○	○	○
2. I will feel better physically	○	○	○	○
3. I will have more energy	○	○	○	○
4. I will feel less hungry between meals	○	○	○	○
5. It will improve my body weight	○	○	○	○

Page 19: [4] Deleted	Julie Ayre	8/7/17 2:59:00 PM

	Strongly disagree						Strongly agree
6. Unhealthy snacking will make it harder to stay a healthy weight	○	○	○	○	○	○	○

Page 42: [5] Moved to page 41 (Move #3)	Julie Ayre	8/7/17 3:17:00 PM

On a scale from very difficult to very easy, how easy would you say it is to:

	Very difficult	Fairly difficult	Fairly easy	Very easy	Don't know
1. Judge when you may need to get a second opinion from another doctor?	○	○	○	○	○
2. Use information the doctor gives you to make decisions about your illness?	○	○	○	○	○
3. Find information on how to manage mental health problems like stress or depression?	○	○	○	○	○
4. Judge if the information on health risks in the media is reliable?	○	○	○	○	○
5. Find out about activities that are good for your mental well-being?	○	○	○	○	○
6. Understand information in the media on how to get healthier?	○	○	○	○	○

Page 45: [6] Deleted	Julie Ayre	8/7/17 3:18:00 PM

That is the end of the survey, thank you for your participation.

You will be sent a planning reminder in one week.

After one month you will be asked to complete the final survey.

If you would like to receive a copy of the results of this study, please email the study office at julie.ayre@sydney.edu.au

Screen 1 – Project Title Screen

Smart snacking: An online planning tool

(Follow-up survey)

A study conducted by the School of Public Health at the University of Sydney

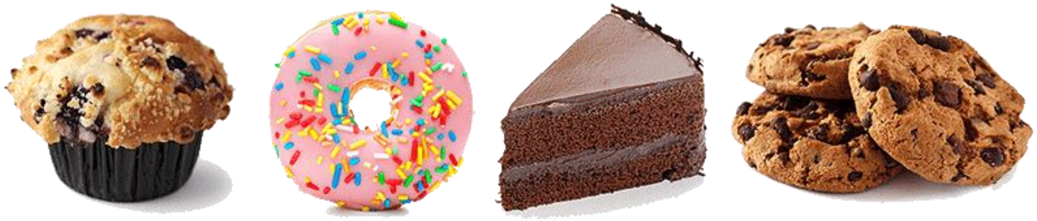

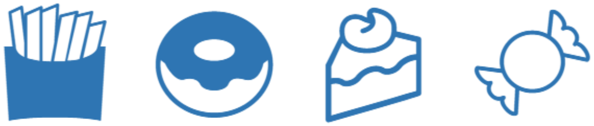

Smart Snacking

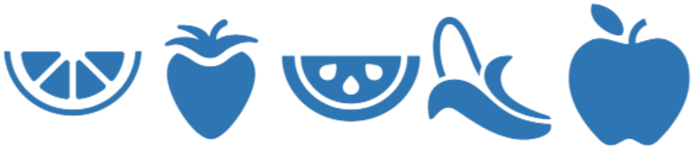

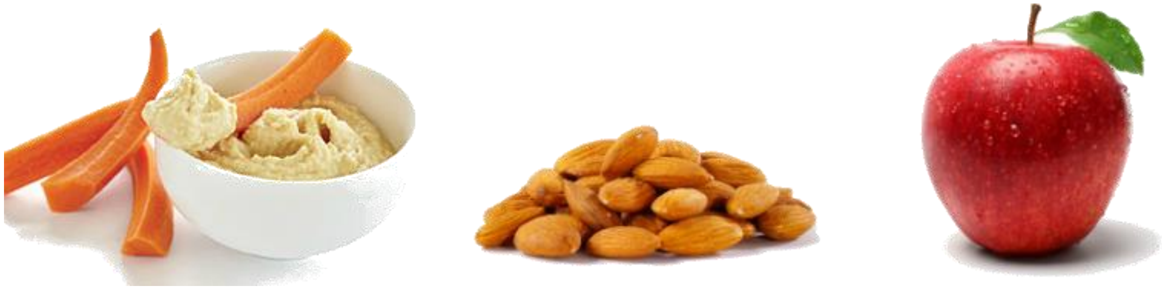

THE UNIVERSITY OF SYDNEY

Smart snacking

Snacks are important because they keep us going until the next meal. Sometimes though, we eat too many, or choose snacks that are unhealthy.

Even though each snack is usually small, over time the snacks add up. This can make us gain weight.

Smart snacking means choosing nutritious, healthy snacks that give you energy until the next meal.

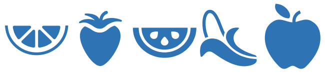

Which snacks are healthy?

Healthy snacks are low in kilojoules, fat, salt and sugars. These include fresh fruit, vegetables with dip, small amounts of dried fruit or nuts, yoghurt, coffee made with low fat milk, raisin toast, rice crackers and corn thins.

What are unhealthy snacks?

Unhealthy snacks are high in kilojoules, fat, salt and sugars. These include biscuits, cheese crackers, cakes, muffins, pastries, chocolate, lollies, potato chips, hot chips, French fries, some muesli bars and large coffees made with full cream milk.

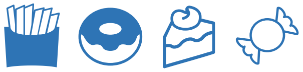

Next

What are your snacking habits?

Before we get started on smart snacking, we'd like to know a little more about your snacking habits over the last month. For each question please answer by selecting a radio button.

Snacking habits

	Strongly disagree						Strongly agree
1. I eat unhealthy snacks frequently (all the time)	○	○	○	○	○	○	○
2. I eat unhealthy snacks automatically (without thinking)	○	○	○	○	○	○	○
3. I eat unhealthy snacks without having to consciously remember (When I eat unhealthy snacks I have not made an active decision to eat them)	○	○	○	○	○	○	○
4. I feel weird if I do not eat unhealthy snacks	○	○	○	○	○	○	○
5. I eat unhealthy snacks without thinking	○	○	○	○	○	○	○
6. It would require effort not to eat unhealthy snacks	○	○	○	○	○	○	○
7. Unhealthy snacks belong to (are part of) my routine (what I usually do)	○	○	○	○	○	○	○
8. I start eating unhealthy snacks before I realise I'm doing it	○	○	○	○	○	○	○
9. I would find it hard not to eat unhealthy snacks	○	○	○	○	○	○	○
10. I don't need to think about unhealthy snacks	○	○	○	○	○	○	○
11. Unhealthy snacking is typical (normal) for me	○	○	○	○	○	○	○
12. I've been eating unhealthy snacks for a long time	○	○	○	○	○	○	○

Snacks in the last week

Think about your snacking habits in the last week. To what extent have you eaten (how true is it that you have eaten):

	Not at all						Very much
1. healthy snacks? (e.g. apple, banana, dried fruit)	O	O	O	O	O	O	O
2. unhealthy snacks? (e.g. chocolate, crisps, cake)	O	O	O	O	O	O	O

Yesterday's snacks

Which snacks did you eat yesterday? Do not include food eaten during breakfast, lunch or dinner.

- O Hot chips, potato gems or French fries
- O crisps or corn chips, crackers with cheese
- O Muffins, cake or doughnuts
- O biscuits
- O Pretzels
- O Pies, pasties or sausage rolls
- O Muesli bars, fruit bars, breakfast cereal bars
- O Chocolate
- O Lollies
- O Ice cream or ice blocks
- O Coffee with full cream milk
- O Coffee with skim milk
- O tea
- O Vita weat or Ryvita
- O Raisin toast
- O Apple or pear
- O Banana, mango
- O Orange or grapefruit
- O Kiwi fruit, mandarins
- O Yoghurt
- O Cherries, peaches or plums
- O Grapes or berries
- O Watermelon, melon
- O Carrot, cucumber or capsicum
- O Other fruit
- O Dip (e.g. hommos), cottage cheese or peanut butter
- O Nuts
- O Popcorn, rice crackers or corn thins
- O yoghurt
- O Other

Snack diary

1. How often do you usually eat oven baked potato gems/chips/hashbrowns, hot chips/French fries, wedges or fried potatoes?

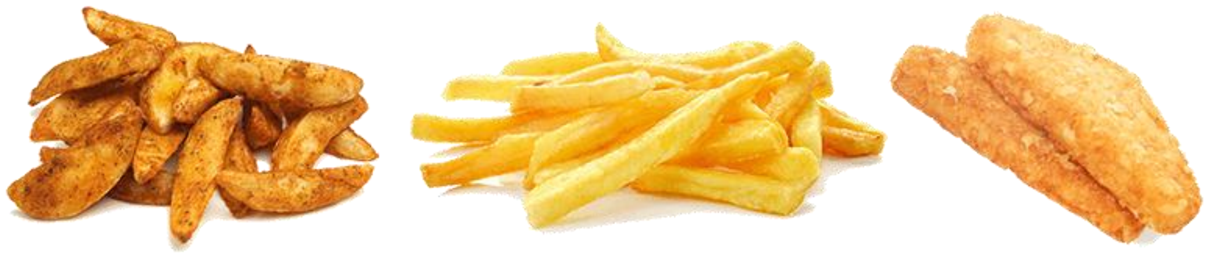

○ each week
○ each day
○ each month
○ I don't eat this

In total, how many serves of potato gems/chips/hashbrowns, hot chips/French fries, wedges or fried potatoes do you usually eat in the timeframe selected above?

1 serve =
12 fried hot chips
1 cup (60g) potato gems/hashbrowns, or wedges

[slider for answer]

2. How often do you usually eat savoury snacks such as crisps, pretzels or plain/flavoured crackers?

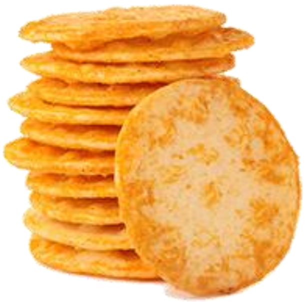
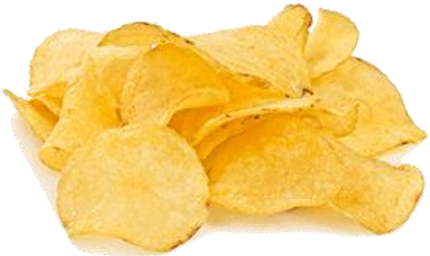
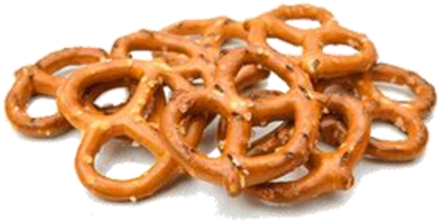

○ each week
○ each day
○ each month
○ I don’t eat this

In total, how many serves of savoury snacks such as crisps, pretzels or plain/flavoured crackers do you usually eat in the timeframe selected above?

1 serve =
½ snack size packet of crisps
1 handful (30g) of salty crackers or pretzels

[slider for answer]

3. How often do you usually have sweet biscuits/cakes/ buns/ muffins/ doughnuts? Include both home-made and bought.

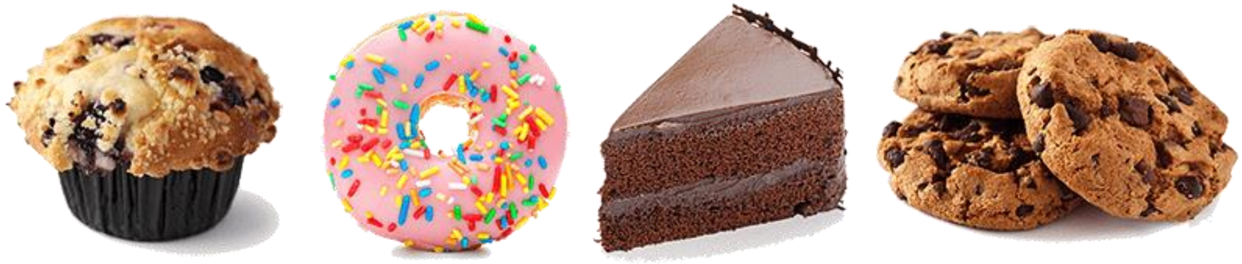

○ each week
○ each day
○ each month
○ I don’t eat this

In total, how many serves of sweet biscuits/cakes/buns/muffins/doughnuts do you usually eat in the timeframe selected above?

1 serve =
2-3 (35g) sweet biscuits
1 doughnut
1 slice (40g) of plain cake or sweet bun
1 small muffin

[slider for answer]

4. How often do you usually eat savoury pastries?

This includes pies, pasties, sausage rolls, Kransky Dogs and frankfurters wrapped in pastry.

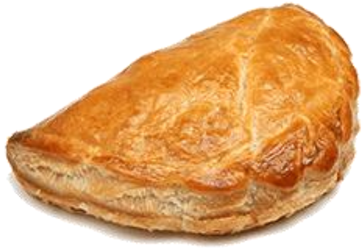 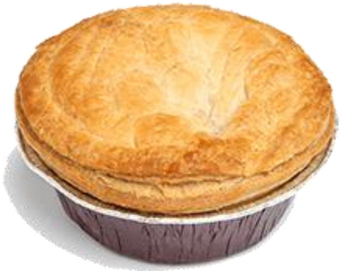 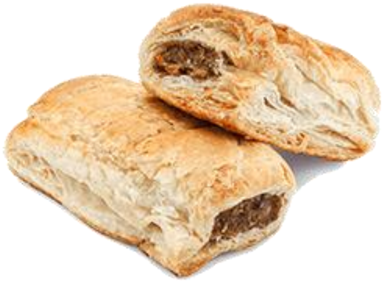

○ each week
○ each day
○ each month
○ I don’t eat this

In total, how many serves of pies or savoury pastries do you usually eat in the timeframe selected above?

1 serve =
1/4 (60g) commerical meat pies or pastie
1 party size pie or sausage roll

[slider for answer]

5. How often do you usually eat snack type bars?

This includes muesli bars, fruit bars and breakfast cereal bars.

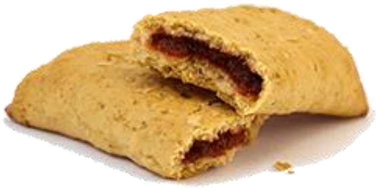 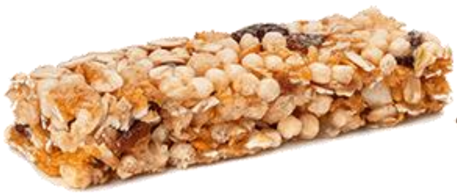 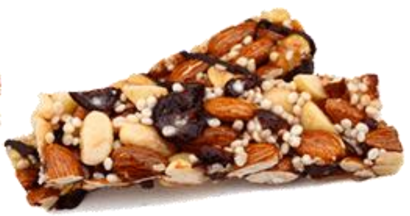

○ each week
○ each day
○ each month
○ I don’t eat this

In total, how many snack type bars do you usually eat in the timeframe selected above? This includes muesli bars, fruit bars and breakfast cereal bars.

[slider for answer]

6. How often do you usually have chocolate or lollies? *This question is required.
Include all types of chocolate and both hard and soft lollies.

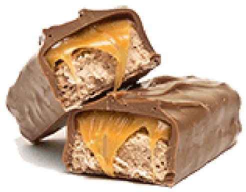 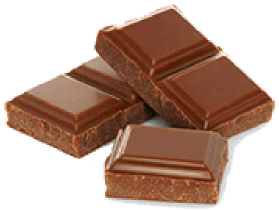 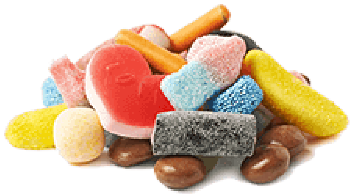 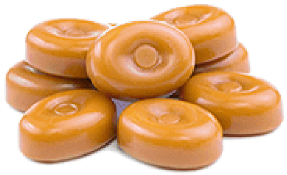

○ each week

○ each day
○ each month
○ I don’t eat this

In total, how many serves of chocolate or lollies do you usually eat in the timeframe selected above?

1 serve =
½ chocolate bar
4 pieces of chocolate (25g)
5-6 (40g) lollies

[slider for answer]

7. How often do you usually have ice-cream or ice-blocks?

This includes ice-blocks, ice-cream in a bowl or ice-creams on a stick.

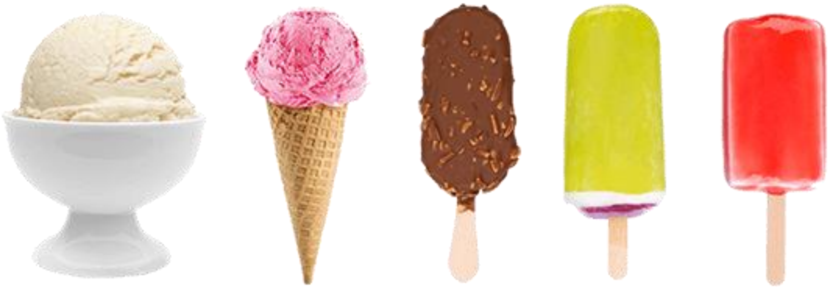

○ each week
○ each day
○ each month
○ I don’t eat this

In total, how many serves of ice-cream or ice-blocks do you usually eat in the timeframe selected above? *This question is required.

1 serve =
2 scoops (60g) ice-cream
1 stick ice-cream or ice-block

[slider for answer]

What do you think about snacking?

**Over the next month:**	Strongly disagree						Strongly agree
7. I want to eat fewer unhealthy snacks	O	O	O	O	O	O	O
8. I plan to eat fewer unhealthy snacks	O	O	O	O	O	O	O
9. I intend (plan) to eat fewer unhealthy snacks	O	O	O	O	O	O	O

What snacking strategies do you already have?

**I have clear plans about…**	Strongly disagree	Strongly agree

1. How I will change my unhealthy snacking habits	○	○	○	○	○	○	○
2. When I will change my unhealthy snacking habits	○	○	○	○	○	○	○
3. When I need to watch out so that I keep choosing healthy snacks	○	○	○	○	○	○	○
4. What to do in situations that make it hard to avoid unhealthy snacks (times when it is hard not to eat unhealthy snacks)	○	○	○	○	○	○	○
5. How to get back on track when I have eaten unhealthy snacks	○	○	○	○	○	○	○

How do you feel about changing your unhealthy snacking?

**I am sure that…**	Strongly disagre e						Strongly agree
1. I can avoid eating unhealthy snacks for the next month	○	○	○	○	○	○	○
**I am certain that I can avoid eating unhealthy snacks even if…**							
2. Friends or family are eating unhealthy snacks	○	○	○	○	○	○	○
3. I am bored	○	○	○	○	○	○	○
4. I am craving an unhealthy snack	○	○	○	○	○	○	○

Nobody is perfect. Sometimes we have trouble sticking to our plans. Imagine you have started eating unhealthy snacks again. How confident are you about changing this habit?

I am certain I could go back to eating healthy snacks…

5. Even after I ate 1 unhealthy snack	○	○	○	○	○	○	○
6. Even after a few days of eating unhealthy snacks	○	○	○	○	○	○	○
7. Even after a week of eating unhealthy snacks	○	○	○	○	○	○	○

How did you go with your snacking plan?

During the last month…	Strongly disagree						Strongly agree
I often thought about my plan to reduce the number of (eat less) unhealthy snacks I ate each day	○	○	○	○	○	○	○
I constantly (often) kept count of how many unhealthy snacks I ate to make sure it was not too much	○	○	○	○	○	○	○
I reminded myself to make sure I wasn't having too many unhealthy snacks	○	○	○	○	○	○	○
I tried my best to be consistent with my plan (stick with my plan) to eat less unhealthy snacks	○	○	○	○	○	○	○
I really tried to reduce the number of (eat less) unhealthy snacks I ate each day	○	○	○	○	○	○	○

That is the end of the survey, thank you for your participation.

If you would like more information about the study, please click here.

If you would like to receive a copy of the results of this study, please email the study office at julie.ayre@sydney.edu.au

Screen 1 – Project Title Screen

Smart snacking: An online planning tool
(Follow-up survey)

A study conducted by the School of Public Health at the University of Sydney

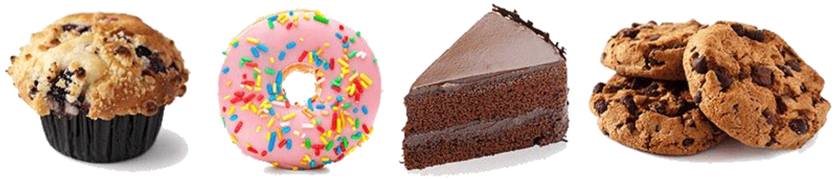

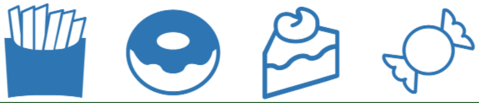

Smart Snacking

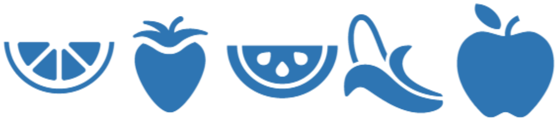

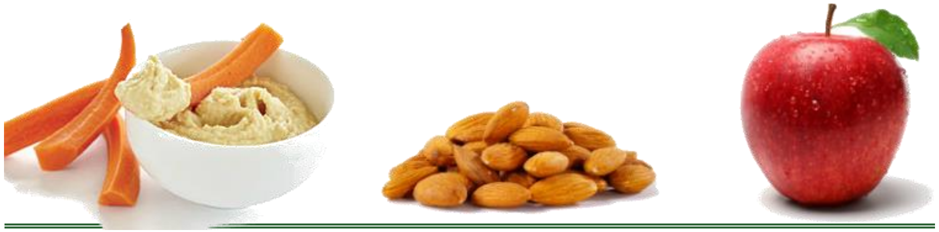

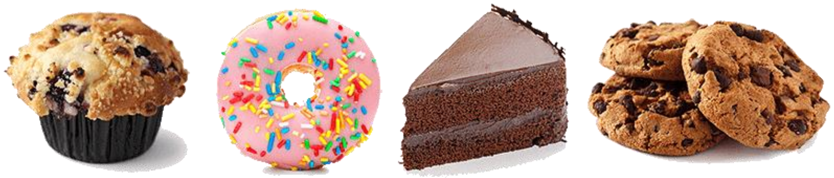

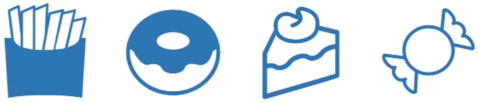

Smart Snacking

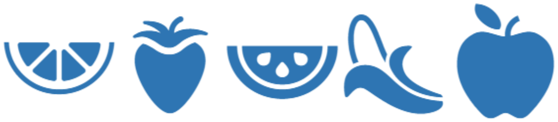

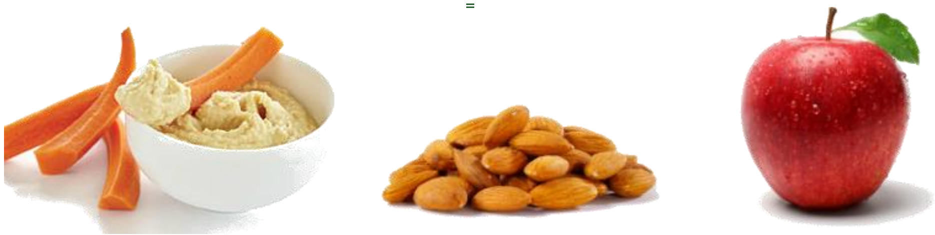

Formatted: Centered

Smart snacking

Snacks are important because they keep us going until the next meal. Sometimes though, we eat too many, or choose snacks that are unhealthy.

Even though each snack is usually small, over time the snacks add up. This can make us gain weight.

Smart snacking means choosing nutritious, healthy snacks that give you energy until the next meal.

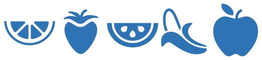

Which snacks are healthy?

Healthy snacks are low in kilojoules, fat, salt and sugars. These include fresh fruit, vegetables with dip, small amounts of dried fruit or nuts, yoghurt, coffee made with low fat milk, raisin toast, rice crackers and corn thins.

What are unhealthy snacks?

Unhealthy snacks are high in kilojoules, fat, salt and sugars. These include biscuits, cheese crackers, cakes, muffins, pastries, chocolate, lollies, potato chips, hot chips, French fries, some muesli bars and large coffees made with full cream milk.

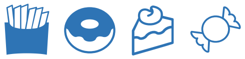

Next

What are your snacking habits?

Before we get started on smart snacking, we'd like to know a little more about your snacking habits over the last month. For each question please answer by selecting a radio button.

Snacking habits

	Strongly disagree						Strongly agree
1. I eat unhealthy snacks frequently (all the time)	O	O	O	O	O	O	O
2. I eat unhealthy snacks automatically (without thinking)	O	O	O	O	O	O	O
3. I eat unhealthy snacks without having to consciously remember (When I eat unhealthy snacks I have not made an active decision to eat them)	O	O	O	O	O	O	O
4. I feel weird if I do not eat unhealthy snacks	O	O	O	O	O	O	O
5. I eat unhealthy snacks without thinking	O	O	O	O	O	O	O
6. It would require effort not to eat unhealthy snacks	O	O	O	O	O	O	O
7. Unhealthy snacks belong to (are part of) my ~~(daily, weekly, monthly)~~ routine (what I usually do)	O	O	O	O	O	O	O
8. I start eating unhealthy snacks before I realise I'm doing it	O	O	O	O	O	O	O
9. I would find it hard not to eat unhealthy snacks	O	O	O	O	O	O	O
10. I don't need to think about unhealthy snacks	O	O	O	O	O	O	O
11. Unhealthy snacking is typical (normal) for me	O	O	O	O	O	O	O
12. I've been eating unhealthy snacks for a long time	O	O	O	O	O	O	O

Snacks in the last week

Think about your snacking habits in the last week. To what extent have you eaten (how true is it that you have eaten):

	Not at all						Very much
1. healthy snacks? (e.g. apple, banana, dried fruit)	O	O	O	O	O	O	O
2. unhealthy snacks? (e.g. chocolate, crisps, cake)	O	O	O	O	O	O	O

Yesterday's snacks

Which snacks did you eat yesterday? Do not include food eaten during breakfast, lunch or dinner.

- O Hot chips, potato gems or French fries
- O ~~Crackers,~~ crisps or corn chips, crackers with cheese
- O Muffins, cake or doughnuts
- O biscuits
- O Pretzels
- O Pies, pasties or sausage rolls
- O Muesli bars, fruit bars, breakfast cereal bars
- O Chocolate
- O Lollies
- O Ice cream or ice blocks
- O Coffee with full cream milk
- O Coffee with skim milk
- O tea
- O Vita weat or Ryvita
- O Raisin toast
- O Apple or pear
- O Banana, mango
- O Orange or grapefruit
- O Kiwi fruit, mandarins
- O Yoghurt
- O Cherries, peaches or plums
- O Grapes or berries
- O Watermelon, melon
- O Carrot, cucumber or capsicum
- O Other fruit
- O Dip (e.g. hommos), cottage cheese or peanut butter
- O Nuts
- O Popcorn, rice crackers or corn thins
- O yoghurt
- O Other

~~Snacks in the last week~~

	~~Not at all~~						~~Very much~~
~~1. In the last week, too what extent (how much) have you eaten healthy snacks? (e.g. apple, banana, dried fruit)~~	~~O~~	~~O~~	~~O~~	~~O~~	~~O~~	~~O~~	~~O~~
~~2. In the last week, to what extent (how much) have you eaten unhealthy snacks? (e.g. chocolate, crisps, cake)~~	~~O~~	~~O~~	~~O~~	~~O~~	~~O~~	~~O~~	~~O~~

Snack diary ~~for previous week~~

1. How often do you usually eat oven baked potato gems/chips/hashbrowns, hot chips/French fries, wedges or fried potatoes?

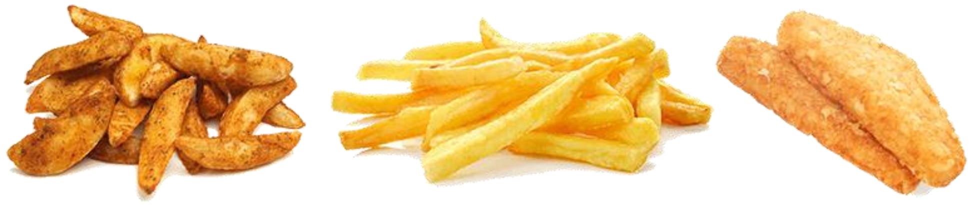

○ each week
○ each day
○ each month
○ I don't eat this

In total, how many serves of potato gems/chips/hashbrowns, hot chips/French fries, wedges or fried potatoes do you usually eat in the timeframe selected above?

1 serve =
12 fried hot chips

1 cup (60g) potato gems/hashbrowns, or wedges

[slider for answer]

2. How often do you usually eat savoury snacks such as crisps, pretzels or plain/flavoured crackers?

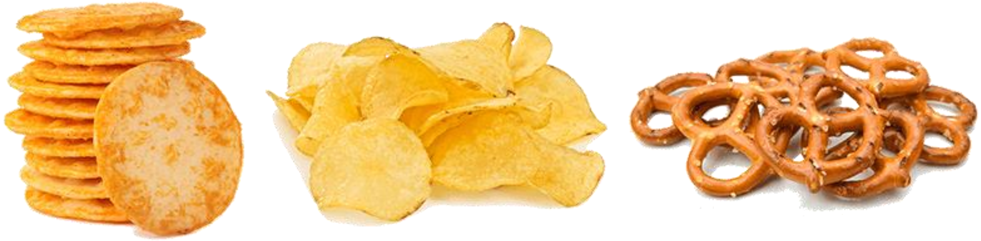

○ each week
○ each day
○ each month
○ I don't eat this

In total, how many serves of savoury snacks such as crisps, pretzels or plain/flavoured crackers do you usually eat in the timeframe selected above?

1 serve =
½ snack size packet of crisps
1 handful (30g) of salty crackers or pretzels

[slider for answer]

3. How often do you usually have sweet biscuits/cakes/ buns/ muffins/ doughnuts? Include both home-made and bought.

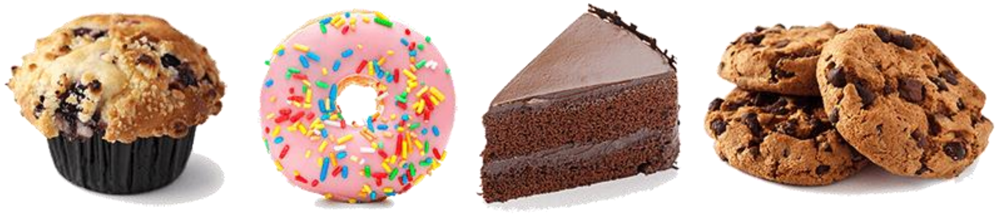

○ each week
○ each day
○ each month
○ I don't eat this

In total, how many serves of sweet biscuits/cakes/buns/muffins/doughnuts do you usually eat in the timeframe selected above?

1 serve =
2-3 (35g) sweet biscuits
1 doughnut
1 slice (40g) of plain cake or sweet bun
1 small muffin

[slider for answer]

4. How often do you usually eat savoury pastries?

This includes pies, pasties, sausage rolls, Kransky Dogs and frankfurters wrapped in pastry.

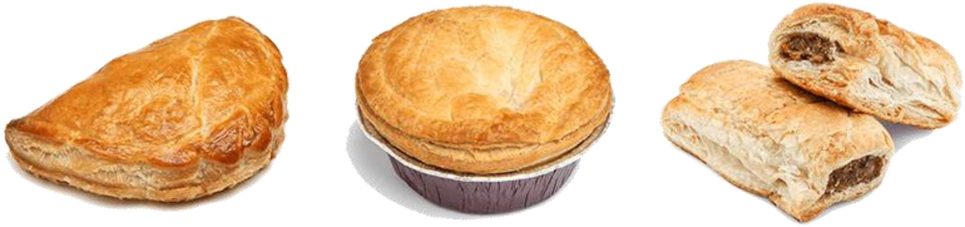

○ each week
○ each day
○ each month
○ I don't eat this

In total, how many serves of pies or savoury pastries do you usually eat in the timeframe selected above?

1 serve =
1/4 (60g) commerical meat pies or pastie
1 party size pie or sausage roll

[slider for answer]

5. How often do you usually eat snack type bars?

This includes muesli bars, fruit bars and breakfast cereal bars.

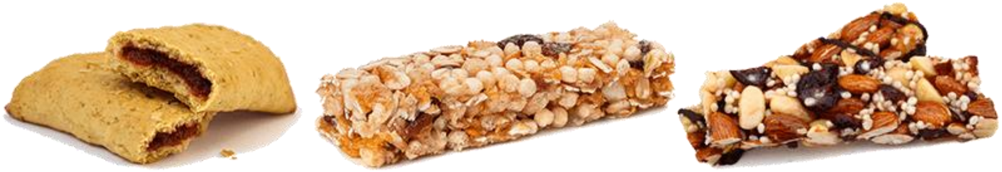

○ each week
○ each day
○ each month
○ I don't eat this

In total, how many snack type bars do you usually eat in the timeframe selected above? This includes muesli bars, fruit bars and breakfast cereal bars.

[slider for answer]

6. How often do you usually have chocolate or lollies? *This question is required.

Include all types of chocolate and both hard and soft lollies.

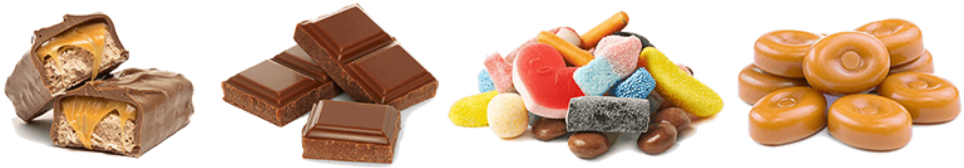

○ each week

○ each day
○ each month
○ I don't eat this

In total, how many serves of chocolate or lollies do you usually eat in the timeframe selected above?

1 serve =
½ chocolate bar
4 pieces of chocolate (25g)
5-6 (40g) lollies

[slider for answer]

7. How often do you usually have ice-cream or ice-blocks?

This includes ice-blocks, ice-cream in a bowl or ice-creams on a stick.

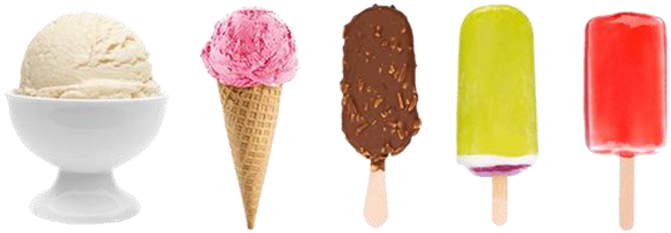

○ each week
○ each day
○ each month
○ I don't eat this

In total, how many serves of ice-cream or ice-blocks do you usually eat in the timeframe selected above? *This question is required.

1 serve =

2 scoops (60g) ice-cream

1 stick ice-cream or ice-block

[slider for answer]

What do you think about snacking?

~~**If I eat healthier snacks...**~~	~~Not at all true~~	~~Barely true~~	~~Mostly true~~	~~Exactly true~~
~~1.I will feel healthier overall~~	~~O~~	~~O~~	~~O~~	~~O~~
~~2. I will feel better physically~~	~~O~~	~~O~~	~~O~~	~~O~~
~~3. I will have more energy~~	~~O~~	~~O~~	~~O~~	~~O~~
~~4. I will feel less hungry between meals~~	~~O~~	~~O~~	~~O~~	~~O~~
~~5. It will improve my body weight~~	~~O~~	~~O~~	~~O~~	~~O~~

	~~Strongly disagree~~						~~Strongly agree~~
~~6. Unhealthy snacking will make it harder to stay a healthy weight~~	~~O~~	~~O~~	~~O~~	~~O~~	~~O~~	~~O~~	~~O~~

**Over the next month:**	Strongly disagree	Strongly agree

7. I want to eat fewer unhealthy snacks	O	O	O	O	O	O	O
8. I plan to eat fewer unhealthy snacks	O	O	O	O	O	O	O
9. I intend (plan) to eat fewer unhealthy snacks	O	O	O	O	O	O	O

What snacking strategies do you already have?

I have clear plans about...	Strongly disagreeNot at all true	Barely true	Mostly true	Exactly true			Strongly agree
1. How I will change my unhealthy snacking habits	O	O	O	O	O	O	O
2. When I will change my unhealthy snacking habits	O	O	O	O	O	O	O
3. When I need to watch out so that I keep choosing healthy snacks	O	O	O	O	O	O	O
4. What to do in situations that make it hard to avoid unhealthy snacks (times when it is hard not to eat unhealthy snacks)	O	O	O	O	O	O	O
5. How to get back on track when I have eaten unhealthy snacks	O	O	O	O	O	O	O

Formatted Table

How do you feel about changing your unhealthy snacking?

I am sure that…	Strongly disagree~~Not at all true~~	~~Barely true~~	~~Mostly true~~	~~Exactly true~~			Strongly agree
1. I can avoid eating unhealthy snacks for the next month	O	O	O	O	O	O	O
**I am certain that I can avoid eating unhealthy snacks even if…**							
2. Friends or family are eating unhealthy snacks	O	O	O	O	O	O	O
3. I am bored	O	O	O	O	O	O	O
4. I am craving an unhealthy snack	O	O	O	O	O	O	O

Nobody is perfect. Sometimes we have trouble sticking to our plans. Imagine you have started eating unhealthy snacks again. How confident are you about changing this habit?

I am certain I could go back to eating healthy snacks…							
5. Even after I ate 1 unhealthy snack	O	O	O	O	O	O	O
6. Even after a few days of eating unhealthy snacks	O	O	O	O	O	O	O
7. Even after a week of eating unhealthy snacks	O	O	O	O	O	O	O

Formatted Table

How ~~was~~ did you go with your snacking plan?

**During the last month…**	Strongly disagree						Strongly agree
I often thought about my plan to reduce the number of (eat less) unhealthy snacks I ate each day	○	○	○	○	○	○	○
I constantly (often) kept count of how many unhealthy snacks I ate to make sure it was not too much	○	○	○	○	○	○	○
I reminded myself to make sure I wasn't having too many unhealthy snacks	○	○	○	○	○	○	○
I tried my best to be consistent with my plan (stick with my plan) to eat less unhealthy snacks	○	○	○	○	○	○	○
I really tried to reduce the number of (eat less) unhealthy snacks I ate each day	○	○	○	○	○	○	○

That is the end of the survey, thank you for your participation.

If you would like more information about the study, please click here.

Formatted: Font: 14 pt, Bold

If you would like to receive a copy of the results of this study, please email the study office at julie.ayre@sydney.edu.au

Survey Sampling International (SSI) recruitment details

Overview (corresponds to Q215/Q217 in Section D of HREC application):

SSI is a market research company that has an extensive database of participants who are willing to be involved in online research. Initial contact will be made by SSI by using their database to approach potential participants who meet the eligibility criteria. Participants listed on their database have already indicated a willingness to participate in online research.

Obtaining consent (corresponds to Q217 in Section D of HREC application):

If participants agree and are interested in being part of the study, they will be directed to an online Participant Information Sheet, consent information and then the survey.

Withdrawal from the study (corresponds to Q218 in Section D of HREC application):

Participants are free to withdraw from the study at any time. After reading the online Participant Information Sheet, potential participants will be able elect not to proceed to the survey. It will be outlined in the Participation Information Sheet that there will be no consequences to the individual should they wish to withdraw, and that they are able to withdraw from the survey at any point. In addition, participants who proceed to the survey and then discontinue survey completion part way through will be treated as though consent has been withdrawn, and their responses will not be used.

Participant reimbursement (corresponds to Q219/221 in Section D of HREC application):

Participants will not receive any financial or other reward from the University of Sydney. SSI uses a points system whereby points are earned for completion of surveys. These points can be redeemed for items such as gift vouchers, donations to charities or cash. The points represent modest compensation for the half an hour or so that participants will forgo by completing the survey.

The invitation and incentive policy of SSI is in line with European Society for Opinion and Marketing Research (ESOMAR) guidelines. The survey incentives are in the form of points. They are based on survey length rather than being offered for particular surveys, and there is no minimum participation requirement. An average 25-minute survey gives the participant 75 points for non-fraudulent completion, assuming the respondent qualifies for the survey. 75 points is equal to $1.50, and can be used to enter the prize draws. SSI practices an active anti-fraud policy, whereby they verify the respondent's genuine participation and identity prior, during and after each survey as well as during the rewards claim process. To be able to complete a survey and to collect the reward, the respondent needs to quality for a survey, i.e. profile match and pass all quality checks. The value of the reward in itself is not competitive with the minimum wage per hour, to avoid encouraging fake responses. This ensures that the completed surveys are quality-assured and less likely to be influenced by 'incentives-skewed bias'.

THE UNIVERSITY OF SYDNEY

HUMAN RESEARCH ETHICS COMMITTEE FORM

Please Note:

This form was created via the University's online system (IRMA) and the information provided is recorded in the University's research office database.

This information is used to assess the ethics submission under the National Health and Medical Research Council's (NHMRCs) National Statement on Ethical Conduct in Human Research (2007) by the University Ethics Committee and its expert advisers, including the RPAH Clinical Trials Subcommittee.

Sign off by researchers is provided online in IRMA and will not be displayed in this document.

ADMINISTRATIVE DETAILS

Title: Online planning tool for unhealthy snacking

Chief Investigator: Prof Kirsten McCaffery

Primary Faculty/Department: School of Public Health: Public Health; Faculty of Medicine and Health

Investigators: McCaffery Kirsten; Ayre Julie; Bonner Carissa;

Grants linked:

External Authorities:

Additional Information:

please see attached cover letter

QUESTIONNAIRE

LIST OF ATTACHED DOCUMENTS

Date Uploaded	Type	Document Name
21/08/2017	Cover Letter/Correspondence	cover letter

THE UNIVERSITY OF SYDNEY

School of Public Health
Faculty of Medicine

ABN 15 211 513 464

PROFESSOR Kirsten McCaffery
School of Public Health

Room 301F
Edward Ford Building (A27)
The University of Sydney
NSW 2006 AUSTRALIA
Telephone: +61 2 9351 7220
Email: Kirsten.mccaffery@sydney.edu.au
Web: http://www.sydney.edu.au/

Friday August 18, 2017

Human Research Ethics Committee (Category B Chair)

Re: Project No. 2017/662 – "Online planning tool for unhealthy snacking"

Thank you for your response to the above study application reviewed by the Human Ethics Review Committee on 16 August 2017.

Please find attached a detailed response to each point.

We look forward to hearing back from you.

Sincerely,

KJ McCaffery

Professor Kirsten McCaffery
School of Public Health
University of Sydney

Modification – reminder emails

1) The response cover letter states "We have included 2 additional reminder emails, such that there are no reminder emails at baseline, 1 week and 2 weeks. Previously there was only a reminder email at 1 week".

a. Please clarify when the reminders will happen. How can a reminder be sent at baseline? Are 3 reminders being sent after the initial baseline survey and after the follow-up survey?

Reminders will be sent at baseline (within 1 week of submitting the baseline survey), and then again 1 week and 2 weeks later. There are a total of 3 reminders, as shown in the diagram below:

**Week**	**Description**
1 (baseline)	Participant completes baseline survey and will receive a reminder email before the end of the first week
2	Participant receives reminder email before the end of the second week
3	Participant receives reminder email before the end of the third week
4 (follow-up survey)	Participant completes follow up survey

Data management and storage

2) As specified in the Research Data Management Policy 2014, it is mandatory that University staff and research students prepare and implement a Research Data Management Plan (RDMP) for all research projects using the University's RDMP tool. To ensure data is stored securely and protected against loss or damage during and after completion of your project, the data storage arrangements detailed in your RDMP must be in line with the Research Data Management Guidelines. Please confirm that you will adhere to this requirement.

We can confirm that we will adhere with the above requirement. We have submitted an RDMP for 'Online planning tool for unhealthy snacking' (RDMP ID: rdmp_5152).

Modification requests

3) While the modifications requested in the response have been reviewed at the discretion of the Chair, please note that in future, requests to modify approved projects or projects pending approval need to be submitted via a modification form in IRMA and reviewed by the Modification Review Committees (MRCs). Please visit the Human Ethics website for more information on modifications and the submission deadlines for the MRCs.

This has been noted and will be submitted correctly for future modifications.

Research Integrity & Ethics Administration
Human Research Ethics Committee

Monday, 28 August 2017

Assoc Prof Kirsten McCaffery
School of Public Health: Public Health; Sydney Medical School

email: kirsten.mccaffery@sydney.edu.au

Dear Kirsten,

The University of Sydney Human Research Ethics Committee (HREC) has considered your application.

After consideration of your response to the comments raised your project has been approved.

Approval is granted for a period of four years from **28/08/2017** to **28/08/2021**.

Project title: **Online planning tool for unhealthy snacking**

Project no.: **2017/622**

First Annual Report due: **28/08/2018**

Authorised Personnel: **McCaffery Kirsten; Ayre Julie; Bonner Carissa;**

Documents Approved:

Date Uploaded	Version number	Document Name
14/08/2017	Version 2	Participant information statement vs 2 (clean)
14/08/2017	Version 2	Smart snacking planning tool baseline v2 (clean)
14/08/2017	Version 2	Smart snacking planning tool followup v2 (clean)
22/06/2017	Version 1	Debriefing information sheet for participants
22/06/2017	Version 1	Baseline questionnaire (includes PIS and consent form)
22/06/2017	Version 1	Followup questionnaire

Condition/s of Approval

- Research must be conducted according to the approved proposal.
- An annual progress report must be submitted to the Ethics Office on or before the anniversary of approval and on completion of the project.
- You must report as soon as practicable anything that might warrant review of ethical approval of the project including:
  - Serious or unexpected adverse events (which should be reported within 72 hours).
  - Unforeseen events that might affect continued ethical acceptability of the project.
- Any changes to the proposal must be approved prior to their implementation (except where an amendment is undertaken to eliminate *immediate* risk to participants).
- Personnel working on this project must be sufficiently qualified by education, training and experience for their role, or adequately supervised. Changes to personnel must be reported and approved.

THE UNIVERSITY OF SYDNEY

- Personnel must disclose any actual or potential conflicts of interest, including any financial or other interest or affiliation, as relevant to this project.
- Data and primary materials must be retained and stored in accordance with the relevant legislation and University guidelines.
- Ethics approval is dependent upon ongoing compliance of the research with the *National Statement on Ethical Conduct in Human Research*, the *Australian Code for the Responsible Conduct of Research*, applicable legal requirements, and with University policies, procedures and governance requirements.
- The Ethics Office may conduct audits on approved projects.
- The Chief Investigator has ultimate responsibility for the conduct of the research and is responsible for ensuring all others involved will conduct the research in accordance with the above.

This letter constitutes ethical approval only.

Please contact the Ethics Office should you require further information or clarification.

Sincerely,

Associate Professor Michael Skilton
Chair, Health Review Committee (Low Risk)

The University of Sydney HRECs are constituted and operate in accordance with the National Health and Medical Research Council's (NHMRC) National Statement on Ethical Conduct in Human Research (2007) and the NHMRC's Australian Code for the Responsible Conduct of Research (2007).
